# Supplementary material for: Brain Re-Irradiation Or Chemotherapy: a phase II randomised trial of re-irradiation and chemotherapy in patients with recurrent glioblastoma (BRIOChe) – protocol for a multi-centre open-label randomised trial
Source: BMJ Open. 2024 Mar 8;14(3):e078926. doi: 10.1136/bmjopen-2023-078926 (PMC11145639; doi:10.1136/bmjopen-2023-078926)
Supplement: Supplementary data [file bmjopen-2023-078926supp001.pdf]

SUPPLEMENTARY FILE 1 – Full inclusion exclusion criteria

Inclusion criteria	Exclusion criteria
1. Histologically proven diagnosis of GBM with consistent molecular pathology, based on original pathology.	1. Pregnant (positive pregnancy test) or lactating
2. First recurrence of GBM, with contrast enhancing disease, following primary treatment (i.e. no previous systemic therapy or re-irradiation for recurrence).	2. Critical normal brain structures treated above usual tolerance during initial radiotherapy
3. Agreement of a Consultant Neuro-Radiologist within multi-disciplinary team meeting that imaging changes are in keeping with recurrence and not pseudo-progression.	3. Recurrence with leptomeningeal disease or only leptomeningeal disease
4. Randomisation must be performed within 21 days of the MRI that confirms recurrence.	4. Recurrence defined by non-enhancing disease only
5. ≥6 months since completion of primary radiotherapy	5. More than three enhancing lesions present on MRI or multi-focal recurrence
6. Prior history of standard dose, conventionally fractionated CNS radiotherapy	6. IDH1/2 mutant tumours on original pathology (to avoid unbalance between arms).
7. As a minimum patients will have completed at least two weeks of temozolomide, concurrent with their original radiotherapy.	7. GBM with known features of PXA, BRAF mutations or 1p19q co-deletion (on original pathology or updated pathology if available)
• Up to and including three enhancing lesions:<br>○ In cases of a single recurrent enhancing lesion:<br>▪ Predicted re-irradiation GTV<75cm^3 and<br>▪ Maximum diameter of enhancing disease must be ≤6cm.<br>○ In cases of multiple (i.e. two or three) discrete recurrent enhancing lesions:<br>▪ The total (i.e. combined) predicted re-irradiation GTV must be <50cm^3 and lesions must be clustered in a similar brain region such that PTVs are anticipated to be adjacent or overlapping and<br>▪ Maximum diameter of the combined enhancing disease, across all enhancing lesions (including any gaps between), must be ≤6cm.	8. Prior invasive malignancy (except non-melanomatous skin cancer), unless disease free for a minimum of one year.

8. Karnofsky Performance Status 70+	9. Severe active co-morbidity making patient unsuitable for chemotherapy or re-irradiation
9. Adequate hematologic, renal, and hepatic function	10. Prior allergic reaction to nitrosoureas
10. Patients who have had surgery for first recurrence may also be included provided there is residual enhancing disease on the immediate post-operative MRI or if enhancing disease develops on subsequent follow-up imaging, provided no prior systemic therapy or re-irradiation for recurrence has been given.	11. Any recognised genetic syndromes causing sensitivity to radiotherapy
11. Patients must have recovered from the prior effects of therapy	12. Patient unwilling/ unable to attend for follow up in the radiotherapy centre
12. Medical history and physical examination, including CNS examination, must be performed within 14 days prior to randomisation.	13. Contra-indication to MRI or gadolinium
13. Female participants of child-bearing potential must agree to be pregnancy screened.	14. Previous radiotherapy dose distribution unavailable
14. Patients must be able to swallow oral medication.	15. Previous systemic therapy or re-irradiation for recurrent GBM
15. Patients must be able to provide study-specific informed consent.	16. Spinal or infratentorial disease
16. Age 18 or over.	
17. Patients should be able to start treatment within 21 days of randomisation and must start within 28 days of randomisation.	

Coeliac disease is usually considered a contra-indication to lomustine as there is wheat within the capsule. For patients with recurrent GBM, the individual clinician may decide whether or not this is a relevant exclusion criteria.
