# Supplementary material for: Brain Re-Irradiation Or Chemotherapy: a phase II randomised trial of re-irradiation and chemotherapy in patients with recurrent glioblastoma (BRIOChe) – protocol for a multi-centre open-label randomised trial
Source: BMJ Open. 2024 Mar 8;14(3):e078926. doi: 10.1136/bmjopen-2023-078926 (PMC11145639; doi:10.1136/bmjopen-2023-078926)
Supplement: Supplementary data [file bmjopen-2023-078926supp002.pdf]

Delete this line, then print on Trust/Hospital headed paper

Participant ID:	Initials:
Date of Birth:	NHS/Hospital Number:
EudraCT Number: 2019-004053-91	Principal Investigator:

BRIOChe

SAMPLE

PARTICIPANT CONSENT FORM

Please <u>initial</u> each box

1. I confirm that I have read and understood the information sheet for the above study and have had the opportunity to ask questions. ☐

2. I understand that my participation in this study is voluntary and that I am free to withdraw at any time without my medical care or legal rights being affected. I understand that even if I withdraw from the above study, the data and samples collected from me will be used in analysing the results of the study and in some cases further information about any unwanted effects of my treatment may need to be collected by the study team. ☐

3. I understand that my healthcare records may be looked at by authorised individuals from the study team, regulatory bodies or Sponsor in order to check that the study is being carried out correctly. ☐

4. I agree to allow any information or results arising from this study to be used for healthcare and/or further medical research upon the understanding that my identity will remain anonymous wherever possible. This includes the use of my radiotherapy data and imaging data for future analysis. ☐

5. I understand that if I lose capacity, further information about any unwanted effects of my treatment may need to be collected by the study team.

6. If selected, I agree to take part in a semi-structured interview on my experiences of participating in the trial and my health-related quality of life, where a member of the research team may approach me directly to schedule a suitable day and time. I agree to these interviews being audio recorded.

7. I agree to consent to my anonymised trial data to be viewed if required by 3rd parties outside the UK / EU etc.

8. I agree to a copy of this Consent Form being sent to the Clinical Trials Research Unit.

9. I agree that my GP, or any other doctor treating me, will be notified of my participation in this study.

10. I agree to take part in the study.

The following points are **OPTIONAL.**

SAMPLE

Title: Consent for potential tissue collection and use in future research (if you do not wish to give this permission do not initial the boxes – you can still participate in the trial)

Please <u>initial</u> each box

	Yes	No
I give my permission for stored tumour tissue sample that was removed during my operation and was not needed for routine diagnosis and treatment to be collected and used for future translational research purposes as described in the information sheet for the above trial if this becomes relevant in the future.		
I understand how the tissue sample will be collected, that giving samples is voluntary and that I'm free to withdraw my approval for use of the samples at any time without giving a reason and without my medical care or legal rights being affected.		

Patient:
Signature……………………………………………………………………………………

Name (block capitals)……………………………………………………………………

Date……………………………………………………………………………………………

Investigator:

I have explained the study to the above named patient and he/she has indicated his/her willingness to participate.

Signature……………………………………………………………………………………

Name (block capitals)……………………………………………………………………

Date……………………………………………………………………………………………

(If used)Translator:

Signature……………………………………………………………………………………

Name (block capitals)……………………………………………………………………

Date……………………………………………………………………………………………

(1 copy for patient; 1 for the CTRU; 1 held in patient notes;

SAMPLE
