# Supplementary material for: Brain Re-Irradiation Or Chemotherapy: a phase II randomised trial of re-irradiation and chemotherapy in patients with recurrent glioblastoma (BRIOChe) – protocol for a multi-centre open-label randomised trial
Source: BMJ Open. 2024 Mar 8;14(3):e078926. doi: 10.1136/bmjopen-2023-078926 (PMC11145639; doi:10.1136/bmjopen-2023-078926)
Supplement: Author's manuscript [file bmjopen-2023-078926.draft_revisions.pdf]

BMJ Open

BMJ Open is committed to open peer review. As part of this commitment we make the peer review history of every article we publish publicly available.

When an article is published we post the peer reviewers' comments and the authors' responses online. We also post the versions of the paper that were used during peer review. These are the versions that the peer review comments apply to.

The versions of the paper that follow are the versions that were submitted during the peer review process. They are not the versions of record or the final published versions. They should not be cited or distributed as the published version of this manuscript.

BMJ Open is an open access journal and the full, final, typeset and author-corrected version of record of the manuscript is available on our site with no access controls, subscription charges or pay-per-view fees (http://bmjopen.bmj.com).

If you have any questions on BMJ Open's open peer review process please email info.bmjopen@bmj.com

BMJ Open

Brain Re-Irradiation Or Chemotherapy: a phase II randomised trial of re-irradiation and chemotherapy in patients with recurrent glioblastoma (BRIOChe): Protocol for a multi-centre open label randomised trial

Journal:	*BMJ Open*
Manuscript ID	bmjopen-2023-078926
Article Type:	Protocol
Date Submitted by the Author:	16-Aug-2023

Complete List of Authors:	Hudson, Eleanor; University of Leeds, Leeds Institute of Clinical Trials Research<br>Noutch, Samantha; University of Leeds, Leeds Institute of Clinical Trials Research<br>Webster, Joanne; University of Leeds, Leeds Institute of Clinical Trials Research<br>Brown, Sarah; University of Leeds, Clinical Trials Research Unit<br>Boele, Florien; University of Leeds, Leeds Institute of Medical Research at St James's; University of Leeds, Leeds Institute of Health Sciences<br>Al-Salihi, Omar; Guy's and St Thomas' Hospitals NHS Trust<br>Baines, Helen; Mount Vernon Cancer Centre, National Radiotherapy Trials QA (RTTQA) Group<br>Bulbeck, Helen; brainstrust,<br>Currie, Stuart; Leeds General Infirmary, Department of Radiology<br>Fernandez, Sharon; University of Leeds, Leeds Institute of Medical Research at St James's<br>Hughes, Jane; University of Leeds, Leeds Institute of Medical Research at St James's<br>Lilley, John; Leeds Institute of Cancer and Pathology, Department of Medical Physics<br>Smith, Alexandra; University of Leeds, Leeds Institute of Clinical Trials Research<br>Parbutt, Catherine; Leeds Teaching Hospitals NHS Trust, Pharmacy<br>Slevin, Finbar; Leeds Institute of Cancer and Pathology, Department of Clinical Oncology; University of Leeds, Leeds Institute of Medical Research at St James's<br>Short, Susan; Leeds Institute of Cancer and Pathology, Department of Clinical Oncology; University of Leeds, Leeds Institute of Medical Research at St James's<br>Sebag-Montefiore, David ; University of Leeds Faculty of Medicine and Health, Institute of Cancer and Pathology<br>Murray, Louise; Leeds Institute of Cancer and Pathology, Department of Clinical Oncology; University of Leeds, Leeds Institute of Medical Research at St James's

Keywords:	Radiation oncology < RADIOTHERAPY, RADIOTHERAPY, ONCOLOGY, Neurological oncology < NEUROLOGY

SCHOLARONE™
Manuscripts

Brain Re-Irradiation Or Chemotherapy: a phase II randomised trial of re-irradiation and chemotherapy in patients with recurrent glioblastoma (BRIOChe): Protocol for a multi-centre open label randomised trial

Authors:

Eleanor Hudson[1], Samantha Noutch[1], Joanne Webster[1], Sarah R. Brown[1], Florien W. Boele[2,3], Omar Al-Salihi[4], Helen Baines[5], Helen Bulbeck[6], Stuart Currie[7], Sharon Fernandez[2] Jane Hughes[2], John Lilley[8], Alexandra Smith[1], Catherine Parbutt[9], Finbar Slevin[2,10], Susan C. Short[2,10], David Sebag-Montefiore[2], Louise Murray[2,10]

Author affiliation:

[1]Clinical Trials Research Unit, Leeds Institute of Clinical Trials Research, University of Leeds, Leeds, United Kingdom

[2]Leeds Institute of Medical Research at St James's, University of Leeds, Leeds, United Kingdom

[3]Leeds Institute of Health Sciences, Faculty of Medicine and Health, University of Leeds, Leeds, UK

[4]Guy's and St Thomas Hospital, London, United Kingdom

[5]National Radiotherapy Trials QA (RTTQA) Group, United Kingdom

[6]Patient Advocate, United Kingdom

[7]Department of Radiology, Leeds General Infirmary, Leeds, United Kingdom

[8]Department of Medical Physics, Leeds Cancer Centre, Leeds, United Kingdom

[9]Pharmacy Department, St James's University Hospital, Leeds, United Kingdom

[10]Department of Clinical Oncology, Leeds Cancer Centre, Leeds, United Kingdom

For peer review only

Corresponding author: Dr. Louise Murray

L.J.Murray@leeds.ac.uk

Leeds Institute of Medical Research at St James's,

University of Leeds,

St James's University Hospital,

Harehills

Leeds,

LS9 7LP

+44 113 206 7854

Word count (excluding title page, abstract, references, figures and tables):

4180-128-234-115 = 3,703

For peer review only

ABSTRACT (300 word count)

Introduction

Glioblastoma (GBM) is the most common adult primary malignant brain tumour. The condition is incurable and, despite aggressive treatment at first presentation, almost all tumours recur after a median of 7 months. The aim of treatment at recurrence is to prolong survival and maintain health-related quality of life (HRQoL). Chemotherapy is typically employed for recurrent GBM, often using nitrosourea-based regimens. However, efficacy is limited, with reported median survivals between 5 and 9 months. Although less commonly used in the United Kingdom (UK), there is growing evidence that re-irradiation may produce survival outcomes at least similar to nitrosourea-based chemotherapy. However, there remains uncertainty as to the optimum approach and there is a paucity of available data, especially with regards to HRQoL. BRIOChe aims to assess re-irradiation, as an acceptable treatment option for recurrent GBM.

Methods and analysis

BRIOChe is a phase II, multi-centre, open-label, randomised trial in patients with recurrent GBM. The trial uses a Sargent's three-outcome design and will recruit 70 participants from 10-15 UK radiotherapy sites, allocated (1:1) to receive re-irradiation (35 Gy in 10 daily fractions) or nitrosourea-based chemotherapy (up to six, 6-weekly cycles). The primary endpoint is overall survival rate for re-irradiation patients at 9 months. There will be no formal statistical comparison between treatment arms for the decision-making primary analysis. The chemotherapy arm will be used for calibration purposes, to collect concurrent data to aid interpretation of results. Secondary outcomes include HRQoL, dexamethasone requirement,

For peer review only - http://bmjopen.bmj.com/site/about/guidelines.xhtml

anti-epileptic drug requirement, radiological response, treatment compliance, acute and late toxicities, progression free survival.

Ethics and dissemination

BRIOChe (ISRCTN: 16052954; registered 25/02/2021) is funded by Jon Moulton Charity Trust and obtained ethical approval from Office for Research Ethics Committees Northern Ireland (reference no. 20/NI/0070). Final trial results will be published in peer reviewed journals and adhere to the to ICMJE guidelines.

For peer review only

STRENGTHS AND WEAKNESSES (up to 5)

- BRIOChe is multi-centre randomised trial
- The trial will provide much needed data regarding the survival and quality of life impact of re-irradiation in patients with recurrent GBM
- There is a significant lack of UK trials for patients with recurrent GBM. BRIOChe provides the opportunity to establish re-irradiation as an acceptable treatment option for this patient group
- Although there is no formal comparison between treatment arms for the primary endpoint overall survival, the trial has a parallel concurrent control arm for benchmarking and to aid interpretation

For peer review only

INTRODUCTION

Glioblastoma

Glioblastoma (GBM) is the most common adult primary malignant brain tumour, with an incidence of 5/100,000 per year in England. Prognosis is poor, with an average survival of 12 to 15 months [1].

GBM is incurable, with treatment from the point of diagnosis aiming to improve survival, delay disease progression and manage symptoms/preserve HRQoL. Optimal treatment at first presentation is aggressive, including debulking surgery (if feasible), chemo-radiotherapy and adjuvant chemotherapy. However, almost all patients will relapse within the brain, with a median progression free survival (PFS) of approximately 7 months [1].

The burden placed on patients, their informal caregivers and the healthcare system as a result of neurological disability and the care requirements for patients with brain tumours is significant [2-4]. GBM is often accompanied by debilitating physical and neurological symptoms, such as paresis, sensory loss, seizures, fatigue, mood issues, and cognitive deficits [5-7]. This symptom burden can affect patients' ability to function independently and negatively impact on health-related quality of life (HRQoL) for both patients and their informal caregivers.

Recurrent glioblastoma

The optimal treatment for recurrent GBM is undefined and treatment decisions are made on a case-by-case basis, based on the site, volume and time of recurrence in relation to the original diagnosis, with consideration for patient age, co-morbidities and performance status.

In the UK, chemotherapy is commonly employed in the setting of recurrent GBM, typically using nitrosourea-based regimens. These DNA alkylating agents are highly lipophilic, and are

For peer review only

therefore able to penetrate the blood brain barrier, making them suitable for the treatment of brain tumours. Nitrosourea-combination regimens have not proven superior to single agent nitrosoureas, resulting in variations in practice as to which regimens are employed [8]. Efficacy is limited, with reported median overall survivals (OS) of between 5 and 9 months [9-17]. Side effects may include fatigue, nausea, vomiting, anorexia, constipation, paraesthesia, rash and myelosuppression. There is currently a relative lack of HRQoL data for patients receiving nitrosourea-based chemotherapy for recurrent GBM [13, 16, 18], making it difficult to determine whether the benefits of chemotherapy outweigh its burdens.

Surgery is another treatment option at point of recurrence, however, is usually reserved for a minority of excellent performance patients with well-localised disease, although high-level evidence to support its role is lacking [9, 19-21].

Re-irradiation for recurrent glioblastoma

Re-irradiation is a further treatment option for recurrent GBM, although at present it is not commonly used in the UK. A meta-analysis of re-irradiation for recurrent GBM [22] included 50 studies of re-irradiation, of which 35 employed external beam radiotherapy (EBRT; the remainder used brachytherapy). Of the patients who received EBRT, 6-month and 12-month overall survival (OS) was 70% and 34% respectively [22]. Progression free survival (PFS) was 40% and 16% at 6 and 12-months, respectively. Grade 3+ toxicity was reported in 7% of patients. Overall, only 13 of the studies were of prospective design and only 9 were considered by the authors to be of good quality. Similarly, a systematic review of re-irradiation in GBM presented comparable outcomes based on a pooled, population-weighted analysis, where mean adjusted OS following hypofractionated EBRT was 10.1 months (95% confidence interval (CI) 9.7-10.5 months) and the rate of radionecrosis was 7.1% (95% CI: 6.6-7.7%) [23]. Despite the promising outcomes reported after re-irradiation, the poor quality of existing

For peer review only

evidence highlights the urgent need for a high quality, prospective, randomised assessment of re-irradiation, including HRQoL assessment [23, 24].

Rationale for study

While the limited existing evidence suggests that re-irradiation has a potential positive impact on overall survival and HRQoL, to date, no trial has randomised patients with recurrent GBM between re-irradiation and chemotherapy. Addressing this research gap is a top priority for patients, informal caregivers, and healthcare professionals [25].

The BRIOChe phase II prospective trial will randomise patients with recurrent GBM to re-irradiation or nitrosourea-based chemotherapy. The aims will be to explore the efficacy and HRQoL impact of re-irradiation. This study will collect concurrent data for patients receiving chemotherapy for validation ('benchmarking') of outcomes. The study is not designed to statistically compare the two arms for the primary outcome of survival, rather to establish the feasibility of randomisation, provide prospectively controlled data on survival and HRQoL impacts associated with re-irradiation and guide the design of future larger scale randomised trials.

For peer review only

For peer review only - http://bmjopen.bmj.com/site/about/guidelines.xhtml

157 **METHODS AND ANALYSIS**

158

159 **Design and aim**

160 BRIOChe is a phase II, multi-centre, open-label, randomised trial in patients with recurrent 161 GBM. The aim of the study is to assess re-irradiation as an alternative treatment to 162 chemotherapy in this group of patients. BRIOChe uses a Sargent's three-outcome, single-163 stage, single-arm design, with the inclusion of a chemotherapy calibration arm for concurrent 164 data for benchmarking. The trial will recruit 70 participants from 10-15 UK radiotherapy 165 sites. Participants will be allocated, with a 1:1 ratio, to receive either re-irradiation or 166 nitrosourea-based chemotherapy (lomustine +/- procarbazine +/- vincristine).

167 The trial schema is presented in Figure 1.

168

For peer review only

Trial objectives

The primary objective of BRIOChe is to assess the proportion of participants alive at 9 months post start of treatment in the re-irradiation arm.

The key secondary objective of BRIOChe is to evaluate the effect of re-irradiation on HRQoL. Questionnaires to be completed by participants include the European Organization for Research and Treatment of Cancer (EORTC) Quality of life questionnaire core 30 (QLQ-C30) and Brain Cancer module (BN20). HRQoL will also be explored in greater detail through one-off semi-structured qualitative interviews in a subsample of patients and their informal caregivers.

Additional secondary objectives include evaluating:

- Dexamethasone requirement
- Anti-epileptic drug requirement
- Radiological response in accordance with Response Assessment in Neuro-Oncology (RANO) criteria
- Treatment compliance
- Acute (measured from randomisation to 12 weeks post end of treatment of treatment) and late toxicities (after 12 weeks post end of treatment) as per Common Terminology Criteria for Adverse Events (CTCAE V.5.0.); radionecrosis is a late toxicity of particular interest
- PFS
- OS

Study population

All inclusion/exclusion criteria must be met and written informed consent obtained prior to recruitment (Key criteria summarised in Table 1); full criteria included as Supplementary Material. The formal assessment of eligibility and informed consent may only be obtained by

For peer review only

195 the Principal Investigator (PI) or an appropriate medically qualified doctor. Patients will be
196 screened from clinic lists.

For peer review only

For peer review only - http://bmjopen.bmj.com/site/about/guidelines.xhtml

Table 1– Summary of key inclusion and exclusion criteria

Inclusion criteria	Exclusion criteria
Histologically proven diagnosis of GBM, based on original pathology.	Pregnant (positive pregnancy test) or lactating
First recurrence of GBM, with contrast enhancing disease, following primary treatment	Critical normal brain structures treated above usual tolerance during initial radiotherapy
Agreement of a Consultant Neuro-Radiologist that imaging changes are in keeping with recurrence and not pseudo-progression.	Recurrence with leptomeningeal disease or only leptomeningeal disease
≥6 months since completion of primary radiotherapy	More than three enhancing lesions present on MRI or multi-focal recurrence
Prior history of standard dose, conventionally fractionated CNS radiotherapy	IDH1/2 mutant tumours on original pathology
As a minimum, patients will have completed at least two weeks of temozolomide, concurrent with their original radiotherapy.	GBM with known features of PXA, BRAF mutations or 1p19q co-deletion (on original pathology or updated pathology if available)
Up to and including three enhancing lesions (size and volume criteria included in Supplementary Material)	Prior invasive malignancy (except non-melanomatous skin cancer), unless disease free for a minimum of one year.
Karnofsky Performance Status 70+	Severe active co-morbidity making patient unsuitable for chemotherapy or re-irradiation
Adequate hematologic, renal, and hepatic function	Prior allergic reaction to nitrosoureas
No contra-indication to lomustine	Any recognised genetic syndromes causing sensitivity to radiotherapy
Patients must be able to provide study-specific informed consent.	Contra-indication to MRI or gadolinium
Age 18 or over.	Previous radiotherapy dose distribution unavailable
	Previous systemic therapy or re-irradiation for recurrent GBM

Randomisation and recruitment

Randomisation will be performed centrally using University of Leeds Clinical Trials Research Unit (CTRU) automated 24-hour randomisation system. A computer-generated minimisation program incorporating a random element will be used to ensure the treatment groups are well balanced for the following factors:

- Age (<50 or ≥50 years)

205 • Time to randomisation from completion of previous radiotherapy (≤12 months or >12 months)

207 • Methylation status (Methylated, Unmethylated or Unknown)

208 • Randomising site

209 The intended recruitment period is 2-years (BRIOChe opened to recruitment on 22/06/2021).

210

211 **Sample size**

212 Thirty-three patients are required in the re-irradiation arm, 35 allowing for 5% drop out. With a 1:1 randomisation allocation ratio to include a calibration arm, 70 patients were required in total. The randomisation allocation ratio was adapted in a protocol amendment to 2:1 (re-irradiation: chemotherapy), to allow a higher proportion of recruited patients into the intervention arm. The subsequent sample size was estimated to be 55 patients in total, with 35 in the re-irradiation arm and approximately 20 in chemotherapy arm.

218 This study is powered to demonstrate that the treatment strategy of re-irradiation demonstrates sufficient efficacy to warrant further large-scale evaluation, based on 9-month survival rates.

221 A Sargent's three-outcome, phase II, single-stage, single-arm design will be used to determine whether re-irradiation demonstrates sufficient efficacy to warrant further evaluation [26]. The trial is designed to test the null hypothesis (H0) that the proportion of participants alive at 9 months is <25% where re-irradiation would not be deemed worthy of further investigation, against an alternative hypothesis H1 of >45% where re-irradiation would be deemed worthy of further investigation. If the proportion of participants alive at 9 months is ≥25% and ≤45%, this is an inconclusive region where neither the null or alternative hypothesis would be rejected and the decision regarding further investigation would be based on other factors. The following operating characteristics are used: type I

For peer review only - http://bmjopen.bmj.com/site/about/guidelines.xhtml

error=0.05, type II error=0.06, prob (correctly rejecting the H1) = 0.82 (eta), prob (correctly rejecting the H0) =0.79 (pi).

Treatment regimen

Re-irradiation arm: The radiotherapy prescription dose will be 35 Gy in 10 fractions, a commonly employed schedule [27-32], delivered daily Monday-Friday over two weeks. Treatment will be delivered with Intensity Modulated Radiotherapy (IMRT)/ Volumetric Modulated Arc Therapy (VMAT) or tomotherapy. Optimally, normal tissue constraints will be based on a ‘dose remaining’ approach, whereby an original organ at risk (OAR) dose (allowing for 25% repair) is subtracted from a cumulative tolerance to give a ‘dose remaining’ for re-irradiation. To save clinicians time and reduce errors in performing such calculations, the radiotherapy guidelines contain tables of ‘OAR doses remaining’ for ease of reference. Where these optimal constraints cannot be achieved, mandatory constraints are those used in the RTOG1205 trial, which also delivered a dose of 35 Gy in 10 fractions [31]. Treatment compliance data to radiotherapy, including fractions, doses, interruptions and reasons will be collected weekly during treatment.

For peer review only

Chemotherapy arm: As per usual UK practice in the setting of relapsed GBM where the interval to recurrence is insufficiently long to use re-challenge temozolomide and/or when patients have O^6-methylguanine-DNA-methyltransferase (MGMT) unmethylated tumours, chemotherapy will be lomustine-based. These drugs have been used in this setting for many years. A variety of lomustine-based regimens are used across the UK including single agent lomustine, PCV (procarbazine, lomustine and vincristine), PC (procarbazine and lomustine) or CV (lomustine and vincristine). Lomustine-based regimens will be delivered as per each centre’s standard practice. Doses will be recorded at each cycle and (based on a survey of participating centres regarding their usual practice) it is recommend that these will consist of:

- Lomustine 100-130mg/m^2, day 1, PO, on a 42 day cycle

  +/-

- Procarbazine 50-100mg/m^2, once daily on days 1-10 or days 2-11, PO, on a 42 day cycle

 +/-

- Vincristine 1.4-1.5mg/m^2, with dose capping as per the institution's usual practice, IV day 1, on a 42 day cycle.

(Dose capping is as per the institution's usual practice).

Treatment compliance data to chemotherapy will be collected from patient diaries. Dose modifications and reasons for changes will also be collected. Suggested dose modifications to chemotherapy based on haematological, renal and hepatic functioning are provided in the study protocol.

Upon further disease progression, patients may receive the alternative treatment according to local policy.

Trial assessments and follow-up

Full eligibility criteria (table 1, Supplementary Material), will be assessed and confirmed prior to randomisation. Further assessments will be conducted at multiple timepoints. An overview of data collection can be seen in table 2. Data will be collected from all participants, until progression or death. Where available, date of death will be collected for all participants.

Follow-up imaging with MRI will include T2, FLAIR (fluid attenuated inversion recovery), DWI (diffusion weighted imaging), T1 pre-gadolinium and T1 post-gadolinium sequences. In addition, patients randomised to re-irradiation will also undergo T2* Dynamic Susceptibility Contrast (DSC) perfusion as part of follow-up imaging to assist in determination of the presence of radionecrosis.

For peer review only

Table 2 – Trial assessment schedules

	Early assessments				Follow-up assessment (measured post start of treatment)									
	Eligibility	Baseline	Pre-treat RT	Pre-treat chemo	0w	1w	6w	12w	18w	24w	30w	36w	42w	48w
Informed consent	X													
Clinical assessment	X				X	X***	X	X	X	X	X	X	X	X
Performance status (Karnofsky)	X				X	X***	X	X	X	X	X	X	X	X
Bloods*	X			X	X	X	X	X	X	X	X			
Pregnancy screening**	X		X		X	X	X	X	X	X	X			
Re-irradiation					X	X								
Chemotherapy					X		X	X	X	X	X			
MRI (RANO criteria)	X						X			X				
Toxicity (CTCAE)		X			X	X***	X	X	X	X	X	X	X	X
HRQoL		X					X	X	X	X	X	X	X	X

* Bloods only for Eligibility for re-irradiation arm, with each cycle in chemotherapy arm

**only at pretreatment for re-irradiation arm, with every cycle in chemotherapy arm

*** re-irradiation arm patients only

Adverse Events (AEs), Serious Adverse Events (SAEs), Adverse Reaction (ARs), Serious Adverse Reactions (SARs) and Suspected Unexpected Serious Adverse Reactions (SUSARs) will all be collected from randomisation until the last participant follow-up or disease progression. Beyond this period only, SARs and SUSARs related to the trial treatment (and not GBM progression) will be reported if the investigator becomes aware of them until the end of trial notification. All SAEs/SARs and SUSARs related to investigation medicinal products (IMPs) will be reported to the Medicines and Healthcare products Regulatory Agency (MHRA) and sponsor. All radiotherapy related SAEs/SARs and SUSARs will be reported to the Research Ethics Committee and sponsor.

For peer review only

Statistical analysis

The statistical analysis will be conducted by University of Leeds CTRU. A full statistical analysis plan has been developed and finalised.

The trial analysis will be conducted on a Modified Intention to Treat (MITT) population [33]. MITT is defined as all participants randomised that have received at least one dose of trial treatment, analysed according to the treatment arm they were randomised to (regardless of ineligibility, non-compliance or withdrawal).

Overall survival rates at 9 months, i.e. the number and proportion of participants alive at 9 months post-start of treatment, will be calculated for the re-irradiation arm with 90% (corresponding to a 1-sided 5% significance level used in the design) and 95% confidence intervals (CIs).

Based on the 3-outcome design, the cut-off values and conclusions for the statistical test for the primary analysis of efficacy are defined as follows:

Green: If ≥13/33 patients are alive, re-irradiation demonstrates sufficient efficacy to warrant larger-scale evaluation. This is based on a 9-month OS >45% (i.e. competitive with the 'best' outcomes from prospective studies using nitrosourea-based chemotherapy [11, 14, 34].

Amber: If 11-12/33 patients are alive the decision to take re-irradiation forward will be uncertain and will be based on HRQoL and toxicity at 3 months, (this is based on 9-month OS 25%-45%; i.e. similar to outcomes from other prospective nitrosourea-based trials [13, 16, 17, 35].

Red: If ≤10/33 patients are alive re-irradiation will not be considered worthy of further investigation, based on 9-month OS <25%.

For peer review only - http://bmjopen.bmj.com/site/about/guidelines.xhtml

The chemotherapy arm is included for calibration and will be used for validation of outcomes in the re-irradiation group with respect to previous series, and validation of the assumptions used to inform the statistical cut-offs.

Summary statistics will be calculated for all domains of the EORTC QLQ-C30 and QLQ-BN20 and the overall summary score calculated, presented overall and by treatment arm for each of the follow-up time points. The difference in scores between treatment arms will be presented with corresponding 90% and 95% confidence intervals.

Summary statistics will be presented for dexamethasone requirement, anti-epileptic drug requirement, treatment compliance, and response assessment; including the proportion of participants within each clinical response status. The maximum CTCAE grade of toxicities experienced by participants will be summarised for each CTCAE term reported, by treatment arm, for the acute and late toxicities.

The assessment of PFS and OS will be based on a time to event analysis and presented using Kaplan Meier curves. Median PFS and OS will be presented with 95% CIs for each arms. There will be no formal comparison between arms, however, the unadjusted OS hazard ratio (HR) for the re-irradiation arm vs the chemotherapy arm will be presented.

Qualitative interviews

Qualitative exploration of HRQoL associated with brain tumour treatments will allow more in-depth evaluation of what matters most to patients and informal caregivers at the time of GBM recurrence; their perception of HRQoL before and during trial treatment; and how they experience both the treatment they receive and trial participation. Interviews will be recorded, transcribed, and thematically analysed [36]. A sub-sample of trial participants will be asked to partake in this interview, around 15 patients and their informal caregivers. We will utilise a maximum variation sample, to choose patients (and their informal caregivers) that differ in age, sex and previous/current treatment(s)

For peer review only

For peer review only - http://bmjopen.bmj.com/site/about/guidelines.xhtml

received. By using a varied sample, we aim to highlight important shared patterns that cut across cases and also discover unique or diverse variations from different participants [37].

Trial organisation

University of Leeds CTRU will manage all trial and data co-ordination according to the unit's well established standard operating procedures. All trial organisation will be conducted in-line with the principles of Good Clinical Practice.

Data collection and management

The BRIOChe trial data collection has been designed for remote data entry. However, all safety data will be recorded on paper case report forms and Patient Reported Outcome Measures (PROMs) on the relevant paper questionnaires, sent to and entered at CTRU. Participating centres will record and complete patient data on a trial specific database. The database has instantaneous validation checking. In addition, data management validation reports for missing and discrepant data.

All trial data is stored confidentially in a secure location at University of Leeds CTRU. Access to trial data will be restricted to CTRU staff and study team only, prior to the analysis and release of the trial results. Upon completion of the trial and publishing of results, researchers can request access to data by contacting members of the BRIOChe trial management group (TMG) or CTRU. All data will be stored and archived for a minimum of 25 years after trial completion.

For peer review only - http://bmjopen.bmj.com/site/about/guidelines.xhtml

367 **Quality assurance**

368 The radiotherapy quality assurance (QA) programme will be implemented by the National
369 Cancer Research Institute (NCRI) Radiotherapy Trials Quality Assurance (RTTQA) group to
370 ensure treatment is planned and delivered according to the trial protocol. RTTQA includes
371 both benchmarking contouring and planning cases. A summary of the RTTQA requirements
372 are provided in the BRIOChe radiotherapy outlining, planning, treatment delivery and QA
373 guidelines. All sites must complete essential documentation and the site initiation process
374 before a site can be activated. The site initiation will be an electronic process including an
375 audio-visual recording. The video recorded site initiation presentation slides can be used for
376 future training and reference at participating sites.

377

378 **Trial monitoring**

379 The BRIOChe trial will be monitored by the specific project delivery group at CTRU and the
380 multi-disciplinary TMG. An independent data monitoring and ethics committee (DMEC) will
381 be responsible for the safety and integrity of the patients and study, monitoring unblinded
382 interim data. The DMEC will be formed of two independent clinical oncologist and one
383 independent statistician, meeting at least annually, in addition to receiving 6-monthly safety
384 reports. The DMEC members will advise the Trial Steering Committee (TSC) on any trial or
385 safety concerns. The TSC have definitive control over the continuation of the trial. A DMEC
386 charter has been developed including the roles and responsibilities, and communication
387 processes.

388

389 **Patient and Public Involvement**

390 Patient and Public Involvement (PPI) has been an instrumental part of the development
391 and conduct of the BRIOChe trial. The trial design was discussed with and presented to

For peer review only

PPI representatives. The trial question was felt to be of importance and the treatments and assessment schedule were considered acceptable.

The Trial Management Group includes a key patient representative, who provides ongoing and regular advice regarding trial conduct from the patient perspective.

Imaging sub-study

An imaging sub-study is planned between two of the recruiting centres (Leeds Teaching Hospitals NHS Trust and University College London Hospitals NHS Foundation Trust), to investigate the impact of re-irradiation and chemotherapy treatments on cerebral vascular changes and to improve understanding as to how these changes might be associated with both tumour response and normal tissue damage. The aim is to recruit 16 patients to the imaging sub-study from the full BRIOChe trial. Sub-study participants who have been randomised to re-irradiation will have Arterial Spin Labelling (ASL) and T2 Dynamic Susceptibility Contrast (DSC) sequences added to the dedicated radiotherapy planning MRI. In addition, these participants will have ASL added to their routine follow up MRI scans (T2 DSC already included), to provide ‘before’ and ‘after’ re-irradiation information. Those sub-study participants randomised to chemotherapy will also have ASL and T2 DSC sequences added to their routine follow-up MRI to allow comparison between chemotherapy-treated and re-irradiated participants.

For peer review only

ETHICS AND DISSEMINATION

The trial obtained ethical approval from the Office for Research Ethics Committees Northern Ireland (ORECNI) (reference no. 20/NI/0070) and is registered in the ISRCTN registry (registration number 16052954). The trial is currently adhering to protocol version 4.0 (7/12/2022). Any protocol amendments will be submitted to MHRA and REC, all changes will be communicated with local sites.

There is no formal interim analysis. The final trial data will be analysed and reported approximately one year after the final participant is recruited. The final trial result manuscript will be written in accordance to ICMJE guidelines and submitted for publication to peer reviewed journals.

DISCUSSION

Despite the poor outcomes and high clinical need, there is a lack of active clinical trials for patients with recurrent GBM in the UK and no gold standard treatment at recurrence exists. Chemotherapy is often used in the UK, but outcomes are disappointing. Re-irradiation is recognised as an alternative treatment option and is listed as such in European and American treatment guidelines for patients with recurrent GBM, and in the current NICE guidelines for brain tumours [38-40]. Despite this, re-irradiation is less commonly utilised in the UK compared to other parts of the world. Re-irradiation offers a shorter course of treatment compared to chemotherapy and has a different toxicity profile, and so may be preferable for some patients, although the risks and benefits need further clarification. The BRIOChe trial aims to be a pragmatic, clinically relevant trial that evaluates re-irradiation as an alternative treatment option to chemotherapy for patients with recurrent GBM. While OS is the primary end point, HRQoL is a key secondary endpoint.

For peer review only

For peer review only - http://bmjopen.bmj.com/site/about/guidelines.xhtml

The trial design and treatment, specified in BRIOChe, intentionally reflects routine practice. Centres are able to use their usual lomustine-based chemotherapy regimen(s) thus reflecting the variety in practice across the UK. The frequently used and relatively well-tolerated hypofractionated schedule, 35Gy in 10 fractions, has been adopted for BRIOChe re-irradiation regimen (Klobukowski *et al.* 2018; Fogh *et al.* 2010; Hudes *et al.* 1999; Tsien *et al.* 2019).

No organ at risk (OAR) constraints have been formally validated for GBM re-irradiation. Most GBM recurrences occur within the previous high dose region and so it is often necessary to assume that a degree of OAR recovery has occurred following the original course of radiotherapy if a meaningful dose is to be delivered to the re-irradiation target. As such, BRIOChe provides ‘radiobiological’ optimal constraints, allowing for 25% recovery from the original radiotherapy, based on limited evidence regarding post-irradiation recovery in neural structures [41-43]. In addition, where optimal constraints limit re-irradiation delivery, the more lenient mandatory constraints have been based on the RTOG-1205 trial [44].

The minimal acceptable interval between completion of original radiotherapy and commencement of re-irradiation is also unknown and this interval varies amongst existing studies. We have adopted a minimum interval of 6 months from completion of first radiotherapy in BRIOChe, which is in-keeping with current practices in other countries [47] and international trials using the same re-irradiation schedule [31, 32]. An interval of at least 6 months also allows some time for OAR repair, should repair occur, and reduces the risk of mistaking pseudo-progression for true progression, which typically occurs within 3 months of radiotherapy [48].

For peer review only

While the patient numbers involved will not permit a direct comparison of survival outcomes in each arm, the aim of this study is not to establish re-irradiation as superior to chemotherapy. Instead, we hope to demonstrate that re-irradiation is an effective and acceptable treatment option for patients with recurrent GBM, including from the patient perspective. This in turn will allow the development of future novel approaches, including potential treatment intensification strategies using re-irradiation in combination with novel agents such as immunotherapies and radiosensitisers, to be explored in large-scale platform studies.

The BRIOChe study will provide valuable data for patients with recurrent GBM treated with re-irradiation and nitrosourea-based chemotherapy, forming a first step in improving treatment for this poor prognosis patient group.

For peer review only

AUTHOR CONTRIBUTIONS

Contributors to the conception and design: LM, FB, SCS

Development of the protocol, trial documents and patient information sheet: EH, LM, SB, SN, FB, CP, SC, JL.

Writing manuscript: EH, LM.

Reviewed manuscript: all authors

All authors read and approved the final manuscript.

FUNDING, SPONSOR AND COORDINATION

BRIOChe is supported by The Jon Moulton Charity Trust. The study is coordinated by University of Leeds CTRU and sponsored by University of Leeds (GA18/118305). For enquires, trial contact *email: BRIOCHE@leeds.ac.uk.*

The imaging sub-study is supported by Cancer Research UK funding for the Leeds Radiotherapy Research Centre of Excellence (RadNet; C19942/A28832).

The sponsor and funders have had no influence on the BRIOChe trial study design, collection, management, analysis, and interpretation of data; and decision for publication. The sponsor and funder will review all manuscripts prior to publication.

COMPETING INTERESTS

FS reports grants from Cancer Research UK, during the conduct of the study. DSM reports grants from Jon Moulton Charitable Foundation, grants from Yorkshire Cancer Research, during the conduct of the study. LM reports grants from Jon Moulton Charity Trust, grants from Cancer Research UK, during the conduct of the study; grants from Yorkshire Cancer Research, outside the submitted work. SB reports grants from Jon Moulton Charitable

For peer review only

Foundation, grants from Yorkshire Cancer Research, during the conduct of the study. SS reports advisory board activity for Bayer, CeCaVa Laboratory Research project support, Apollomics.

EXCLUSIVE LICENCE

I, the Submitting Author has the right to grant and does grant on behalf of all authors of the Work (as defined in the below author licence), an exclusive licence and/or a non-exclusive licence for contributions from authors who are: i) UK Crown employees; ii) where BMJ has agreed a CC-BY licence shall apply, and/or iii) in accordance with the terms applicable for US Federal Government officers or employees acting as part of their official duties; on a worldwide, perpetual, irrevocable, royalty-free basis to BMJ Publishing Group Ltd (“BMJ”) its licensees and where the relevant Journal is co-owned by BMJ to the co-owners of the Journal, to publish the Work in BMJ Open and any other BMJ products and to exploit all rights, as set out in our licence.

The Submitting Author accepts and understands that any supply made under these terms is made by BMJ to the Submitting Author unless you are acting as an employee on behalf of your employer or a postgraduate student of an affiliated institution which is paying any applicable article publishing charge (“APC”) for Open Access articles. Where the Submitting Author wishes to make the Work available on an Open Access basis (and intends to pay the relevant APC), the terms of reuse of such Open Access shall be governed by a Creative Commons licence – details of these licences and which Creative Commons licence will apply to this Work are set out in our licence referred to above.

For peer review only

525 **FIGURE LEGEND:** Figure 1 - BRIOChe Trial schema

526

For peer review only

For peer review only - http://bmjopen.bmj.com/site/about/guidelines.xhtml

REFERENCES

1. Stupp, R., et al., *Radiotherapy plus concomitant and adjuvant temozolomide for glioblastoma.* New England journal of medicine, 2005. **352**(10): p. 987-996.
2. Gately, L., et al., *Life beyond a diagnosis of glioblastoma: a systematic review of the literature.* Journal of Cancer Survivorship, 2017. **11**(4): p. 447-452.
3. Mummudi, N. and R. Jalali, *Palliative care and quality of life in neuro-oncology.* F1000prime reports, 2014. **6**.
4. Arvold, N.D., et al., *Hospitalization burden and survival among older glioblastoma patients.* Neuro-oncology, 2014. **16**(11): p. 1530-1540.
5. Mukand, J.A., et al., *Incidence of neurologic deficits and rehabilitation of patients with brain tumors.* American journal of physical medicine & rehabilitation, 2001. **80**(5): p. 346-350.
6. Boele, F.W., et al., *Symptom management and quality of life in glioma patients.* CNS oncology, 2014. **3**(1): p. 37-47.
7. Sterckx, W., et al., *The impact of a high-grade glioma on everyday life: a systematic review from the patient's and caregiver's perspective.* European Journal of Oncology Nursing, 2013. **17**(1): p. 107-117.
8. Weller, M., et al., *Standards of care for treatment of recurrent glioblastoma—are we there yet?* Neuro-oncology, 2013. **15**(1): p. 4-27.
9. van Linde, M.E., et al., *Treatment outcome of patients with recurrent glioblastoma multiforme: a retrospective multicenter analysis.* Journal of neuro-oncology, 2017. **135**(1): p. 183-192.
10. Das, P., S. McCaffrey, and D. Conkey, *Re-evaluating the efficacy and toxicity of procarbazine, lomustine and vincrsitine (PCV) in relapsed glioblastoma in the concurrent/ adjuvant temozolamide era.* Journal of Cancer Prevention and Current Research, 2016. **6**(3): p. 1-6.
11. Taal, W., et al., *Single-agent bevacizumab or lomustine versus a combination of bevacizumab plus lomustine in patients with recurrent glioblastoma (BELOB trial): a randomised controlled phase 2 trial.* The lancet oncology, 2014. **15**(9): p. 943-953.
12. Carvalho, B.F., et al., *Second-line chemotherapy in recurrent glioblastoma: a 2-cohort study.* Oncology research and treatment, 2015. **38**(7-8): p. 348-354.
13. Brada, M., et al., *Temozolomide versus procarbazine, lomustine, and vincristine in recurrent high-grade glioma.* J Clin Oncol, 2010. **28**(30): p. 4601-4608.
14. Wick, W., et al., *Lomustine and bevacizumab in progressive glioblastoma.* New England Journal of Medicine, 2017. **377**(20): p. 1954-1963.
15. Rajan, B., et al., *Survival in patients with recurrent glioma as a measure of treatment efficacy: prognostic factors following nitrosourea chemotherapy.* European Journal of Cancer, 1994. **30**(12): p. 1809-1815.
16. Wick, W., et al., *Phase III study of enzastaurin compared with lomustine in the treatment of recurrent intracranial glioblastoma.* Journal of clinical oncology, 2010. **28**(7): p. 1168.
17. Lombardi, G., et al. *Updated results of REGOMA: A randomized, multicenter, controlled open-label phase II clinical trial evaluating regorafenib in relapsed glioblastoma (GBM) patients (PTS)*. in *Oral Presentation at EANO (Eurpoean Association of Neuro-Oncolgy) Annual conference*. 2018.
18. Dirven, L., et al., *Dutch Neuro-Oncology Group (LWNO). The impact of bevacizumab on health-related quality of life in patients treated for recurrent glioblastoma: results of the randomised controlled phase 2 BELOB trial.* Eur J Cancer, 2015. **51**(10): p. 1321-1330.
19. Pessina, F., et al., *Role of surgical resection in recurrent glioblastoma: prognostic factors and outcome evaluation in an observational study.* Journal of neuro-oncology, 2017. **131**(2): p. 377-384.
20. Franceschi, E., et al., *The effect of re-operation on survival in patients with recurrent glioblastoma.* Anticancer research, 2015. **35**(3): p. 1743-1748.

For peer review only - http://bmjopen.bmj.com/site/about/guidelines.xhtml

21. Gorlia, T., et al., *New prognostic factors and calculators for outcome prediction in patients with recurrent glioblastoma: a pooled analysis of EORTC Brain Tumour Group phase I and II clinical trials.* European journal of cancer, 2012. **48**(8): p. 1176-1184.
22. Kazmi, F., et al., *Re-irradiation for recurrent glioblastoma (GBM): a systematic review and meta-analysis.* Journal of neuro-oncology, 2019. **142**(1): p. 79-90.
23. Shanker, M., et al., *Re-irradiation for recurrent high-grade gliomas: a systematic review and analysis of treatment technique with respect to survival and risk of radionecrosis.* Neuro-Oncology Practice, 2019. **6**(2): p. 144-155.
24. Nieder, C., et al., *Improvement, clinical course, and quality of life after palliative radiotherapy for recurrent glioblastoma.* American journal of clinical oncology, 2008. **31**(3): p. 300-305.
25. Grant, R., et al., *OP01THE UK TOP 10 CLINICAL RESEARCH PRIORITIES IN NEURO-ONCOLOGY.* Neuro-Oncology, 2015. **17**(Suppl 8): p. viii16.
26. Sargent, D.J., V. Chan, and R.M. Goldberg, *A three-outcome design for phase II clinical trials.* Controlled clinical trials, 2001. **22**(2): p. 117-125.
27. Klobukowski, L., et al., *A retrospective review of re-irradiating patients' recurrent high-grade gliomas.* Clinical Oncology, 2018. **30**(9): p. 563-570.
28. Fogh, S.E., et al., *Hypofractionated stereotactic radiation therapy: an effective therapy for recurrent high-grade gliomas.* Journal of clinical oncology, 2010. **28**(18): p. 3048.
29. Hudes, R.S., et al., *A phase I dose escalation study of hypofractionated stereotactic radiotherapy as salvage therapy for persistent or recurrent malignant glioma.* International Journal of Radiation Oncology* Biology* Physics, 1999. **43**(2): p. 293-298.
30. Tsien, C., et al., *ACTR-32. NRG ONCOLOGY RTOG 1205: randomized phase II trial of concurrent bevacizumab and re-irradiation vs. bevacizumab alone as treatment for recurrent glioblastoma.* Neuro-Oncology, 2019. **21**(Suppl 6): p. vi20.
31. RTOG 1205. *Randomized phase II trial of concurrent bevacizumab and re-irradiation versus bevacizumab alone as treatment of recurrent glioblastoma*. 2016; Available from: https://clinicaltrials.gov/ct2/show/NCT01730950.
32. AstraZeneca, *AZD1390 Radiation Procedure Manual. Version 1.0.* 2017.
33. Abraha, I. and A. Montedori, *Modified intention to treat reporting in randomised controlled trials: systematic review.* Bmj, 2010. **340**.
34. Batchelor, T.T., et al., *Phase III randomized trial comparing the efficacy of cediranib as monotherapy, and in combination with lomustine, versus lomustine alone in patients with recurrent glioblastoma.* Journal of Clinical Oncology, 2013. **31**(26): p. 3212.
35. Boiardi, A., et al., *Efficacy of '8-drugs-in-one-day'combination in treatment of recurrent GBM patients.* Journal of neuro-oncology, 1992. **12**(2): p. 153-158.
36. Fereday, J. and E. Muir-Cochrane, *Demonstrating rigor using thematic analysis: A hybrid approach of inductive and deductive coding and theme development.* International journal of qualitative methods, 2006. **5**(1): p. 80-92.
37. Patton, M., *Pattern, theme, and content analysis*, in *Qualitative research & evaluation methods*. 2002, Sage Publications Ltd, London. p. 452-471.
38. Cabrera, A.R., et al., *Radiation therapy for glioblastoma: executive summary of an American Society for Radiation Oncology evidence-based clinical practice guideline.* Practical radiation oncology, 2016. **6**(4): p. 217-225.
39. Stupp, R., et al., *High-grade glioma: ESMO Clinical Practice Guidelines for diagnosis, treatment and follow-up.* Annals of Oncology, 2014. **25**: p. iii93-iii101.
40. NICE, N.I.f.H.a.C.E., *Brain tumours (primary) and brain metastases in adults.* NICE guideline [NG99], 2018.
41. Kirkpatrick, J.P., A.J. van der Kogel, and T.E. Schultheiss, *Radiation dose–volume effects in the spinal cord.* International Journal of Radiation Oncology* Biology* Physics, 2010. **76**(3): p. S42-S49.
42. Ang, K.K., et al., *Extent and kinetics of recovery of occult spinal cord injury.* International Journal of Radiation Oncology* Biology* Physics, 2001. **50**(4): p. 1013-1020.

For peer review only - http://bmjopen.bmj.com/site/about/guidelines.xhtml

43. Flickinger, J.C., M. Deutsch, and L.D. Lunsford, *Repeat megavoltage irradiation of pituitary and suprasellar tumors.* International Journal of Radiation Oncology* Biology* Physics, 1989. **17**(1): p. 171-175.
44. Tsien, C., et al., *Randomized phase II trial of re-irradiation and concurrent bevacizumab versus bevacizumab alone as treatment for recurrent glioblastoma (NRG Oncology/RTOG 1205): initial outcomes and RT plan quality report.* International Journal of Radiation Oncology Biology and Physics, 2019. **105**(1).
45. Combs, S.E., et al., *Generation and validation of a prognostic score to predict outcome after re-irradiation of recurrent glioma.* Acta Oncologica, 2013. **52**(1): p. 147-152.
46. Niyazi, M., et al., *Independent validation of a new reirradiation risk score (RRRS) for glioma patients predicting post-recurrence survival: a multicenter DKTK/ROG analysis.* Radiotherapy and Oncology, 2018. **127**(1): p. 121-127.
47. Post, C.C., et al., *Patterns of re-irradiation for recurrent gliomas and validation of a prognostic score.* Radiotherapy and Oncology, 2019. **130**: p. 156-163.
48. De Wit, M., et al., *Immediate post-radiotherapy changes in malignant glioma can mimic tumor progression.* Neurology, 2004. **63**(3): p. 535-537.
49. Parsons, D.W., et al., *An integrated genomic analysis of human glioblastoma multiforme.* science, 2008. **321**(5897): p. 1807-1812.

For peer review only

For peer review only - http://bmjopen.bmj.com/site/about/guidelines.xhtml

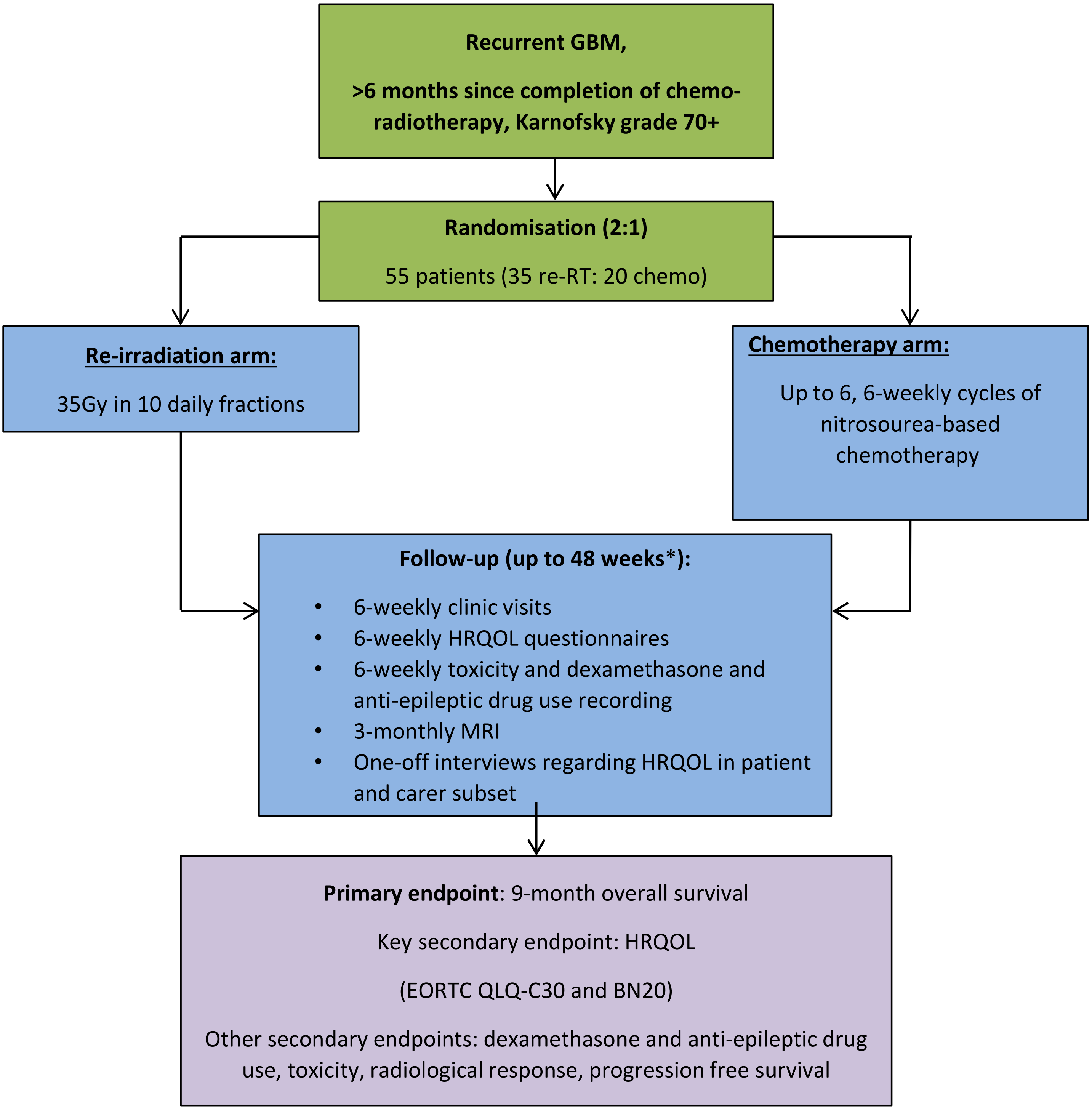

EORTC QLQ-C30 and BN20, European Organization for Research and Treatment of Cancer Quality of life questionnaire core 30 and Brain Cancer module 20; GBM, glioblastoma multiforme; HRQoL, health-related quality of life; MRI, magnetic resonance imaging;

SUPLEMENTARY FILE 1 – Full inclusion exclusion criteria

Inclusion criteria	Exclusion criteria
1. Histologically proven diagnosis of GBM with consistent molecular pathology, based on original pathology.	1. Pregnant (positive pregnancy test) or lactating
2. First recurrence of GBM, with contrast enhancing disease, following primary treatment (i.e. no previous systemic therapy or re-irradiation for recurrence).	2. Critical normal brain structures treated above usual tolerance during initial radiotherapy
3. Agreement of a Consultant Neuro-Radiologist within multi-disciplinary team meeting that imaging changes are in keeping with recurrence and not pseudo-progression.	3. Recurrence with leptomeningeal disease or only leptomeningeal disease
4. Randomisation must be performed within 21 days of the MRI that confirms recurrence.	4. Recurrence defined by non-enhancing disease only
5. ≥6 months since completion of primary radiotherapy	5. More than three enhancing lesions present on MRI or multi-focal recurrence
6. Prior history of standard dose, conventionally fractionated CNS radiotherapy	6. IDH1/2 mutant tumours on original pathology (to avoid unbalance between arms).
7. As a minimum patients will have completed at least two weeks of temozolomide, concurrent with their original radiotherapy.	7. GBM with known features of PXA, BRAF mutations or 1p19q co-deletion (on original pathology or updated pathology if available)
• Up to and including three enhancing lesions:<br>○ In cases of a single recurrent enhancing lesion:<br>▪ Predicted re-irradiation GTV<75cm^3 and<br>▪ Maximum diameter of enhancing disease must be ≤6cm.<br>○ In cases of multiple (i.e. two or three) discrete recurrent enhancing lesions:<br>▪ The total (i.e. combined) predicted re-irradiation GTV must be <50cm^3 and lesions must be clustered in a similar brain region such that PTVs are anticipated to be adjacent or overlapping and<br>▪ Maximum diameter of the combined enhancing disease, across all enhancing lesions (including any gaps between), must be ≤6cm.	8. Prior invasive malignancy (except non-melanomatous skin cancer), unless disease free for a minimum of one year.

For peer review only

8. Karnofsky Performance Status 70+	9. Severe active co-morbidity making patient unsuitable for chemotherapy or re-irradiation
9. Adequate hematologic, renal, and hepatic function	10. Prior allergic reaction to nitrosoureas
10. Patients who have had surgery for first recurrence may also be included provided there is residual enhancing disease on the immediate post-operative MRI or if enhancing disease develops on subsequent follow-up imaging, provided no prior systemic therapy or re-irradiation for recurrence has been given.	11. Any recognised genetic syndromes causing sensitivity to radiotherapy
11. Patients must have recovered from the prior effects of therapy	12. Patient unwilling/ unable to attend for follow up in the radiotherapy centre
12. Medical history and physical examination, including CNS examination, must be performed within 14 days prior to randomisation.	13. Contra-indication to MRI or gadolinium
13. Female participants of child-bearing potential must agree to be pregnancy screened.	14. Previous radiotherapy dose distribution unavailable
14. Patients must be able to swallow oral medication.	15. Previous systemic therapy or re-irradiation for recurrent GBM
15. Patients must be able to provide study-specific informed consent.	16. Spinal or infratentorial disease
16. Age 18 or over.	
17. Patients should be able to start treatment within 21 days of randomisation and must start within 28 days of randomisation.	

Coeliac disease is usually considered a contra-indication to lomustine as there is wheat within the capsule. For patients with recurrent GBM, the individual clinician may decide whether or not this is a relevant exclusion criteria.

For peer review only

Delete this line, then print on Trust/Hospital headed paper

Participant ID:	Initials:
Date of Birth:	NHS/Hospital Number:
EudraCT Number: 2019-004053-91	Principal Investigator:

BRIOChe

SAMPLE
For peer review only

Please initial each box

PARTICIPANT CONSENT FORM

1. I confirm that I have read and understood the information sheet for the above study and have had the opportunity to ask questions. ☐

2. I understand that my participation in this study is voluntary and that I am free to withdraw at any time without my medical care or legal rights being affected. I understand that even if I withdraw from the above study, the data and samples collected from me will be used in analysing the results of the study and in some cases further information about any unwanted effects of my treatment may need to be collected by the study team. ☐

3. I understand that my healthcare records may be looked at by authorised individuals from the study team, regulatory bodies or Sponsor in order to check that the study is being carried out correctly. ☐

4. I agree to allow any information or results arising from this study to be used for healthcare and/or further medical research upon the understanding that my identity will remain anonymous wherever possible. This includes the use of my radiotherapy data and imaging data for future analysis. ☐

5. I understand that if I lose capacity, further information about any unwanted effects of my treatment may need to be collected by the study team.

6. If selected, I agree to take part in a semi-structured interview on my experiences of participating in the trial and my health-related quality of life, where a member of the research team may approach me directly to schedule a suitable day and time. I agree to these interviews being audio recorded.

7. I agree to consent to my anonymised trial data to be viewed if required by 3rd parties outside the UK / EU etc.

8. I agree to a copy of this Consent Form being sent to the Clinical Trials Research Unit.

9. I agree that my GP, or any other doctor treating me, will be notified of my participation in this study.

10. I agree to take part in the study.

The following points are **OPTIONAL.**

Title: Consent for potential tissue collection and use in future research (if you do not wish to give this permission do not initial the boxes – you can still participate in the trial)

Please initial each box

	Yes	No
I give my permission for stored tumour tissue sample that was removed during my operation and was not needed for routine diagnosis and treatment to be collected and used for future translational research purposes as described in the information sheet for the above trial if this becomes relevant in the future.		
I understand how the tissue sample will be collected, that giving samples is voluntary and that I'm free to withdraw my approval for use of the samples at any time without giving a reason and without my medical care or legal rights being affected.		

SAMPLE

For peer review only

Patient:
Signature……………………………………………………………………………

Name (block capitals)…………………………………………………………………

Date……………………………………………………………………………………

Investigator:

I have explained the study to the above named patient and he/she has indicated his/her willingness to participate.

Signature……………………………………………………………………………

Name (block capitals)…………………………………………………………………

Date……………………………………………………………………………………

(If used)Translator:

Signature……………………………………………………………………………

Name (block capitals)…………………………………………………………………

Date……………………………………………………………………………………

(1 copy for patient; 1 for the CTRU; 1 held in patient notes;

SAMPLE
For peer review only

SPIRIT

STANDARD PROTOCOL ITEMS: RECOMMENDATIONS FOR INTERVENTIONAL TRIALS

SPIRIT 2013 Checklist: Recommended items to address in a clinical trial protocol and related documents*

Section/item	Item No	Description	Addressed on page number
**Administrative information**			
Title	1	Descriptive title identifying the study design, population, interventions, and, if applicable, trial acronym	1
Trial registration	2a	Trial identifier and registry name. If not yet registered, name of intended registry	4, 23
	2b	All items from the World Health Organization Trial Registration Data Set	Many
Protocol version	3	Date and version identifier	23
Funding	4	Sources and types of financial, material, and other support	4, 26
Roles and responsibilities	5a	Names, affiliations, and roles of protocol contributors	1, 26
	5b	Name and contact information for the trial sponsor	26
	5c	Role of study sponsor and funders, if any, in study design; collection, management, analysis, and interpretation of data; writing of the report; and the decision to submit the report for publication, including whether they will have ultimate authority over any of these activities	26
	5d	Composition, roles, and responsibilities of the coordinating centre, steering committee, endpoint adjudication committee, data management team, and other individuals or groups overseeing the trial, if applicable (see Item 21a for data monitoring committee)	20, 21

For peer review only

Introduction

Background and rationale	6a	Description of research question and justification for undertaking the trial, including summary of relevant studies (published and unpublished) examining benefits and harms for each intervention	6,7, 8_____
	6b	Explanation for choice of comparators	6, 7__________
Objectives	7	Specific objectives or hypotheses	11 _________
Trial design	8	Description of trial design including type of trial (e.g., parallel group, crossover, factorial, single group), allocation ratio, and framework (e.g., superiority, equivalence, non-inferiority, exploratory)	9 ___________

Methods: Participants, interventions, and outcomes

Study setting	9	Description of study settings (e.g., community clinic, academic hospital) and list of countries where data will be collected. Reference to where list of study sites can be obtained	9 ________
Eligibility criteria	10	Inclusion and exclusion criteria for participants. If applicable, eligibility criteria for study centres and individuals who will perform the interventions (e.g., surgeons, psychotherapists)	13, Table 1, Supplementary file 1
Interventions	11a	Interventions for each group with sufficient detail to allow replication, including how and when they will be administered	15__________
	11b	Criteria for discontinuing or modifying allocated interventions for a given trial participant (e.g., drug dose change in response to harms, participant request, or improving/worsening disease)	15, 16_________
	11c	Strategies to improve adherence to intervention protocols, and any procedures for monitoring adherence (e.g., drug tablet return, laboratory tests)	14,15 ________
	11d	Relevant concomitant care and interventions that are permitted or prohibited during the trial	N/A________
Outcomes	12	Primary, secondary, and other outcomes, including the specific measurement variable (e.g., systolic blood pressure), analysis metric (e.g., change from baseline, final value, time to event), method of aggregation (e.g., median, proportion), and time point for each outcome. Explanation of the clinical relevance of chosen efficacy and harm outcomes is strongly recommended	11 _____

Participant timeline	13	Time schedule of enrolment, interventions (including any run-ins and washouts), assessments, and visits for participants. A schematic diagram is highly recommended (see Figure)	Table 2, Figure 1
Sample size	14	Estimated number of participants needed to achieve study objectives and how it was determined, including clinical and statistical assumptions supporting any sample size calculations	14_______
Recruitment	15	Strategies for achieving adequate participant enrolment to reach target sample size	12______

Methods: Assignment of interventions (for controlled trials)

Allocation:

Sequence generation	16a	Method of generating the allocation sequence (e.g., computer-generated random numbers), and list of any factors for stratification. To reduce predictability of a random sequence, details of any planned restriction (eg, blocking) should be provided in a separate document that is unavailable to those who enrol participants or assign interventions	13, 14 __________
Allocation concealment mechanism	16b	Mechanism of implementing the allocation sequence (e.g., central telephone; sequentially numbered, opaque, sealed envelopes), describing any steps to conceal the sequence until interventions are assigned	13, 14 ___________
Implementation	16c	Who will generate the allocation sequence, who will enrol participants, and who will assign participants to interventions	11, 12, 13______
Blinding (masking)	17a	Who will be blinded after assignment to interventions (e.g., trial participants, care providers, outcome assessors, data analysts), and how	N/A _________
	17b	If blinded, circumstances under which unblinding is permissible, and procedure for revealing a participant's allocated intervention during the trial	N/A__________

Methods: Data collection, management, and analysis

Data collection methods	18a	Plans for assessment and collection of outcome, baseline, and other trial data, including any related processes to promote data quality (e.g., duplicate measurements, training of assessors) and a description of study instruments (e.g., questionnaires, laboratory tests) along with their reliability and validity, if known. Reference to where data collection forms can be found, if not in the protocol	10, 11, (Figure 1)

	18b	Plans to promote participant retention and complete follow-up, including list of any outcome data to be collected for participants who discontinue or deviate from intervention protocols	16__________
Data management	19	Plans for data entry, coding, security, and storage, including any related processes to promote data quality (e.g., double data entry; range checks for data values). Reference to where details of data management procedures can be found, if not in the protocol	20 _________
Statistical methods	20a	Statistical methods for analysing primary and secondary outcomes. Reference to where other details of the statistical analysis plan can be found, if not in the protocol	18,19______
	20b	Methods for any additional analyses (e.g., subgroup and adjusted analyses)	18,19_______
	20c	Definition of analysis population relating to protocol non-adherence (e.g., as randomised analysis), and any statistical methods to handle missing data (e.g., multiple imputation)	18 _________
**Methods: Monitoring**			
Data monitoring	21a	Composition of data monitoring committee (DMC); summary of its role and reporting structure; statement of whether it is independent from the sponsor and competing interests; and reference to where further details about its charter can be found, if not in the protocol. Alternatively, an explanation of why a DMC is not needed	21 __________
	21b	Description of any interim analyses and stopping guidelines, including who will have access to these interim results and make the final decision to terminate the trial	21, 23 ________
Harms	22	Plans for collecting, assessing, reporting, and managing solicited and spontaneously reported adverse events and other unintended effects of trial interventions or trial conduct	17 ____
Auditing	23	Frequency and procedures for auditing trial conduct, if any, and whether the process will be independent from investigators and the sponsor	20_________
**Ethics and dissemination**			
Research ethics approval	24	Plans for seeking research ethics committee/institutional review board (REC/IRB) approval	23 ___________

Protocol amendments	25	Plans for communicating important protocol modifications (e.g., changes to eligibility criteria, outcomes, analyses) to relevant parties (e.g., investigators, REC/IRBs, trial participants, trial registries, journals, regulators)	23____________
Consent or assent	26a	Who will obtain informed consent or assent from potential trial participants or authorised surrogates, and how (see Item 32)	11 ___________
	26b	Additional consent provisions for collection and use of participant data and biological specimens in ancillary studies, if applicable	N/A__________
Confidentiality	27	How personal information about potential and enrolled participants will be collected, shared, and maintained in order to protect confidentiality before, during, and after the trial	20 ________
Declaration of interests	28	Financial and other competing interests for principal investigators for the overall trial and each study site	26 ___________
Access to data	29	Statement of who will have access to the final trial dataset, and disclosure of contractual agreements that limit such access for investigators	20 ________
Ancillary and post-trial care	30	Provisions, if any, for ancillary and post-trial care, and for compensation to those who suffer harm from trial participation	N/A ________
Dissemination policy	31a	Plans for investigators and sponsor to communicate trial results to participants, healthcare professionals, the public, and other relevant groups (e.g., via publication, reporting in results databases, or other data sharing arrangements), including any publication restrictions	23 _____
	31b	Authorship eligibility guidelines and any intended use of professional writers	23 __________
	31c	Plans, if any, for granting public access to the full protocol, participant-level dataset, and statistical code	20_________
**Appendices**			
Informed consent materials	32	Model consent form and other related documentation given to participants and authorised surrogates	Supplementary fie 2 _________
Biological specimens	33	Plans for collection, laboratory evaluation, and storage of biological specimens for genetic or molecular analysis in the current trial and for future use in ancillary studies, if applicable	_N/A_________

*It is strongly recommended that this checklist be read in conjunction with the SPIRIT 2013 Explanation & Elaboration for important clarification on the items. Amendments to the protocol should be tracked and dated. The SPIRIT checklist is copyrighted by the SPIRIT Group under the Creative Commons "Attribution-NonCommercial-NoDerivs 3.0 Unported" license.

For peer review only

BMJ Open

Brain Re-Irradiation Or Chemotherapy: a phase II randomised trial of re-irradiation and chemotherapy in patients with recurrent glioblastoma (BRIOChe): Protocol for a multi-centre open label randomised trial

Journal:	*BMJ Open*
Manuscript ID	bmjopen-2023-078926.R1
Article Type:	Protocol
Date Submitted by the Author:	14-Nov-2023
Complete List of Authors:	Hudson, Eleanor; University of Leeds, Leeds Institute of Clinical Trials Research<br>Noutch, Samantha; University of Leeds, Leeds Institute of Clinical Trials Research<br>Webster, Joanne; University of Leeds, Leeds Institute of Clinical Trials Research<br>Brown, Sarah; University of Leeds, Clinical Trials Research Unit<br>Boele, Florien; University of Leeds, Leeds Institute of Medical Research at St James's; University of Leeds, Leeds Institute of Health Sciences<br>Al-Salihi, Omar; Guy's and St Thomas' Hospitals NHS Trust<br>Baines, Helen; Mount Vernon Cancer Centre, National Radiotherapy Trials QA (RTTQA) Group<br>Bulbeck, Helen; brainstrust,<br>Currie, Stuart; Leeds General Infirmary, Department of Radiology<br>Fernandez, Sharon; University of Leeds, Leeds Institute of Medical Research at St James's<br>Hughes, Jane; University of Leeds, Leeds Institute of Medical Research at St James's<br>Lilley, John; Leeds Institute of Cancer and Pathology, Department of Medical Physics<br>Smith, Alexandra; University of Leeds, Leeds Institute of Clinical Trials Research<br>Parbutt, Catherine; Leeds Teaching Hospitals NHS Trust, Pharmacy<br>Slevin, Finbar; Leeds Institute of Cancer and Pathology, Department of Clinical Oncology; University of Leeds, Leeds Institute of Medical Research at St James's<br>Short, Susan; Leeds Institute of Cancer and Pathology, Department of Clinical Oncology; University of Leeds, Leeds Institute of Medical Research at St James's<br>Sebag-Montefiore, David ; University of Leeds Faculty of Medicine and Health, Institute of Cancer and Pathology<br>Murray, Louise; Leeds Institute of Cancer and Pathology, Department of Clinical Oncology; University of Leeds, Leeds Institute of Medical Research at St James's
**Primary Subject Heading**:	Oncology

Secondary Subject Heading:	Oncology
Keywords:	Radiation oncology < RADIOTHERAPY, RADIOTHERAPY, ONCOLOGY, Neurological oncology < NEUROLOGY

SCHOLARONE™
Manuscripts

Brain Re-Irradiation Or Chemotherapy: a phase II randomised trial of re-irradiation and chemotherapy in patients with recurrent glioblastoma (BRIOChe): Protocol for a multi-centre open label randomised trial

Authors:

Eleanor Hudson[1], Samantha Noutch[1], Joanne Webster[1], Sarah R. Brown[1], Florien W. Boele[2,3], Omar Al-Salihi[4], Helen Baines[5], Helen Bulbeck[6], Stuart Currie[7], Sharon Fernandez[2] Jane Hughes[2], John Lilley[8], Alexandra Smith[1], Catherine Parbutt[9], Finbar Slevin[2,10], Susan C. Short[2,10], David Sebag-Montefiore[2], Louise Murray[2,10]

Author affiliation:

[1]Clinical Trials Research Unit, Leeds Institute of Clinical Trials Research, University of Leeds, Leeds, United Kingdom

[2]Leeds Institute of Medical Research at St James's, University of Leeds, Leeds, United Kingdom

[3]Leeds Institute of Health Sciences, Faculty of Medicine and Health, University of Leeds, Leeds, UK

[4]Guy's and St Thomas Hospital, London, United Kingdom

[5]National Radiotherapy Trials QA (RTTQA) Group, United Kingdom

[6]Patient Advocate, United Kingdom

[7]Department of Radiology, Leeds General Infirmary, Leeds, United Kingdom

[8]Department of Medical Physics, Leeds Cancer Centre, Leeds, United Kingdom

[9]Pharmacy Department, St James's University Hospital, Leeds, United Kingdom

[10]Department of Clinical Oncology, Leeds Cancer Centre, Leeds, United Kingdom

For peer review only

Corresponding author: Dr. Louise Murray
L.J.Murray@leeds.ac.uk
Leeds Institute of Medical Research at St James's,
University of Leeds,
St James's University Hospital,
Harehills
Leeds,
LS9 7LP
+44 113 206 7854

Word count (excluding title page, abstract, references, figures and tables):

4180-128-234-115 = 3,703

For peer review only

ABSTRACT (300 word count)

Introduction

Glioblastoma (GBM) is the most common adult primary malignant brain tumour. The condition is incurable and, despite aggressive treatment at first presentation, almost all tumours recur after a median of 7 months. The aim of treatment at recurrence is to prolong survival and maintain health-related quality of life (HRQoL). Chemotherapy is typically employed for recurrent GBM, often using nitrosourea-based regimens. However, efficacy is limited, with reported median survivals between 5 and 9 months. Although less commonly used in the United Kingdom (UK), there is growing evidence that re-irradiation may produce survival outcomes at least similar to nitrosourea-based chemotherapy. However, there remains uncertainty as to the optimum approach and there is a paucity of available data, especially with regards to HRQoL. BRIOChe aims to assess re-irradiation, as an acceptable treatment option for recurrent IDH-wild-type GBM.

Methods and analysis

BRIOChe is a phase II, multi-centre, open-label, randomised trial in patients with recurrent GBM. The trial uses a Sargent's three-outcome design and will recruit approximately 55 participants from 10-15 UK radiotherapy sites, allocated (2:1) to receive re-irradiation (35 Gy in 10 daily fractions) or nitrosourea-based chemotherapy (up to six, 6-weekly cycles). The primary endpoint is overall survival rate for re-irradiation patients at 9 months. There will be no formal statistical comparison between treatment arms for the decision-making primary analysis. The chemotherapy arm will be used for calibration purposes, to collect concurrent data to aid interpretation of results. Secondary outcomes include HRQoL, dexamethasone

For peer review only

For peer review only - http://bmjopen.bmj.com/site/about/guidelines.xhtml

requirement, anti-epileptic drug requirement, radiological response, treatment compliance, acute and late toxicities, progression free survival.

Ethics and dissemination

BRIOChe obtained ethical approval from Office for Research Ethics Committees Northern Ireland (reference no. 20/NI/0070). Final trial results will be published in peer reviewed journals and adhere to the to ICMJE guidelines.

Registration details: BRIOChe (ISRCTN: 16052954; registered 25/02/2021)

For peer review only

For peer review only - http://bmjopen.bmj.com/site/about/guidelines.xhtml

STRENGTHS AND WEAKNESSES (up to 5)

- BRIOChe is multi-centre randomised trial exploring re-irradiation and chemotherapy for recurrent GBM.
- The trial will collect much needed survival and health related quality of life (HRQoL) data for patients with recurrent GBM treated with re-irradiation.
- BRIOChe focuses on the underserved population of recurrent GBM patients, for whom there is a significant lack of treatment options and UK-based trials.
- Although there is no formal comparison between treatment arms for the primary endpoint of overall survival, the trial has a parallel concurrent control arm for benchmarking and to aid interpretation.

For peer review only

INTRODUCTION

Glioblastoma

Glioblastoma (GBM) is the most common adult primary malignant brain tumour, with an incidence of 5/100,000 per year in England. Prognosis is poor, with an average survival of 12 to 15 months [1].

GBM is incurable, with treatment from the point of diagnosis aiming to improve survival, delay disease progression and manage symptoms/preserve HRQoL. Optimal treatment at first presentation is aggressive, including debulking surgery (if feasible), chemo-radiotherapy and adjuvant chemotherapy. However, almost all patients will relapse within the brain, with a median progression free survival (PFS) of approximately 7 months [1].

The burden placed on patients, their informal caregivers and the healthcare system as a result of neurological disability and the care requirements for patients with brain tumours is significant [2-4]. GBM is often accompanied by debilitating physical and neurological symptoms, such as paresis, sensory loss, seizures, fatigue, mood issues, and cognitive deficits [5-7]. This symptom burden can affect patients' ability to function independently and negatively impact on health-related quality of life (HRQoL) for both patients and their informal caregivers.

Recurrent glioblastoma

The optimal treatment for recurrent GBM is undefined and treatment decisions are made on a case-by-case basis, based on the site, volume and time of recurrence in relation to the original diagnosis, with consideration for patient age, co-morbidities and performance status.

In the UK, chemotherapy is commonly employed in the setting of recurrent GBM, typically using nitrosourea-based regimens. These DNA alkylating agents are highly lipophilic, and are

For peer review only

therefore able to penetrate the blood brain barrier, making them suitable for the treatment of brain tumours. Nitrosourea-combination regimens have not proven superior to single agent nitrosoureas, resulting in variations in practice as to which regimens are employed [8]. Efficacy is limited, with reported median overall survivals (OS) of between 5 and 9 months [9-17]. Side effects may include fatigue, nausea, vomiting, anorexia, constipation, paraesthesia, rash and myelosuppression. There is currently a relative lack of HRQoL data for patients receiving nitrosourea-based chemotherapy for recurrent GBM [13, 16, 18], making it difficult to determine whether the benefits of chemotherapy outweigh its burdens.

Surgery is another treatment option at point of recurrence; however, is usually reserved for a minority of excellent performance patients with well-localised disease, although high-level evidence to support its role is lacking [9, 19-21].

Re-irradiation for recurrent glioblastoma

Re-irradiation is a further treatment option for recurrent GBM, although at present it is not commonly used in the UK. A meta-analysis of re-irradiation for recurrent GBM [22] included 50 studies of re-irradiation, of which 35 employed external beam radiotherapy (EBRT; the remainder used brachytherapy). Of the patients who received EBRT, 6-month and 12-month overall survival (OS) was 70% and 34% respectively [22]. Progression free survival (PFS) was 40% and 16% at 6 and 12-months, respectively. Grade 3+ toxicity was reported in 7% of patients. Overall, only 13 of the studies were of prospective design and only 9 were considered by the authors to be of good quality. Similarly, a systematic review of re-irradiation in GBM presented comparable outcomes based on a pooled, population-weighted analysis, where mean adjusted OS following hypofractionated EBRT was 10.1 months (95% confidence interval (CI) 9.7-10.5 months) and the rate of radionecrosis was 7.1% (95% CI: 6.6-7.7%) [23]. Despite the promising outcomes reported after re-irradiation, the poor quality of existing evidence highlights the urgent need for a high quality, prospective, randomised assessment

For peer review only

of re-irradiation, including HRQoL assessment [23, 24]. More recently, the phase II RTOG1205 trial, which randomised patients with recurrent GBM between bevacizumab (not routinely available in the UK) and bevacizumab plus re-irradiation 35Gy in 10 fractions, demonstrated a median overall survival of 10.1 months in the combination arm and improved progression free survival compared to bevacizumab alone [25]. Treatment was also well tolerated, with 5% grade 3+ acute toxicities and no delayed high grade adverse events reported.

Rationale for study

While the limited existing evidence suggests that re-irradiation has a potential positive impact on overall survival and HRQoL, to date, no trial has randomised patients with recurrent GBM between re-irradiation and chemotherapy. Addressing this research gap is a top priority for patients, informal caregivers, and healthcare professionals [26].

The BRIOChe phase II prospective trial will randomise patients with recurrent GBM to re-irradiation (35 Gy in 10 fractions, a commonly adopted re-irradiation regimen [27-32]), or nitrosourea-based chemotherapy. The aims will be to explore the efficacy and HRQoL impact of re-irradiation. This study will collect concurrent data for patients receiving chemotherapy for validation (‘benchmarking’) of outcomes. The study is not designed to statistically compare the two arms for the primary outcome of survival, which would require a larger number of patients. By establishing the feasibility of randomisation and providing prospective controlled data on survival and HRQoL impacts associated with re-irradiation, the BRIOChe trial aims to demonstrate re-irradiation as an acceptable alternative treatment option for patients with recurrent GBM, guiding the design of future larger scale randomised trials.

For peer review only

METHODS AND ANALYSIS

Design and aim

BRIOChe is a phase II, multi-centre, open-label, randomised trial in patients with recurrent GBM. The aim of the study is to assess re-irradiation as an alternative treatment to chemotherapy in this group of patients. BRIOChe uses a Sargent's three-outcome, single-stage, single-arm design, with the inclusion of a chemotherapy calibration arm for concurrent data for benchmarking. Following an amendment due to slow recruitment, the trial will now recruit approximately 55 participants from 10-15 UK radiotherapy sites. The original sample size was 70 based on a 1:1 allocation ratio, to receive either re-irradiation or nitrosourea-based chemotherapy (lomustine +/- procarbazine +/- vincristine). The allocation ratio was adapted during recruitment to 2:1, further details are presented in the sample size section.

The trial schema is presented in Figure 1.

For peer review only

Trial objectives

The primary objective of BRIOChe is to assess the proportion of participants alive at 9 months post start of treatment in the re-irradiation arm.

The key secondary objective of BRIOChe is to evaluate the effect of re-irradiation on HRQoL. Questionnaires to be completed by participants include the European Organization for Research and Treatment of Cancer (EORTC) Quality of life questionnaire core 30 (QLQ-C30) and Brain Cancer module (BN20). HRQoL will also be explored in greater detail through one-off semi-structured qualitative interviews in a subsample of patients and their informal caregivers.

Additional secondary objectives include evaluating:

- Dexamethasone requirement
- Anti-epileptic drug requirement
- Radiological response in accordance with Response Assessment in Neuro-Oncology (RANO) criteria
- Treatment compliance
- Acute (measured from randomisation to 12 weeks post end of treatment of treatment) and late toxicities (after 12 weeks post end of treatment) as per Common Terminology Criteria for Adverse Events (CTCAE V.5.0.); radionecrosis is a late toxicity of particular interest
- PFS
- OS

Study population

All inclusion/exclusion criteria must be met and written informed consent obtained prior to recruitment (Key criteria summarised in Table 1); full criteria (Supplementary File 1) and a sample patient consent form (Supplementary File 2) are included as Supplementary Material.

For peer review only

The formal assessment of eligibility and informed consent may only be obtained by the Principal Investigator (PI) or an appropriate medically qualified doctor. Patients will be screened from clinic lists.

For peer review only

For peer review only - http://bmjopen.bmj.com/site/about/guidelines.xhtml

Table 1– Summary of key inclusion and exclusion criteria

Inclusion criteria	Exclusion criteria
Histologically proven diagnosis of GBM, based on original pathology.	Pregnant (positive pregnancy test) or lactating
First recurrence of GBM, with contrast enhancing disease, following primary treatment	Critical normal brain structures treated above usual tolerance during initial radiotherapy
Agreement of a Consultant Neuro-Radiologist that imaging changes are in keeping with recurrence and not pseudo-progression.	Recurrence with leptomeningeal disease or only leptomeningeal disease
≥6 months since completion of primary radiotherapy	More than three enhancing lesions present on MRI or multi-focal recurrence
Prior history of standard dose, conventionally fractionated CNS radiotherapy	IDH1/2 mutant tumours on original pathology
As a minimum, patients will have completed at least two weeks of temozolomide, concurrent with their original radiotherapy.	GBM with known features of PXA, BRAF mutations or 1p19q co-deletion (on original pathology or updated pathology if available)
Up to and including three enhancing lesions (size and volume criteria included in Supplementary Material)	Prior invasive malignancy (except non-melanomatous skin cancer), unless disease free for a minimum of one year.
Karnofsky Performance Status 70+	Severe active co-morbidity making patient unsuitable for chemotherapy or re-irradiation
Adequate hematologic, renal, and hepatic function	Prior allergic reaction to nitrosoureas
No contra-indication to lomustine	Any recognised genetic syndromes causing sensitivity to radiotherapy
Patients must be able to provide study-specific informed consent.	Contra-indication to MRI or gadolinium
Age 18 or over.	Previous radiotherapy dose distribution unavailable
	Previous systemic therapy or re-irradiation for recurrent GBM

Randomisation and recruitment

Randomisation will be performed centrally using University of Leeds Clinical Trials Research Unit (CTRU) automated 24-hour randomisation system. A computer-generated minimisation program incorporating a random element will be used to ensure the treatment groups are well balanced for the following factors:

- Age (<50 or ≥50 years)

For peer review only - http://bmjopen.bmj.com/site/about/guidelines.xhtml

- Time to randomisation from completion of previous radiotherapy (≤12 months or >12 months)
- MGMT promoter methylation status (Methylated, Unmethylated or Unknown)
- Randomising site

The intended recruitment period is 2-years (BRIOChe opened to recruitment on 22/06/2021).

Sample size

Thirty-three patients are required in the re-irradiation arm, 35 allowing for 5% drop out. With a 1:1 randomisation allocation ratio to include a calibration arm, 70 patients are required in total. As a result of slow recruitment, the randomisation allocation ratio was adapted in a protocol amendment to 2:1 (re-irradiation: chemotherapy), to allow a higher proportion of recruited patients into the intervention arm. The subsequent sample size was estimated to be 55 patients in total, with 35 in the re-irradiation arm and approximately 20 in chemotherapy arm.

This study is powered to demonstrate that the treatment strategy of re-irradiation demonstrates sufficient efficacy to warrant further large-scale evaluation, based on 9-month survival rates.

A Sargent's three-outcome, phase II, single-stage, single-arm design will be used to determine whether re-irradiation demonstrates sufficient efficacy to warrant further evaluation [33]. The trial is designed to test the null hypothesis (H0) that the proportion of participants alive at 9 months is <25% where re-irradiation would not be deemed worthy of further investigation, against an alternative hypothesis H1 of >45% where re-irradiation would be deemed worthy of further investigation. If the proportion of participants alive at 9 months is ≥25% and ≤45%, this is an inconclusive region where neither the null or alternative hypothesis would be rejected and the decision regarding further investigation would be based on other factors. The following operating characteristics are used: type I

For peer review only

For peer review only - http://bmjopen.bmj.com/site/about/guidelines.xhtml

241 error=0.05, type II error=0.06, prob (correctly rejecting the H1) = 0.82 (eta), prob (correctly
242 rejecting the H0) =0.79 (pi).

243

244 **Treatment regimen**

245 Re-irradiation arm: The radiotherapy prescription dose will be 35 Gy in 10 fractions, a
246 commonly employed schedule [27-32], delivered daily Monday-Friday over two weeks.
247 Treatment will be delivered with Intensity Modulated Radiotherapy (IMRT)/ Volumetric
248 Modulated Arc Therapy (VMAT) or tomotherapy. Optimally, normal tissue constraints will be
249 based on a 'dose remaining' approach, whereby an original organ at risk (OAR) dose
250 (allowing for 25% repair) is subtracted from a cumulative tolerance to give a 'dose remaining'
251 for re-irradiation. To save clinicians time and reduce errors in performing such calculations,
252 the radiotherapy guidelines contain tables of 'OAR doses remaining' for ease of reference.
253 Where these optimal constraints cannot be achieved, mandatory constraints are those used
254 in the RTOG1205 trial, which also delivered a dose of 35 Gy in 10 fractions [31]. Treatment
255 compliance data to radiotherapy, including fractions, doses, interruptions and reasons will be
256 collected weekly during treatment.

257 Chemotherapy arm: As per usual UK practice in the setting of relapsed GBM where the
258 interval to recurrence is insufficiently long to use re-challenge temozolomide and/or when
259 patients have O^6-methylguanine-DNA-methyltransferase (MGMT) unmethylated tumours,
260 chemotherapy will be lomustine-based. These drugs have been used in this setting for many
261 years. A variety of lomustine-based regimens are used across the UK including single agent
262 lomustine, PCV (procarbazine, lomustine and vincristine), PC (procarbazine and lomustine)
263 or CV (lomustine and vincristine). Lomustine-based regimens will be delivered as per each
264 centre's standard practice. Doses will be recorded at each cycle and (based on a survey of
265 participating centres regarding their usual practice) it is recommend that these will consist of:

266 - Lomustine 100-130mg/m^2, day 1, PO, on a 42 day cycle

For peer review only - http://bmjopen.bmj.com/site/about/guidelines.xhtml

+/-

- Procarbazine 50-100mg/m^2, once daily on days 1-10 or days 2-11, PO, on a 42 day cycle

+/-

- Vincristine 1.4-1.5mg/m^2, with dose capping as per the institution’s usual practice, IV day 1, on a 42 day cycle.

(Dose capping is as per the institution’s usual practice).

Treatment compliance data to chemotherapy will be collected from patient diaries. Dose modifications and reasons for changes will also be collected. Suggested dose modifications to chemotherapy based on haematological, renal and hepatic functioning are provided in the study protocol.

Upon further disease progression, patients may receive the alternative treatment according to local policy.

Trial assessments and follow-up

Full eligibility criteria (Table 1, Supplementary Material), will be assessed and confirmed prior to randomisation. Further assessments will be conducted at multiple timepoints. An overview of data collection can be seen in Table 2. Data will be collected from all participants, until progression or death. Where available, date of death will be collected for all participants. A final data sweep prior to the final analysis will collect information regarding death and any additional anti-cancer treatment received beyond progression.

Follow-up imaging with MRI will include T2, FLAIR (fluid attenuated inversion recovery), DWI (diffusion weighted imaging), T1 pre-gadolinium and T1 post-gadolinium sequences. In addition, patients randomised to re-irradiation will also undergo T2* Dynamic Susceptibility Contrast (DSC) perfusion as part of follow-up imaging to assist in determination of the presence of radionecrosis.

For peer review only

For peer review only - http://bmjopen.bmj.com/site/about/guidelines.xhtml

Table 2 – Trial assessment schedules

	Early assessments				Follow-up assessment (measured post start of treatment)									
	Eligibility	Baseline	Pre-treat RT	Pre-treat chemo	0w	1w	6w	12w	18w	24w	30w	36w	42w	48w
Informed consent	X													
Clinical assessment	X				X	X***	X	X	X	X	X	X	X	X
Performance status (Karnofsky)	X				X	X***	X	X	X	X	X	X	X	X
Bloods*	X			X	X	X	X	X	X	X	X			
Pregnancy screening**	X		X		X	X	X	X	X	X	X			
Re-irradiation					X	X								
Chemotherapy					X		X	X	X	X	X			
MRI (RANO criteria)	X						X			X				
Toxicity (CTCAE)		X			X	X***	X	X	X	X	X	X	X	X
HRQoL		X					X	X	X	X	X	X	X	X

* Bloods only for Eligibility for re-irradiation arm, with each cycle in chemotherapy arm

**only at pretreatment for re-irradiation arm, with every cycle in chemotherapy arm

*** re-irradiation arm patients only

Adverse Events (AEs), Serious Adverse Events (SAEs), Adverse Reaction (ARs), Serious Adverse Reactions (SARs) and Suspected Unexpected Serious Adverse Reactions (SUSARs) will all be collected from randomisation until the last participant follow-up or disease progression. Beyond this period only, SARs and SUSARs related to the trial treatment (and not GBM progression) will be reported if the investigator becomes aware of them until the end of trial notification. All SAEs/SARs and SUSARs related to investigation medicinal products (IMPs) will be reported to the Medicines and Healthcare products Regulatory Agency (MHRA) and sponsor. All radiotherapy related SAEs/SARs and SUSARs will be reported to the Research Ethics Committee and sponsor.

For peer review only

For peer review only - http://bmjopen.bmj.com/site/about/guidelines.xhtml

Statistical analysis

The statistical analysis will be conducted by University of Leeds CTRU. A full statistical analysis plan has been developed and finalised.

The trial analysis will be conducted on a Modified Intention to Treat (MITT) population [34]. MITT is defined as all participants randomised that have received at least one dose of trial treatment, analysed according to the treatment arm they were randomised to (regardless of ineligibility, non-compliance or withdrawal).

Overall survival rates at 9 months, i.e. the number and proportion of participants alive at 9 months post-start of treatment, will be calculated for the re-irradiation arm with 90% (corresponding to a 1-sided 5% significance level used in the design) and 95% confidence intervals (CIs).

Based on the 3-outcome design, the cut-off values and conclusions for the statistical test for the primary analysis of efficacy are defined as follows:

Green: If ≥13/33 patients are alive, re-irradiation demonstrates sufficient efficacy to warrant larger-scale evaluation. This is based on a 9-month OS >45% (i.e. competitive with the 'best' outcomes from prospective studies using nitrosourea-based chemotherapy [11, 14, 35].

Amber: If 11-12/33 patients are alive the decision to take re-irradiation forward will be uncertain and will be based on HRQoL and toxicity at 3 months, (this is based on 9-month OS 25%-45%; i.e. similar to outcomes from other prospective nitrosourea-based trials [13, 16, 17, 36].

Red: If ≤10/33 patients are alive re-irradiation will not be considered worthy of further investigation, based on 9-month OS <25%.

For peer review only - http://bmjopen.bmj.com/site/about/guidelines.xhtml

332 The chemotherapy arm is included for calibration and will be used for validation of outcomes
333 in the re-irradiation group with respect to previous series, and validation of the assumptions
334 used to inform the statistical cut-offs.

335 Summary statistics will be calculated for all domains of the EORTC QLQ-C30 and QLQ-
336 BN20 and the overall summary score calculated, presented overall and by treatment arm for
337 each of the follow-up time points. The difference in scores between treatment arms will be
338 presented with corresponding 90% and 95% confidence intervals.

339 Summary statistics will be presented for dexamethasone requirement, anti-epileptic drug
340 requirement, treatment compliance, and response assessment; including the proportion of
341 participants within each clinical response status. The maximum CTCAE grade of toxicities
342 experienced by participants will be summarised for each CTCAE term reported, by treatment
343 arm, for the acute and late toxicities.

344 The assessment of PFS and OS will be based on a time to event analysis and presented
345 using Kaplan Meier curves. Median PFS and OS will be presented with 95% CIs for each
346 arms. There will be no formal comparison between arms, however, the unadjusted OS
347 hazard ratio (HR) for the re-irradiation arm vs the chemotherapy arm will be presented.

348

349 **Qualitative interviews**

350 Qualitative exploration of HRQoL associated with brain tumour treatments will allow
351 more in-depth evaluation of what matters most to patients and informal caregivers at the
352 time of GBM recurrence; their perception of HRQoL before and during trial treatment;
353 and how they experience both the treatment they receive and trial participation.
354 Interviews will be recorded, transcribed, and thematically analysed [37]. A sub-sample of
355 trial participants will be asked to partake in this interview, around 15 patients and their
356 informal caregivers. We will utilise a maximum variation sample, to choose patients (and
357 their informal caregivers) that differ in age, sex and previous/current treatment(s)

For peer review only

received. By using a varied sample, we aim to highlight important shared patterns that cut across cases and also discover unique or diverse variations from different participants [38].

Trial organisation

University of Leeds CTRU will manage all trial and data co-ordination according to the unit's well established standard operating procedures. All trial organisation will be conducted in-line with the principles of Good Clinical Practice.

Data collection and management

The BRIOChe trial data collection has been designed for remote data entry. However, all safety data will be recorded on paper case report forms and Patient Reported Outcome Measures (PROMs) on the relevant paper questionnaires, sent to and entered at CTRU. Participating centres will record and complete patient data on a trial specific database. The database has instantaneous validation checking. In addition, data management validation reports for missing and discrepant data.

All trial data is stored confidentially in a secure location at University of Leeds CTRU. Access to trial data will be restricted to CTRU staff and study team only, prior to the analysis and release of the trial results. Upon completion of the trial and publishing of results, researchers can request access to data by contacting members of the BRIOChe trial management group (TMG) or CTRU. All data will be stored and archived for a minimum of 25 years after trial completion.

For peer review only

For peer review only - http://bmjopen.bmj.com/site/about/guidelines.xhtml

Quality assurance

The radiotherapy quality assurance (QA) programme will be implemented by the National Cancer Research Institute (NCRI) Radiotherapy Trials Quality Assurance (RTTQA) group to ensure treatment is planned and delivered according to the trial protocol. RTTQA includes both benchmarking contouring and planning cases. A summary of the RTTQA requirements are provided in the BRIOChe radiotherapy outlining, planning, treatment delivery and QA guidelines. All sites must complete essential documentation and the site initiation process before a site can be activated. The site initiation will be an electronic process including an audio-visual recording. The video recorded site initiation presentation slides can be used for future training and reference at participating sites.

Trial monitoring

The BRIOChe trial will be monitored by the specific project delivery group at CTRU and the multi-disciplinary TMG. An independent data monitoring and ethics committee (DMEC) will be responsible for the safety and integrity of the patients and study, monitoring unblinded interim data. The DMEC will be formed of two independent clinical oncologists and one independent statistician, meeting at least annually, in addition to receiving 6-monthly safety reports. The DMEC members will advise the Trial Steering Committee (TSC) on any trial or safety concerns. The TSC have definitive control over the continuation of the trial. A DMEC charter has been developed including the roles and responsibilities, and communication processes.

Patient and Public Involvement

Patient and Public Involvement (PPI) has been an instrumental part of the development and conduct of the BRIOChe trial. The trial design was discussed with and presented to

For peer review only

PPI representatives. The trial question was felt to be of importance and the treatments and assessment schedule were considered acceptable.

The Trial Management Group includes a key patient representative, who provides ongoing and regular advice regarding trial conduct from the patient perspective.

Imaging sub-study

An imaging sub-study is planned between two of the recruiting centres (Leeds Teaching Hospitals NHS Trust and University College London Hospitals NHS Foundation Trust), to investigate the impact of re-irradiation and chemotherapy treatments on cerebral vascular changes and to improve understanding as to how these changes might be associated with both tumour response and normal tissue damage. The imaging sub-study is estimated to recruit 12-16 patients from the full BRIOChe trial. Sub-study participants who have been randomised to re-irradiation will have Arterial Spin Labelling (ASL) and T2 Dynamic Susceptibility Contrast (DSC) sequences added to the dedicated radiotherapy planning MRI. In addition, these participants will have ASL added to their routine follow up MRI scans (T2 DSC already included), to provide 'before' and 'after' re-irradiation information. Those sub-study participants randomised to chemotherapy will also have ASL and T2 DSC sequences added to their routine follow-up MRI to allow comparison between chemotherapy-treated and re-irradiated participants.

For peer review only

ETHICS AND DISSEMINATION

The trial obtained ethical approval from the Office for Research Ethics Committees Northern Ireland (ORECNI) (reference no. 20/NI/0070) and is registered in the ISRCTN registry (registration number 16052954). The trial is currently adhering to protocol version 4.0 (7/12/2022). Any protocol amendments will be submitted to MHRA and REC, all changes will be communicated with local sites.

There is no formal interim analysis. The final trial data will be analysed and reported approximately one year after the final participant is recruited. The final trial result manuscript will be written in accordance to ICMJE guidelines and submitted for publication to peer reviewed journals.

For peer review only

DISCUSSION

Despite the poor outcomes and high clinical need, there is a lack of active clinical trials for patients with recurrent GBM in the UK and no gold standard treatment at recurrence exists. Chemotherapy is often used in the UK, but outcomes are disappointing. Re-irradiation is recognised as an alternative treatment option and is listed as such in European and American treatment guidelines for patients with recurrent GBM, and in the current NICE guidelines for brain tumours [39-41]. Despite this, re-irradiation is less commonly utilised in the UK compared to other parts of the world. Re-irradiation offers a shorter course of treatment compared to chemotherapy and has a different toxicity profile, and so may be preferable for some patients, although the risks and benefits need further clarification. The BRIOChe trial aims to be a pragmatic, clinically relevant trial that evaluates re-irradiation as an alternative treatment option to chemotherapy for patients with recurrent GBM. While OS is the primary end point, HRQoL is a key secondary endpoint.

For peer review only - http://bmjopen.bmj.com/site/about/guidelines.xhtml

The trial design and treatment, specified in BRIOChe, intentionally reflects routine practice. Centres are able to use their usual lomustine-based chemotherapy regimen(s) thus reflecting the variety in practice across the UK. The frequently used and relatively well-tolerated hypofractionated schedule, 35Gy in 10 fractions, has been adopted for BRIOChe re-irradiation regimen [27-30] .

No organ at risk (OAR) constraints have been formally validated for GBM re-irradiation. Most GBM recurrences occur within the previous high dose region and so it is often necessary to assume that a degree of OAR recovery has occurred following the original course of radiotherapy if a meaningful dose is to be delivered to the re-irradiation target. As such, BRIOChe provides 'radiobiological' optimal constraints, allowing for 25% recovery from the original radiotherapy, based on limited evidence regarding post-irradiation recovery in neural structures [42-44]. In addition, where optimal constraints limit re-irradiation delivery, the more lenient mandatory constraints have been based on the RTOG-1205 trial [45]. Encouragingly since the opening of the BRIOChe trial, results from the RTOG1205 trial have been published indicating that re-irradiation, 35Gy in 10 fractions, as used in BRIOChe, in combination with bevacizumab, results in a median overall survival of 10.1 months [25]. In addition, re-irradiation was well tolerated [25]. However, re-irradiation alone was not investigated in FTOG1205, BRIOChe will investigate this further.

The minimal acceptable interval between completion of original radiotherapy and commencement of re-irradiation is also unknown and this interval varies amongst existing studies. We have adopted a minimum interval of 6 months from completion of first radiotherapy in BRIOChe, which is in-keeping with current practices in other countries [46] and international trials using the same re-irradiation schedule [31, 32]. An interval of at least

For peer review only - http://bmjopen.bmj.com/site/about/guidelines.xhtml

6 months also allows some time for OAR repair, should repair occur, and reduces the risk of mistaking pseudo-progression for true progression, which typically occurs within 3 months of radiotherapy [47].

While the patient numbers involved will not permit a direct comparison of survival outcomes in each arm, the BRIOChe trial aims to demonstrate that re-irradiation is an acceptable alternative treatment option for patients with recurrent GBM, including from the patient perspective. This in turn will allow the development of future novel approaches, including potential treatment intensification strategies using re-irradiation in combination with novel agents such as immunotherapies and radiosensitisers, to be explored in large-scale platform studies.

The BRIOChe study will provide valuable data for patients with recurrent GBM treated with re-irradiation and nitrosourea-based chemotherapy, forming a first step in improving treatment for this poor prognosis patient group.

For peer review only

For peer review only - http://bmjopen.bmj.com/site/about/guidelines.xhtml

AUTHOR CONTRIBUTIONS

Contributors to the conception, trial design and ongoing conduct: LM, FB, SCS, EH, SN, JW, SB, OA, HBe, HBu, SC, SF, JH, JL, AS, CP, FS, DSM.

Development of the protocol, trial documents and patient information sheet: EH, LM, SB, SN, FB, CP, SC, JL.

Writing manuscript: EH, LM.

Reviewed manuscript: all authors

All authors read and approved the final manuscript.

FUNDING, SPONSOR AND COORDINATION

BRIOChe is supported by The Jon Moulton Charity Trust (NA), and by Yorkshire Cancer Research (YCR), Centre for Early Phase Clinical Trials (L375PA). The imaging sub-study is supported by Cancer Research UK funding for the Leeds Radiotherapy Research Centre of Excellence (RadNet; C19942/A28832).

The study is coordinated by University of Leeds CTRU and sponsored by University of Leeds (GA18/118305). For enquires, trial contact *email:* BRIOCHE@leeds.ac.uk.

The sponsor and funders have had no influence on the BRIOChe trial study design, collection, management, analysis, and interpretation of data; and decision for publication. The sponsor and funder will review all manuscripts prior to publication.

For peer review only

COMPETING INTERESTS

FS reports grants from Cancer Research UK, during the conduct of the study. DSM reports grants from Jon Moulton Charitable Foundation, grants from Yorkshire Cancer Research, during the conduct of the study. LM reports grants from Jon Moulton Charity Trust, grants from Cancer Research UK, during the conduct of the study; grants from Yorkshire Cancer Research, outside the submitted work. SB reports grants from Jon Moulton Charitable Foundation, grants from Yorkshire Cancer Research, during the conduct of the study. SS reports advisory board activity for Bayer, CeCaVa Laboratory Research project support, Apollomics.

EXCLUSIVE LICENCE

I, the Submitting Author has the right to grant and does grant on behalf of all authors of the Work (as defined in the below author licence), an exclusive licence and/or a non-exclusive licence for contributions from authors who are: i) UK Crown employees; ii) where BMJ has agreed a CC-BY licence shall apply, and/or iii) in accordance with the terms applicable for US Federal Government officers or employees acting as part of their official duties; on a worldwide, perpetual, irrevocable, royalty-free basis to BMJ Publishing Group Ltd ("BMJ") its licensees and where the relevant Journal is co-owned by BMJ to the co-owners of the Journal, to publish the Work in BMJ Open and any other BMJ products and to exploit all rights, as set out in our licence.

The Submitting Author accepts and understands that any supply made under these terms is made by BMJ to the Submitting Author unless you are acting as an employee on behalf of your employer or a postgraduate student of an affiliated institution which is paying any applicable article publishing charge ("APC") for Open Access articles. Where the Submitting

For peer review only

Author wishes to make the Work available on an Open Access basis (and intends to pay the relevant APC), the terms of reuse of such Open Access shall be governed by a Creative Commons licence – details of these licences and which Creative Commons licence will apply to this Work are set out in our licence referred to above.

FIGURE LEGEND: Figure 1 - BRIOChe Trial schema

For peer review only

REFERENCES

1. Stupp, R., et al., *Radiotherapy plus concomitant and adjuvant temozolomide for glioblastoma.* New England journal of medicine, 2005. **352**(10): p. 987-996.
2. Gately, L., et al., *Life beyond a diagnosis of glioblastoma: a systematic review of the literature.* Journal of Cancer Survivorship, 2017. **11**(4): p. 447-452.
3. Mummudi, N. and R. Jalali, *Palliative care and quality of life in neuro-oncology.* F1000prime reports, 2014. **6**.
4. Arvold, N.D., et al., *Hospitalization burden and survival among older glioblastoma patients.* Neuro-oncology, 2014. **16**(11): p. 1530-1540.
5. Mukand, J.A., et al., *Incidence of neurologic deficits and rehabilitation of patients with brain tumors.* American journal of physical medicine & rehabilitation, 2001. **80**(5): p. 346-350.
6. Boele, F.W., et al., *Symptom management and quality of life in glioma patients.* CNS oncology, 2014. **3**(1): p. 37-47.
7. Sterckx, W., et al., *The impact of a high-grade glioma on everyday life: a systematic review from the patient's and caregiver's perspective.* European Journal of Oncology Nursing, 2013. **17**(1): p. 107-117.
8. Weller, M., et al., *Standards of care for treatment of recurrent glioblastoma—are we there yet?* Neuro-oncology, 2013. **15**(1): p. 4-27.
9. van Linde, M.E., et al., *Treatment outcome of patients with recurrent glioblastoma multiforme: a retrospective multicenter analysis.* Journal of neuro-oncology, 2017. **135**(1): p. 183-192.
10. Das, P., S. McCaffrey, and D. Conkey, *Re-evaluating the efficacy and toxicity of procarbazine, lomustine and vincrsitine (PCV) in relapsed glioblastoma in the concurrent/ adjuvant temozolamide era.* Journal of Cancer Prevention and Current Research, 2016. **6**(3): p. 1-6.
11. Taal, W., et al., *Single-agent bevacizumab or lomustine versus a combination of bevacizumab plus lomustine in patients with recurrent glioblastoma (BELOB trial): a randomised controlled phase 2 trial.* The lancet oncology, 2014. **15**(9): p. 943-953.
12. Carvalho, B.F., et al., *Second-line chemotherapy in recurrent glioblastoma: a 2-cohort study.* Oncology research and treatment, 2015. **38**(7-8): p. 348-354.
13. Brada, M., et al., *Temozolomide versus procarbazine, lomustine, and vincristine in recurrent high-grade glioma.* J Clin Oncol, 2010. **28**(30): p. 4601-4608.
14. Wick, W., et al., *Lomustine and bevacizumab in progressive glioblastoma.* New England Journal of Medicine, 2017. **377**(20): p. 1954-1963.
15. Rajan, B., et al., *Survival in patients with recurrent glioma as a measure of treatment efficacy: prognostic factors following nitrosourea chemotherapy.* European Journal of Cancer, 1994. **30**(12): p. 1809-1815.
16. Wick, W., et al., *Phase III study of enzastaurin compared with lomustine in the treatment of recurrent intracranial glioblastoma.* Journal of clinical oncology, 2010. **28**(7): p. 1168.
17. Lombardi, G., et al. *Updated results of REGOMA: A randomized, multicenter, controlled open-label phase II clinical trial evaluating regorafenib in relapsed glioblastoma (GBM) patients (PTS)*. in *Oral Presentation at EANO (Eurpoean Association of Neuro-Oncolgy) Annual conference*. 2018.
18. Dirven, L., et al., *Dutch Neuro-Oncology Group (LWNO). The impact of bevacizumab on health-related quality of life in patients treated for recurrent glioblastoma: results of the randomised controlled phase 2 BELOB trial.* Eur J Cancer, 2015. **51**(10): p. 1321-1330.
19. Pessina, F., et al., *Role of surgical resection in recurrent glioblastoma: prognostic factors and outcome evaluation in an observational study.* Journal of neuro-oncology, 2017. **131**(2): p. 377-384.
20. Franceschi, E., et al., *The effect of re-operation on survival in patients with recurrent glioblastoma.* Anticancer research, 2015. **35**(3): p. 1743-1748.

For peer review only - http://bmjopen.bmj.com/site/about/guidelines.xhtml

21. Gorlia, T., et al., *New prognostic factors and calculators for outcome prediction in patients with recurrent glioblastoma: a pooled analysis of EORTC Brain Tumour Group phase I and II clinical trials.* European journal of cancer, 2012. **48**(8): p. 1176-1184.

22. Kazmi, F., et al., *Re-irradiation for recurrent glioblastoma (GBM): a systematic review and meta-analysis.* Journal of neuro-oncology, 2019. **142**(1): p. 79-90.

23. Shanker, M., et al., *Re-irradiation for recurrent high-grade gliomas: a systematic review and analysis of treatment technique with respect to survival and risk of radionecrosis.* Neuro-Oncology Practice, 2019. **6**(2): p. 144-155.

24. Nieder, C., et al., *Improvement, clinical course, and quality of life after palliative radiotherapy for recurrent glioblastoma.* American journal of clinical oncology, 2008. **31**(3): p. 300-305.

25. Tsien, C.I., et al., *NRG Oncology/RTOG1205: a randomized phase II trial of concurrent bevacizumab and reirradiation versus bevacizumab alone as treatment for recurrent glioblastoma.* Journal of clinical oncology, 2023. **41**(6): p. 1285-1295.

26. Grant, R., et al., *OP01THE UK TOP 10 CLINICAL RESEARCH PRIORITIES IN NEURO-ONCOLOGY.* Neuro-Oncology, 2015. **17**(Suppl 8): p. viii16.

27. Klobukowski, L., et al., *A retrospective review of re-irradiating patients' recurrent high-grade gliomas.* Clinical Oncology, 2018. **30**(9): p. 563-570.

28. Fogh, S.E., et al., *Hypofractionated stereotactic radiation therapy: an effective therapy for recurrent high-grade gliomas.* Journal of clinical oncology, 2010. **28**(18): p. 3048.

29. Hudes, R.S., et al., *A phase I dose escalation study of hypofractionated stereotactic radiotherapy as salvage therapy for persistent or recurrent malignant glioma.* International Journal of Radiation Oncology* Biology* Physics, 1999. **43**(2): p. 293-298.

30. Tsien, C., et al., *ACTR-32. NRG ONCOLOGY RTOG 1205: randomized phase II trial of concurrent bevacizumab and re-irradiation vs. bevacizumab alone as treatment for recurrent glioblastoma.* Neuro-Oncology, 2019. **21**(Suppl 6): p. vi20.

31. RTOG 1205. *Randomized phase II trial of concurrent bevacizumab and re-irradiation versus bevacizumab alone as treatment of recurrent glioblastoma.* 2016; Available from: https://clinicaltrials.gov/ct2/show/NCT01730950.

32. AstraZeneca, *AZD1390 Radiation Procedure Manual. Version 1.0.* 2017.

33. Sargent, D.J., V. Chan, and R.M. Goldberg, *A three-outcome design for phase II clinical trials.* Controlled clinical trials, 2001. **22**(2): p. 117-125.

34. Abraha, I. and A. Montedori, *Modified intention to treat reporting in randomised controlled trials: systematic review.* Bmj, 2010. **340**.

35. Batchelor, T.T., et al., *Phase III randomized trial comparing the efficacy of cediranib as monotherapy, and in combination with lomustine, versus lomustine alone in patients with recurrent glioblastoma.* Journal of Clinical Oncology, 2013. **31**(26): p. 3212.

36. Boiardi, A., et al., *Efficacy of '8-drugs-in-one-day'combination in treatment of recurrent GBM patients.* Journal of neuro-oncology, 1992. **12**(2): p. 153-158.

37. Fereday, J. and E. Muir-Cochrane, *Demonstrating rigor using thematic analysis: A hybrid approach of inductive and deductive coding and theme development.* International journal of qualitative methods, 2006. **5**(1): p. 80-92.

38. Patton, M., *Pattern, theme, and content analysis*, in *Qualitative research & evaluation methods*. 2002, Sage Publications Ltd, London. p. 452-471.

39. Cabrera, A.R., et al., *Radiation therapy for glioblastoma: executive summary of an American Society for Radiation Oncology evidence-based clinical practice guideline.* Practical radiation oncology, 2016. **6**(4): p. 217-225.

40. Stupp, R., et al., *High-grade glioma: ESMO Clinical Practice Guidelines for diagnosis, treatment and follow-up.* Annals of Oncology, 2014. **25**: p. iii93-iii101.

41. NICE, N.I.f.H.a.C.E., *Brain tumours (primary) and brain metastases in adults.* NICE guideline [NG99], 2018.

42. Kirkpatrick, J.P., A.J. van der Kogel, and T.E. Schultheiss, *Radiation dose–volume effects in the spinal cord.* International Journal of Radiation Oncology* Biology* Physics, 2010. **76**(3): p. S42-S49.

For peer review only

For peer review only - http://bmjopen.bmj.com/site/about/guidelines.xhtml

43. Ang, K.K., et al., *Extent and kinetics of recovery of occult spinal cord injury.* International Journal of Radiation Oncology* Biology* Physics, 2001. **50**(4): p. 1013-1020.

44. Flickinger, J.C., M. Deutsch, and L.D. Lunsford, *Repeat megavoltage irradiation of pituitary and suprasellar tumors.* International Journal of Radiation Oncology* Biology* Physics, 1989. **17**(1): p. 171-175.

45. Tsien, C., et al., *Randomized phase II trial of re-irradiation and concurrent bevacizumab versus bevacizumab alone as treatment for recurrent glioblastoma (NRG Oncology/RTOG 1205): initial outcomes and RT plan quality report.* International Journal of Radiation Oncology Biology and Physics, 2019. **105**(1).

46. Post, C.C., et al., *Patterns of re-irradiation for recurrent gliomas and validation of a prognostic score.* Radiotherapy and Oncology, 2019. **130**: p. 156-163.

47. De Wit, M., et al., *Immediate post-radiotherapy changes in malignant glioma can mimic tumor progression.* Neurology, 2004. **63**(3): p. 535-537.

For peer review only

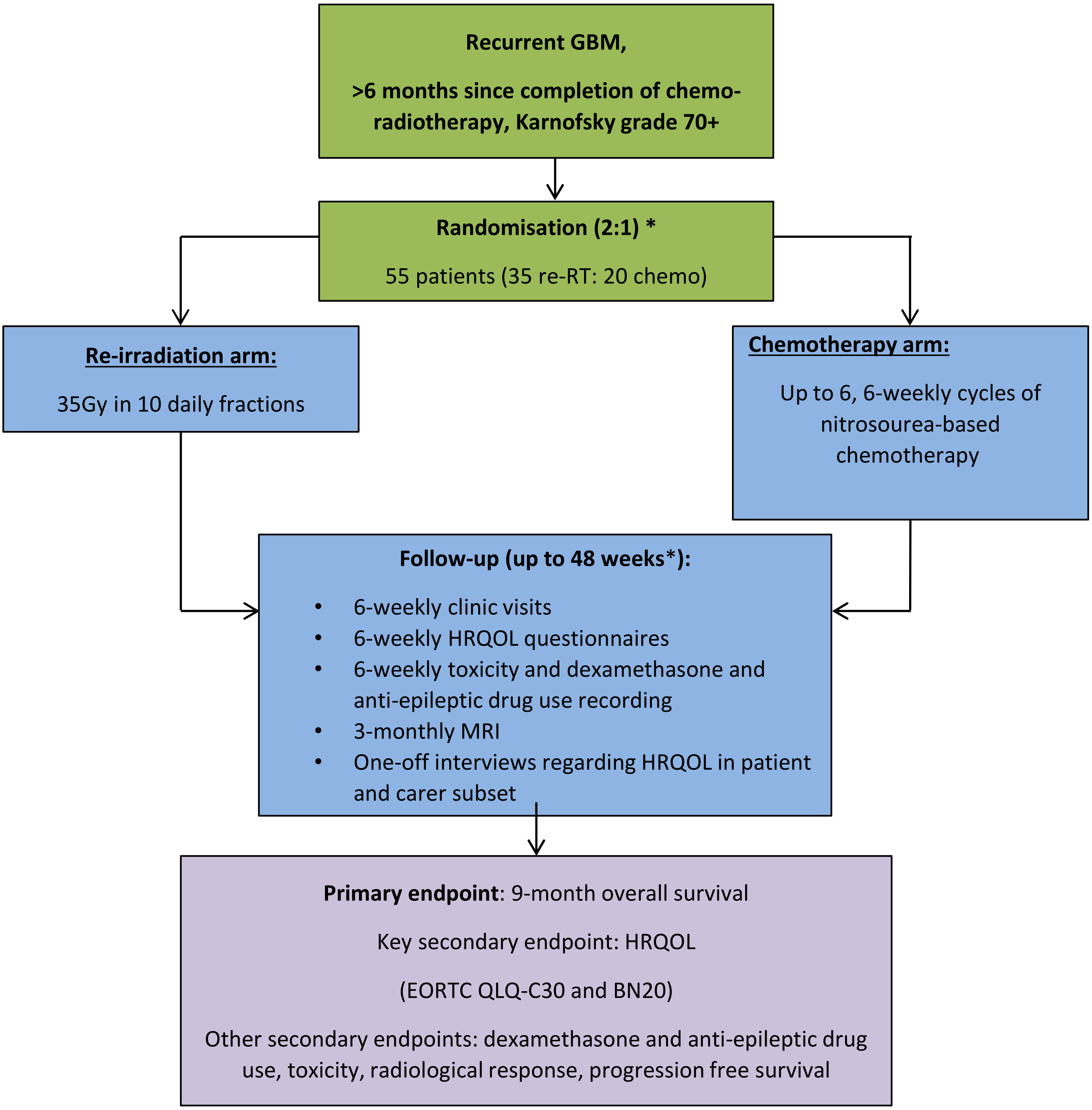

**allocation ratio originally 1:1, ratio was adapted to 2:1 during the recruitment phase*

EORTC QLQ-C30 and BN20, European Organization for Research and Treatment of Cancer Quality of life questionnaire core 30 and Brain Cancer module 20; GBM, glioblastoma multiforme; HRQoL, health-related quality of life; MRI, magnetic resonance imaging;

SUPPLEMENTARY FILE 1 – Full inclusion exclusion criteria

Inclusion criteria	Exclusion criteria
1. Histologically proven diagnosis of GBM with consistent molecular pathology, based on original pathology.	1. Pregnant (positive pregnancy test) or lactating
2. First recurrence of GBM, with contrast enhancing disease, following primary treatment (i.e. no previous systemic therapy or re-irradiation for recurrence).	2. Critical normal brain structures treated above usual tolerance during initial radiotherapy
3. Agreement of a Consultant Neuro-Radiologist within multi-disciplinary team meeting that imaging changes are in keeping with recurrence and not pseudo-progression.	3. Recurrence with leptomeningeal disease or only leptomeningeal disease
4. Randomisation must be performed within 21 days of the MRI that confirms recurrence.	4. Recurrence defined by non-enhancing disease only
5. ≥6 months since completion of primary radiotherapy	5. More than three enhancing lesions present on MRI or multi-focal recurrence
6. Prior history of standard dose, conventionally fractionated CNS radiotherapy	6. IDH1/2 mutant tumours on original pathology (to avoid unbalance between arms).
7. As a minimum patients will have completed at least two weeks of temozolomide, concurrent with their original radiotherapy.	7. GBM with known features of PXA, BRAF mutations or 1p19q co-deletion (on original pathology or updated pathology if available)
• Up to and including three enhancing lesions:<br>○ In cases of a single recurrent enhancing lesion:<br>▪ Predicted re-irradiation GTV<75cm^3 and<br>▪ Maximum diameter of enhancing disease must be ≤6cm.<br>○ In cases of multiple (i.e. two or three) discrete recurrent enhancing lesions:<br>▪ The total (i.e. combined) predicted re-irradiation GTV must be <50cm^3 and lesions must be clustered in a similar brain region such that PTVs are anticipated to be adjacent or overlapping and<br>▪ Maximum diameter of the combined enhancing disease, across all enhancing lesions (including any gaps between), must be ≤6cm.	8. Prior invasive malignancy (except non-melanomatous skin cancer), unless disease free for a minimum of one year.

For peer review only - http://bmjopen.bmj.com/site/about/guidelines.xhtml

8. Karnofsky Performance Status 70+	9. Severe active co-morbidity making patient unsuitable for chemotherapy or re-irradiation
9. Adequate hematologic, renal, and hepatic function	10. Prior allergic reaction to nitrosoureas
10. Patients who have had surgery for first recurrence may also be included provided there is residual enhancing disease on the immediate post-operative MRI or if enhancing disease develops on subsequent follow-up imaging, provided no prior systemic therapy or re-irradiation for recurrence has been given.	11. Any recognised genetic syndromes causing sensitivity to radiotherapy
11. Patients must have recovered from the prior effects of therapy	12. Patient unwilling/ unable to attend for follow up in the radiotherapy centre
12. Medical history and physical examination, including CNS examination, must be performed within 14 days prior to randomisation.	13. Contra-indication to MRI or gadolinium
13. Female participants of child-bearing potential must agree to be pregnancy screened.	14. Previous radiotherapy dose distribution unavailable
14. Patients must be able to swallow oral medication.	15. Previous systemic therapy or re-irradiation for recurrent GBM
15. Patients must be able to provide study-specific informed consent.	16. Spinal or infratentorial disease
16. Age 18 or over.	
17. Patients should be able to start treatment within 21 days of randomisation and must start within 28 days of randomisation.	

Coeliac disease is usually considered a contra-indication to lomustine as there is wheat within the capsule. For patients with recurrent GBM, the individual clinician may decide whether or not this is a relevant exclusion criteria.

For peer review only

Delete this line, then print on Trust/Hospital headed paper

Participant ID:	Initials:
Date of Birth:	NHS/Hospital Number:
EudraCT Number: 2019-004053-91	Principal Investigator:

BRIOChe

SAMPLE

PARTICIPANT CONSENT FORM

Please initial each box

1. I confirm that I have read and understood the information sheet for the above study and have had the opportunity to ask questions. ☐

2. I understand that my participation in this study is voluntary and that I am free to withdraw at any time without my medical care or legal rights being affected. I understand that even if I withdraw from the above study, the data and samples collected from me will be used in analysing the results of the study and in some cases further information about any unwanted effects of my treatment may need to be collected by the study team. ☐

3. I understand that my healthcare records may be looked at by authorised individuals from the study team, regulatory bodies or Sponsor in order to check that the study is being carried out correctly. ☐

4. I agree to allow any information or results arising from this study to be used for healthcare and/or further medical research upon the understanding that my identity will remain anonymous wherever possible. This includes the use of my radiotherapy data and imaging data for future analysis. ☐

5. I understand that if I lose capacity, further information about any unwanted effects of my treatment may need to be collected by the study team.

6. If selected, I agree to take part in a semi-structured interview on my experiences of participating in the trial and my health-related quality of life, where a member of the research team may approach me directly to schedule a suitable day and time. I agree to these interviews being audio recorded.

7. I agree to consent to my anonymised trial data to be viewed if required by 3rd parties outside the UK / EU etc.

8. I agree to a copy of this Consent Form being sent to the Clinical Trials Research Unit.

9. I agree that my GP, or any other doctor treating me, will be notified of my participation in this study.

10. I agree to take part in the study.

The following points are **OPTIONAL.**

Title: Consent for potential tissue collection and use in future research (if you do not wish to give this permission do not initial the boxes – you can still participate in the trial)

Please initial each box

	Yes	No
I give my permission for stored tumour tissue sample that was removed during my operation and was not needed for routine diagnosis and treatment to be collected and used for future translational research purposes as described in the information sheet for the above trial if this becomes relevant in the future.		
I understand how the tissue sample will be collected, that giving samples is voluntary and that I'm free to withdraw my approval for use of the samples at any time without giving a reason and without my medical care or legal rights being affected.		

SAMPLE
For peer review only

Patient:
Signature……………………………………………………………………………

Name (block capitals)………………………………………………………………

Date……………………………………………………………………………………

Investigator:

I have explained the study to the above named patient and he/she has indicated his/her willingness to participate.

Signature……………………………………………………………………………

Name (block capitals)………………………………………………………………

Date……………………………………………………………………………………

(If used)Translator:

Signature……………………………………………………………………………

Name (block capitals)………………………………………………………………

Date……………………………………………………………………………………

(1 copy for patient; 1 for the CTRU; 1 held in patient notes;

SAMPLE
For peer review only

SPIRIT

STANDARD PROTOCOL ITEMS: RECOMMENDATIONS FOR INTERVENTIONAL TRIALS

SPIRIT 2013 Checklist: Recommended items to address in a clinical trial protocol and related documents*

Section/item	Item No	Description	Addressed on page number
**Administrative information**			
Title	1	Descriptive title identifying the study design, population, interventions, and, if applicable, trial acronym	1 ____________
Trial registration	2a	Trial identifier and registry name. If not yet registered, name of intended registry	4, 23___________
	2b	All items from the World Health Organization Trial Registration Data Set	Many___________
Protocol version	3	Date and version identifier	23 ____________
Funding	4	Sources and types of financial, material, and other support	4, 26___________
Roles and responsibilities	5a	Names, affiliations, and roles of protocol contributors	1, 26 __________
	5b	Name and contact information for the trial sponsor	26 ____________
	5c	Role of study sponsor and funders, if any, in study design; collection, management, analysis, and interpretation of data; writing of the report; and the decision to submit the report for publication, including whether they will have ultimate authority over any of these activities	26 ____________
	5d	Composition, roles, and responsibilities of the coordinating centre, steering committee, endpoint adjudication committee, data management team, and other individuals or groups overseeing the trial, if applicable (see Item 21a for data monitoring committee)	20, 21 _________

For peer review only

For peer review only - http://bmjopen.bmj.com/site/about/guidelines.xhtml

Introduction

Background and rationale	6a	Description of research question and justification for undertaking the trial, including summary of relevant studies (published and unpublished) examining benefits and harms for each intervention	6,7, 8_____
	6b	Explanation for choice of comparators	6, 7__________
Objectives	7	Specific objectives or hypotheses	11 _________
Trial design	8	Description of trial design including type of trial (e.g., parallel group, crossover, factorial, single group), allocation ratio, and framework (e.g., superiority, equivalence, non-inferiority, exploratory)	9 ___________

Methods: Participants, interventions, and outcomes

Study setting	9	Description of study settings (e.g., community clinic, academic hospital) and list of countries where data will be collected. Reference to where list of study sites can be obtained	9 ________
Eligibility criteria	10	Inclusion and exclusion criteria for participants. If applicable, eligibility criteria for study centres and individuals who will perform the interventions (e.g., surgeons, psychotherapists)	13, Table 1, Supplementary file 1
Interventions	11a	Interventions for each group with sufficient detail to allow replication, including how and when they will be administered	15__________
	11b	Criteria for discontinuing or modifying allocated interventions for a given trial participant (e.g., drug dose change in response to harms, participant request, or improving/worsening disease)	15, 16_________
	11c	Strategies to improve adherence to intervention protocols, and any procedures for monitoring adherence (e.g., drug tablet return, laboratory tests)	14,15 ________
	11d	Relevant concomitant care and interventions that are permitted or prohibited during the trial	N/A________
Outcomes	12	Primary, secondary, and other outcomes, including the specific measurement variable (e.g., systolic blood pressure), analysis metric (e.g., change from baseline, final value, time to event), method of aggregation (e.g., median, proportion), and time point for each outcome. Explanation of the clinical relevance of chosen efficacy and harm outcomes is strongly recommended	11 _____

For peer review only - http://bmjopen.bmj.com/site/about/guidelines.xhtml

Participant timeline	13	Time schedule of enrolment, interventions (including any run-ins and washouts), assessments, and visits for participants. A schematic diagram is highly recommended (see Figure)	Table 2, Figure 1
Sample size	14	Estimated number of participants needed to achieve study objectives and how it was determined, including clinical and statistical assumptions supporting any sample size calculations	14_______
Recruitment	15	Strategies for achieving adequate participant enrolment to reach target sample size	12______

Methods: Assignment of interventions (for controlled trials)

Allocation:

Sequence generation	16a	Method of generating the allocation sequence (e.g., computer-generated random numbers), and list of any factors for stratification. To reduce predictability of a random sequence, details of any planned restriction (eg, blocking) should be provided in a separate document that is unavailable to those who enrol participants or assign interventions	13, 14 __________
Allocation concealment mechanism	16b	Mechanism of implementing the allocation sequence (e.g., central telephone; sequentially numbered, opaque, sealed envelopes), describing any steps to conceal the sequence until interventions are assigned	13, 14 ___________
Implementation	16c	Who will generate the allocation sequence, who will enrol participants, and who will assign participants to interventions	11, 12, 13______
Blinding (masking)	17a	Who will be blinded after assignment to interventions (e.g., trial participants, care providers, outcome assessors, data analysts), and how	N/A _________
	17b	If blinded, circumstances under which unblinding is permissible, and procedure for revealing a participant's allocated intervention during the trial	N/A__________

Methods: Data collection, management, and analysis

Data collection methods	18a	Plans for assessment and collection of outcome, baseline, and other trial data, including any related processes to promote data quality (e.g., duplicate measurements, training of assessors) and a description of study instruments (e.g., questionnaires, laboratory tests) along with their reliability and validity, if known. Reference to where data collection forms can be found, if not in the protocol	10, 11, (Figure 1)

	18b	Plans to promote participant retention and complete follow-up, including list of any outcome data to be collected for participants who discontinue or deviate from intervention protocols	16__________
Data management	19	Plans for data entry, coding, security, and storage, including any related processes to promote data quality (e.g., double data entry; range checks for data values). Reference to where details of data management procedures can be found, if not in the protocol	20 _________
Statistical methods	20a	Statistical methods for analysing primary and secondary outcomes. Reference to where other details of the statistical analysis plan can be found, if not in the protocol	18,19______
	20b	Methods for any additional analyses (e.g., subgroup and adjusted analyses)	18,19_______
	20c	Definition of analysis population relating to protocol non-adherence (e.g., as randomised analysis), and any statistical methods to handle missing data (e.g., multiple imputation)	18 _________
**Methods: Monitoring**			
Data monitoring	21a	Composition of data monitoring committee (DMC); summary of its role and reporting structure; statement of whether it is independent from the sponsor and competing interests; and reference to where further details about its charter can be found, if not in the protocol. Alternatively, an explanation of why a DMC is not needed	21 __________
	21b	Description of any interim analyses and stopping guidelines, including who will have access to these interim results and make the final decision to terminate the trial	21, 23 ________
Harms	22	Plans for collecting, assessing, reporting, and managing solicited and spontaneously reported adverse events and other unintended effects of trial interventions or trial conduct	17 ____
Auditing	23	Frequency and procedures for auditing trial conduct, if any, and whether the process will be independent from investigators and the sponsor	20_________
**Ethics and dissemination**			
Research ethics approval	24	Plans for seeking research ethics committee/institutional review board (REC/IRB) approval	23 ___________

Protocol amendments	25	Plans for communicating important protocol modifications (e.g., changes to eligibility criteria, outcomes, analyses) to relevant parties (e.g., investigators, REC/IRBs, trial participants, trial registries, journals, regulators)	23____________
Consent or assent	26a	Who will obtain informed consent or assent from potential trial participants or authorised surrogates, and how (see Item 32)	11 ___________
	26b	Additional consent provisions for collection and use of participant data and biological specimens in ancillary studies, if applicable	N/A__________
Confidentiality	27	How personal information about potential and enrolled participants will be collected, shared, and maintained in order to protect confidentiality before, during, and after the trial	20 ________
Declaration of interests	28	Financial and other competing interests for principal investigators for the overall trial and each study site	26 ___________
Access to data	29	Statement of who will have access to the final trial dataset, and disclosure of contractual agreements that limit such access for investigators	20 ________
Ancillary and post-trial care	30	Provisions, if any, for ancillary and post-trial care, and for compensation to those who suffer harm from trial participation	N/A ________
Dissemination policy	31a	Plans for investigators and sponsor to communicate trial results to participants, healthcare professionals, the public, and other relevant groups (e.g., via publication, reporting in results databases, or other data sharing arrangements), including any publication restrictions	23 _____
	31b	Authorship eligibility guidelines and any intended use of professional writers	23 __________
	31c	Plans, if any, for granting public access to the full protocol, participant-level dataset, and statistical code	20_________

Appendices

Informed consent materials	32	Model consent form and other related documentation given to participants and authorised surrogates	Supplementary fie 2 _________
Biological specimens	33	Plans for collection, laboratory evaluation, and storage of biological specimens for genetic or molecular analysis in the current trial and for future use in ancillary studies, if applicable	_N/A_________

For peer review only - http://bmjopen.bmj.com/site/about/guidelines.xhtml

*It is strongly recommended that this checklist be read in conjunction with the SPIRIT 2013 Explanation & Elaboration for important clarification on the items. Amendments to the protocol should be tracked and dated. The SPIRIT checklist is copyrighted by the SPIRIT Group under the Creative Commons "Attribution-NonCommercial-NoDerivs 3.0 Unported" license.

For peer review only

BMJ Open

Brain Re-Irradiation Or Chemotherapy: a phase II randomised trial of re-irradiation and chemotherapy in patients with recurrent glioblastoma (BRIOChe): Protocol for a multi-centre open label randomised trial

Journal:	*BMJ Open*
Manuscript ID	bmjopen-2023-078926.R2
Article Type:	Protocol
Date Submitted by the Author:	19-Jan-2024

Complete List of Authors:	Hudson, Eleanor; University of Leeds, Leeds Institute of Clinical Trials Research<br>Noutch, Samantha; University of Leeds, Leeds Institute of Clinical Trials Research<br>Webster, Joanne; University of Leeds, Leeds Institute of Clinical Trials Research<br>Brown, Sarah; University of Leeds, Clinical Trials Research Unit<br>Boele, Florien; University of Leeds, Leeds Institute of Medical Research at St James's; University of Leeds, Leeds Institute of Health Sciences<br>Al-Salihi, Omar; Guy's and St Thomas' Hospitals NHS Trust<br>Baines, Helen; Mount Vernon Cancer Centre, National Radiotherapy Trials QA (RTTQA) Group<br>Bulbeck, Helen; brainstrust,<br>Currie, Stuart; Leeds General Infirmary, Department of Radiology<br>Fernandez, Sharon; University of Leeds, Leeds Institute of Medical Research at St James's<br>Hughes, Jane; University of Leeds, Leeds Institute of Medical Research at St James's<br>Lilley, John; Leeds Institute of Cancer and Pathology, Department of Medical Physics<br>Smith, Alexandra; University of Leeds, Leeds Institute of Clinical Trials Research<br>Parbutt, Catherine; Leeds Teaching Hospitals NHS Trust, Pharmacy<br>Slevin, Finbar; Leeds Institute of Cancer and Pathology, Department of Clinical Oncology; University of Leeds, Leeds Institute of Medical Research at St James's<br>Short, Susan; Leeds Institute of Cancer and Pathology, Department of Clinical Oncology; University of Leeds, Leeds Institute of Medical Research at St James's<br>Sebag-Montefiore, David ; University of Leeds Faculty of Medicine and Health, Institute of Cancer and Pathology<br>Murray, Louise; Leeds Institute of Cancer and Pathology, Department of Clinical Oncology; University of Leeds, Leeds Institute of Medical Research at St James's

**Primary Subject Heading**:	Oncology

Secondary Subject Heading:	Oncology
Keywords:	Radiation oncology < RADIOTHERAPY, RADIOTHERAPY, ONCOLOGY, Neurological oncology < NEUROLOGY

SCHOLARONE™ Manuscripts

Brain Re-Irradiation Or Chemotherapy: a phase II randomised trial of re-irradiation and chemotherapy in patients with recurrent glioblastoma (BRIOChe): Protocol for a multi-centre open label randomised trial

Authors:

Eleanor Hudson[1], Samantha Noutch[1], Joanne Webster[1], Sarah R. Brown[1], Florien W. Boele[2,3], Omar Al-Salihi[4], Helen Baines[5], Helen Bulbeck[6], Stuart Currie[7], Sharon Fernandez[2] Jane Hughes[2], John Lilley[8], Alexandra Smith[1], Catherine Parbutt[9], Finbar Slevin[2,10], Susan C. Short[2,10], David Sebag-Montefiore[2], Louise Murray[2,10]

Author affiliation:

[1]Clinical Trials Research Unit, Leeds Institute of Clinical Trials Research, University of Leeds, Leeds, United Kingdom

[2]Leeds Institute of Medical Research at St James's, University of Leeds, Leeds, United Kingdom

[3]Leeds Institute of Health Sciences, Faculty of Medicine and Health, University of Leeds, Leeds, UK

[4]Guy's and St Thomas Hospital, London, United Kingdom

[5]National Radiotherapy Trials QA (RTTQA) Group, United Kingdom

[6]Patient Advocate, United Kingdom

[7]Department of Radiology, Leeds General Infirmary, Leeds, United Kingdom

[8]Department of Medical Physics, Leeds Cancer Centre, Leeds, United Kingdom

[9]Pharmacy Department, St James's University Hospital, Leeds, United Kingdom

[10]Department of Clinical Oncology, Leeds Cancer Centre, Leeds, United Kingdom

For peer review only

Corresponding author: Dr. Louise Murray

L.J.Murray@leeds.ac.uk

Leeds Institute of Medical Research at St James's,

University of Leeds,

St James's University Hospital,

Harehills

Leeds,

LS9 7LP

+44 113 206 7854

Word count (excluding title page, abstract, references, figures and tables):

4180-128-234-115 = 3,703

For peer review only

ABSTRACT (300 word count)

Introduction

Glioblastoma (GBM) is the most common adult primary malignant brain tumour. The condition is incurable and, despite aggressive treatment at first presentation, almost all tumours recur after a median of 7 months. The aim of treatment at recurrence is to prolong survival and maintain health-related quality of life (HRQoL). Chemotherapy is typically employed for recurrent GBM, often using nitrosourea-based regimens. However, efficacy is limited, with reported median survivals between 5 and 9 months. Although less commonly used in the United Kingdom (UK), there is growing evidence that re-irradiation may produce survival outcomes at least similar to nitrosourea-based chemotherapy. However, there remains uncertainty as to the optimum approach and there is a paucity of available data, especially with regards to HRQoL. BRIOChe aims to assess re-irradiation, as an acceptable treatment option for recurrent IDH-wild-type GBM.

Methods and analysis

BRIOChe is a phase II, multi-centre, open-label, randomised trial in patients with recurrent GBM. The trial uses a Sargent's three-outcome design and will recruit approximately 55 participants from 10-15 UK radiotherapy sites, allocated (2:1) to receive re-irradiation (35 Gy in 10 daily fractions) or nitrosourea-based chemotherapy (up to six, 6-weekly cycles). The primary endpoint is overall survival rate for re-irradiation patients at 9 months. There will be no formal statistical comparison between treatment arms for the decision-making primary analysis. The chemotherapy arm will be used for calibration purposes, to collect concurrent data to aid interpretation of results. Secondary outcomes include HRQoL, dexamethasone

For peer review only

requirement, anti-epileptic drug requirement, radiological response, treatment compliance, acute and late toxicities, progression free survival.

Ethics and dissemination

BRIOChe obtained ethical approval from Office for Research Ethics Committees Northern Ireland (reference no. 20/NI/0070). Final trial results will be published in peer reviewed journals and adhere to the to ICMJE guidelines.

Registration details: BRIOChe (ISRCTN: 16052954; registered 25/02/2021)

For peer review only

For peer review only - http://bmjopen.bmj.com/site/about/guidelines.xhtml

STRENGTHS AND WEAKNESSES (up to 5)

- BRIOChe is multi-centre randomised trial exploring re-irradiation and chemotherapy for recurrent GBM.
- The trial will collect much needed survival and health related quality of life (HRQoL) data for patients with recurrent GBM treated with re-irradiation.
- BRIOChe focuses on the underserved population of recurrent GBM patients, for whom there is a significant lack of treatment options and UK-based trials.
- The trial design and decision criteria are based on available historical data at the time of development, where patient populations may differ from those in the BRIOChe trial.
- The trial does not provide formal comparative evidence between treatment arms. Rather the parallel concurrent control arm provides essential randomised data for benchmarking and interpretation.

For peer review only

INTRODUCTION

Glioblastoma

Glioblastoma (GBM) is the most common adult primary malignant brain tumour, with an incidence of 5/100,000 per year in England. Prognosis is poor, with an average survival of 12 to 15 months [1].

GBM is incurable, with treatment from the point of diagnosis aiming to improve survival, delay disease progression and manage symptoms/preserve HRQoL. Optimal treatment at first presentation is aggressive, including debulking surgery (if feasible), chemo-radiotherapy and adjuvant chemotherapy. However, almost all patients will relapse within the brain, with a median progression free survival (PFS) of approximately 7 months [1].

The burden placed on patients, their informal caregivers and the healthcare system as a result of neurological disability and the care requirements for patients with brain tumours is significant [2-4]. GBM is often accompanied by debilitating physical and neurological symptoms, such as paresis, sensory loss, seizures, fatigue, mood issues, and cognitive deficits [5-7]. This symptom burden can affect patients' ability to function independently and negatively impact on health-related quality of life (HRQoL) for both patients and their informal caregivers.

Recurrent glioblastoma

The optimal treatment for recurrent GBM is undefined and treatment decisions are made on a case-by-case basis, based on the site, volume and time of recurrence in relation to the original diagnosis, with consideration for patient age, co-morbidities and performance status.

In the UK, chemotherapy is commonly employed in the setting of recurrent GBM, typically using nitrosourea-based regimens. These DNA alkylating agents are highly lipophilic, and are

For peer review only

therefore able to penetrate the blood brain barrier, making them suitable for the treatment of brain tumours. Nitrosourea-combination regimens have not proven superior to single agent nitrosoureas, resulting in variations in practice as to which regimens are employed [8]. Efficacy is limited, with reported median overall survivals (OS) of between 5 and 9 months [9-17]. Side effects may include fatigue, nausea, vomiting, anorexia, constipation, paraesthesia, rash and myelosuppression. There is currently a relative lack of HRQoL data for patients receiving nitrosourea-based chemotherapy for recurrent GBM [13, 16, 18], making it difficult to determine whether the benefits of chemotherapy outweigh its burdens.

Surgery is another treatment option at point of recurrence, however, it is usually reserved for a minority of excellent performance patients with well-localised disease, although high-level evidence to support its role is lacking [9, 19-21].

Re-irradiation for recurrent glioblastoma

Re-irradiation is a further treatment option for recurrent GBM, although at present it is not commonly used in the UK. A meta-analysis of re-irradiation for recurrent GBM [22] included 50 studies of re-irradiation, of which 35 employed external beam radiotherapy (EBRT; the remainder used brachytherapy). Of the patients who received EBRT, 6-month and 12-month overall survival (OS) was 70% and 34% respectively [22]. Progression free survival (PFS) was 40% and 16% at 6 and 12-months, respectively. Grade 3+ toxicity was reported in 7% of patients. Overall, only 13 of the studies were of prospective design and only 9 were considered by the authors to be of good quality. Similarly, a systematic review of re-irradiation in GBM presented comparable outcomes based on a pooled, population-weighted analysis, where mean adjusted OS following hypofractionated EBRT was 10.1 months (95% confidence interval (CI) 9.7-10.5 months) and the rate of radionecrosis was 7.1% (95% CI: 6.6-7.7%) [23]. Despite the promising outcomes reported after re-irradiation, the poor quality of existing evidence highlights the urgent need for a high quality, prospective, randomised assessment

For peer review only

of re-irradiation, including HRQoL assessment [23, 24]. More recently, the phase II RTOG1205 trial, which randomised patients with recurrent GBM between bevacizumab (not routinely available in the UK) and bevacizumab plus re-irradiation 35Gy in 10 fractions, demonstrated a median overall survival of 10.1 months in the combination arm and improved progression free survival compared to bevacizumab alone [25]. Treatment was also well tolerated, with 5% grade 3+ acute toxicities and no delayed high grade adverse events reported.

Rationale for study

While the limited existing evidence suggests that re-irradiation has a potential positive impact on overall survival and HRQoL, to date, no trial has randomised patients with recurrent GBM between re-irradiation and chemotherapy. Addressing this research gap is a top priority for patients, informal caregivers, and healthcare professionals [26].

The BRIOChe phase II prospective trial will randomise patients with recurrent GBM to re-irradiation (35 Gy in 10 fractions, a commonly adopted re-irradiation regimen [27-32]), or nitrosourea-based chemotherapy. The aims will be to explore the efficacy and HRQoL impact of re-irradiation. This study will collect concurrent data for patients receiving chemotherapy for validation ('benchmarking') of outcomes. The study is not designed to statistically compare the two arms for the primary outcome of survival, which would require a larger number of patients. By establishing the feasibility of randomisation, and providing prospective controlled data on survival and HRQoL impacts associated with re-irradiation, the BRIOChe trial aims to demonstrate re-irradiation as an acceptable alternative treatment option for patients with recurrent GBM, guiding the design of future larger scale randomised trials.

For peer review only

METHODS AND ANALYSIS

Design and aim

BRIOChe is a phase II, multi-centre, open-label, randomised trial in patients with recurrent GBM. The aim of the study is to assess re-irradiation as an alternative treatment to chemotherapy in this group of patients. BRIOChe uses a Sargent's three-outcome, single-stage, single-arm design, with the inclusion of a chemotherapy calibration arm for concurrent data for benchmarking. Following an amendment due to slow recruitment, the trial will now recruit approximately 55 participants from 10-15 UK radiotherapy sites. The original sample size was 70 based on a 1:1 allocation ratio, to receive either re-irradiation or nitrosourea-based chemotherapy (lomustine +/- procarbazine +/- vincristine). The allocation ratio was adapted during recruitment to 2:1, further details are presented in the sample size section.

The trial schema is presented in Figure 1.

For peer review only

For peer review only - http://bmjopen.bmj.com/site/about/guidelines.xhtml

Trial objectives

The primary objective of BRIOChe is to assess the proportion of participants alive at 9 months post start of treatment in the re-irradiation arm.

The key secondary objective of BRIOChe is to evaluate the effect of re-irradiation on HRQoL. Questionnaires to be completed by participants include the European Organization for Research and Treatment of Cancer (EORTC) Quality of life questionnaire core 30 (QLQ-C30) and Brain Cancer module (BN20). HRQoL will also be explored in greater detail through one-off semi-structured qualitative interviews in a subsample of patients and their informal caregivers.

Additional secondary objectives include evaluating:

- Dexamethasone requirement
- Anti-epileptic drug requirement
- Radiological response in accordance with Response Assessment in Neuro-Oncology (RANO) criteria
- Treatment compliance
- Acute (measured from randomisation to 12 weeks post end of treatment of treatment) and late toxicities (after 12 weeks post end of treatment) as per Common Terminology Criteria for Adverse Events (CTCAE V.5.0.); radionecrosis is a late toxicity of particular interest
- PFS
- OS

Study population

All inclusion/exclusion criteria must be met and written informed consent obtained prior to recruitment (Key criteria summarised in Table 1); full criteria (Supplementary File 1) and a sample patient consent form (Supplementary File 2) are included as Supplementary Material.

For peer review only

For peer review only - http://bmjopen.bmj.com/site/about/guidelines.xhtml

The formal assessment of eligibility and informed consent may only be obtained by the Principal Investigator (PI) or an appropriate medically qualified doctor. Patients will be screened from clinic lists. Disease eligibility will be based on the diagnostic MRI scan where recurrence is identified. For patients who are randomised to re-irradiation, if significant progression is observed on the re-irradiation planning imaging (with GTV volume >75cm3 or doubling compared to the baseline MRI), such cases will be reviewed with the RTTQA group before proceeding with re-irradiation. Patients who are randomised to chemotherapy who remain clinically stable would not routinely receive updated imaging prior to commencement of chemotherapy but in these cases the interval to starting chemotherapy is anticipated to be shorter than that to starting re-irradiation, providing less time for significant progression.

Table 1– Summary of key inclusion and exclusion criteria

**Inclusion criteria**	**Exclusion criteria**
Histologically proven diagnosis of GBM, based on original pathology.	Pregnant (positive pregnancy test) or lactating
First recurrence of GBM, with contrast enhancing disease, following primary treatment	Critical normal brain structures treated above usual tolerance during initial radiotherapy
Agreement of a Consultant Neuro-Radiologist that imaging changes are in keeping with recurrence and not pseudo-progression.	Recurrence with leptomeningeal disease or only leptomeningeal disease
≥6 months since completion of primary radiotherapy	More than three enhancing lesions present on MRI or multi-focal recurrence
Prior history of standard dose, conventionally fractionated CNS radiotherapy	IDH1/2 mutant tumours on original pathology
As a minimum, patients will have completed at least two weeks of temozolomide, concurrent with their original radiotherapy.	GBM with known features of PXA, BRAF mutations or 1p19q co-deletion (on original pathology or updated pathology if available)
Up to and including three enhancing lesions (size and volume criteria included in Supplementary Material)	Prior invasive malignancy (except non-melanomatous skin cancer), unless disease free for a minimum of one year.
Karnofsky Performance Status 70+	Severe active co-morbidity making patient unsuitable for chemotherapy or re-irradiation
Adequate hematologic, renal, and hepatic function	Prior allergic reaction to nitrosoureas
No contra-indication to lomustine	Any recognised genetic syndromes causing sensitivity to radiotherapy

For peer review only - http://bmjopen.bmj.com/site/about/guidelines.xhtml

Patients must be able to provide study-specific informed consent.	Contra-indication to MRI or gadolinium
Age 18 or over.	Previous radiotherapy dose distribution unavailable
	Previous systemic therapy or re-irradiation for recurrent GBM

Randomisation and recruitment

Randomisation will be performed centrally using University of Leeds Clinical Trials Research Unit (CTRU) automated 24-hour randomisation system. A computer-generated minimisation program incorporating a random element will be used to ensure the treatment groups are well balanced for the following factors:

- Age (<50 or ≥50 years)
- Time to randomisation from completion of previous radiotherapy (≤12 months or >12 months)
- MGMT promoter methylation status (Methylated, Unmethylated or Unknown)
- Randomising site

The intended recruitment period is 2-years (BRIOChe opened to recruitment on 22/06/2021).

Sample size

Thirty-three patients are required in the re-irradiation arm, 35 allowing for 5% drop out. With a 1:1 randomisation allocation ratio to include a calibration arm, the original sample size was 70 patients in total. As a result of slow recruitment, the randomisation allocation ratio was adapted in a protocol amendment to 2:1 (re-irradiation: chemotherapy), to allow a higher proportion of recruited patients into the intervention arm. The subsequent sample size was estimated to be 55 patients in total, with 35 in the re-irradiation arm and approximately 20 in chemotherapy arm.

For peer review only

For peer review only - http://bmjopen.bmj.com/site/about/guidelines.xhtml

This study is powered to demonstrate that the treatment strategy of re-irradiation demonstrates sufficient efficacy to warrant further large-scale evaluation, based on 9-month survival rates.

A Sargent's three-outcome, phase II, single-stage, single-arm design will be used to determine whether re-irradiation demonstrates sufficient efficacy to warrant further evaluation [33]. The trial is designed to test the null hypothesis (H0) that the proportion of participants alive at 9 months is <25% where re-irradiation would not be deemed worthy of further investigation, against an alternative hypothesis H1 of >45% where re-irradiation would be deemed worthy of further investigation. If the proportion of participants alive at 9 months is ≥25% and ≤45%, this is an inconclusive region where neither the null or alternative hypothesis would be rejected and the decision regarding further investigation would be based on other factors. The following operating characteristics are used: type I error=0.05, type II error=0.06, prob (correctly rejecting the H1) = 0.82 (eta), prob (correctly rejecting the H0) =0.79 (pi).

Treatment regimen

Re-irradiation arm: The radiotherapy prescription dose will be 35 Gy in 10 fractions, a commonly employed schedule [27-32], delivered daily Monday-Friday over two weeks. Treatment will be delivered with Intensity Modulated Radiotherapy (IMRT)/ Volumetric Modulated Arc Therapy (VMAT) or tomotherapy. Optimally, normal tissue constraints will be based on a 'dose remaining' approach, whereby an original organ at risk (OAR) dose (allowing for 25% repair) is subtracted from a cumulative tolerance to give a 'dose remaining' for re-irradiation. To save clinicians time and reduce errors in performing such calculations, the radiotherapy guidelines contain tables of 'OAR doses remaining' for ease of reference. Where these optimal constraints cannot be achieved, mandatory constraints are those used in the RTOG1205 trial, which also delivered a dose of 35 Gy in 10 fractions [31]. Treatment

For peer review only

For peer review only - http://bmjopen.bmj.com/site/about/guidelines.xhtml

compliance data to radiotherapy, including fractions, doses, interruptions and reasons will be collected weekly during treatment.

Chemotherapy arm: As per usual UK practice in the setting of relapsed GBM where the interval to recurrence is insufficiently long to use re-challenge temozolomide and/or when patients have O^6-methylguanine-DNA-methyltransferase (MGMT) unmethylated tumours, chemotherapy will be lomustine-based. These drugs have been used in this setting for many years. A variety of lomustine-based regimens are used across the UK including single agent lomustine, PCV (procarbazine, lomustine and vincristine), PC (procarbazine and lomustine) or CV (lomustine and vincristine). Lomustine-based regimens will be delivered as per each centre's standard practice. Doses will be recorded at each cycle and (based on a survey of participating centres regarding their usual practice) it is recommend that these will consist of:

- Lomustine 100-130mg/m^2, day 1, PO, on a 42 day cycle

  +/-
- Procarbazine 50-100mg/m^2, once daily on days 1-10 or days 2-11, PO, on a 42 day cycle

  +/-
- Vincristine 1.4-1.5mg/m^2, with dose capping as per the institution's usual practice, IV day 1, on a 42 day cycle.

(Dose capping is as per the institution's usual practice).

Treatment compliance data to chemotherapy will be collected from patient diaries. Dose modifications and reasons for changes will also be collected. Suggested dose modifications to chemotherapy based on haematological, renal and hepatic functioning are provided in the study protocol.

Upon further disease progression, patients may receive the alternative treatment according to local policy.

For peer review only

Trial assessments and follow-up

Full eligibility criteria (Table 1, Supplementary Material), will be assessed and confirmed prior to randomisation. Further assessments will be conducted at multiple timepoints. An overview of data collection can be seen in Table 2. Data will be collected from all participants, until progression or death. Where available, date of death will be collected for all participants. A final data sweep prior to the final analysis will collect information regarding death and any additional anti-cancer treatment received beyond progression.

Follow-up imaging with MRI will include T2, FLAIR (fluid attenuated inversion recovery), DWI (diffusion weighted imaging), T1 pre-gadolinium and T1 post-gadolinium sequences. In addition, patients randomised to re-irradiation will also undergo T2* Dynamic Susceptibility Contrast (DSC) perfusion as part of follow-up imaging to assist in determination of the presence of radionecrosis.

For peer review only

Table 2 – Trial assessment schedules

	Early assessments				Follow-up assessment (measured post start of treatment)									
	Eligibility	Baseline	Pre-treat RT	Pre-treat chemo	0w	1w	6w	12w	18w	24w	30w	36w	42w	48w
Informed consent	X													
Clinical assessment	X				X	X***	X	X	X	X	X	X	X	X
Performance status (Karnofsky)	X				X	X***	X	X	X	X	X	X	X	X
Bloods*	X			X	X	X	X	X	X	X	X			
Pregnancy screening**	X		X		X	X	X	X	X	X	X			
Re-irradiation					X	X								
Chemotherapy					X		X	X	X	X	X			
MRI (RANO criteria)	X						X			X				
Toxicity (CTCAE)		X			X	X***	X	X	X	X	X	X	X	X
HRQoL		X					X	X	X	X	X	X	X	X

* Bloods only for Eligibility for re-irradiation arm, with each cycle in chemotherapy arm

**only at pretreatment for re-irradiation arm, with every cycle in chemotherapy arm

*** re-irradiation arm patients only

Adverse Events (AEs), Serious Adverse Events (SAEs), Adverse Reaction (ARs), Serious Adverse Reactions (SARs) and Suspected Unexpected Serious Adverse Reactions (SUSARs) will all be collected from randomisation until the last participant follow-up or disease progression. Beyond this period only, SARs and SUSARs related to the trial treatment (and not GBM progression) will be reported if the investigator becomes aware of them until the end of trial notification. All SAEs/SARs and SUSARs related to investigation medicinal products (IMPs) will be reported to the Medicines and Healthcare products Regulatory Agency (MHRA) and sponsor. All radiotherapy related SAEs/SARs and SUSARs will be reported to the Research Ethics Committee and sponsor.

For peer review only

Statistical analysis

The statistical analysis will be conducted by University of Leeds CTRU. A full statistical analysis plan has been developed and finalised.

The trial analysis will be conducted on a Modified Intention to Treat (MITT) population [34]. MITT is defined as all participants randomised that have received at least one dose of trial treatment, analysed according to the treatment arm they were randomised to (regardless of ineligibility, non-compliance or withdrawal).

Overall survival rates at 9 months, i.e. the number and proportion of participants alive at 9 months post-start of treatment, will be calculated for the re-irradiation arm with 90% (corresponding to a 1-sided 5% significance level used in the design) and 95% confidence intervals (CIs).

Based on the 3-outcome design, the cut-off values and conclusions for the statistical test for the primary analysis of efficacy are defined as follows:

Green: If ≥13/33 patients are alive, re-irradiation demonstrates sufficient efficacy to warrant larger-scale evaluation. This is based on a 9-month OS >45% (i.e. competitive with the 'best' outcomes from prospective studies using nitrosourea-based chemotherapy [11, 14, 35].

Amber: If 11-12/33 patients are alive the decision to take re-irradiation forward will be uncertain and will be based on HRQoL and toxicity at 3 months, (this is based on 9-month OS 25%-45%; i.e. similar to outcomes from other prospective nitrosourea-based trials [13, 16, 17, 36].

Red: If ≤10/33 patients are alive re-irradiation will not be considered worthy of further investigation, based on 9-month OS <25%.

For peer review only

For peer review only - http://bmjopen.bmj.com/site/about/guidelines.xhtml

The chemotherapy arm is included for calibration and will be used for validation of outcomes in the re-irradiation group with respect to previous series, and validation of the assumptions used to inform the statistical cut-offs.

Summary statistics will be calculated for all domains of the EORTC QLQ-C30 and QLQ-BN20 and the overall summary score calculated, presented overall and by treatment arm for each of the follow-up time points. The difference in scores between treatment arms will be presented with corresponding 90% and 95% confidence intervals.

Summary statistics will be presented for dexamethasone requirement, anti-epileptic drug requirement, treatment compliance, and response assessment; including the proportion of participants within each clinical response status. The maximum CTCAE grade of toxicities experienced by participants will be summarised for each CTCAE term reported, by treatment arm, for the acute and late toxicities.

The assessment of PFS and OS will be based on a time to event analysis and presented using Kaplan Meier curves. Median PFS and OS will be presented with 95% CIs for each arms. There will be no formal comparison between arms, however, the unadjusted OS hazard ratio (HR) for the re-irradiation arm vs the chemotherapy arm will be presented.

Qualitative interviews

Qualitative exploration of HRQoL associated with brain tumour treatments will allow more in-depth evaluation of what matters most to patients and informal caregivers at the time of GBM recurrence; their perception of HRQoL before and during trial treatment; and how they experience both the treatment they receive and trial participation. Interviews will be recorded, transcribed, and thematically analysed [37]. A sub-sample of trial participants will be asked to partake in this interview, around 15 patients and their informal caregivers. We will utilise a maximum variation sample, to choose patients (and their informal caregivers) that differ in age, sex and previous/current treatment(s)

For peer review only

received. By using a varied sample, we aim to highlight important shared patterns that cut across cases and also discover unique or diverse variations from different participants [38].

Trial organisation

University of Leeds CTRU will manage all trial and data co-ordination according to the unit's well established standard operating procedures. All trial organisation will be conducted in-line with the principles of Good Clinical Practice.

Data collection and management

The BRIOChe trial data collection has been designed for remote data entry. However, all safety data will be recorded on paper case report forms and Patient Reported Outcome Measures (PROMs) on the relevant paper questionnaires, sent to and entered at CTRU. Participating centres will record and complete patient data on a trial specific database. The database has instantaneous validation checking. In addition, data management validation reports for missing and discrepant data.

All trial data is stored confidentially in a secure location at University of Leeds CTRU. Access to trial data will be restricted to CTRU staff and study team only, prior to the analysis and release of the trial results. Upon completion of the trial and publishing of results, researchers can request access to data by contacting members of the BRIOChe trial management group (TMG) or CTRU. All data will be stored and archived for a minimum of 25 years after trial completion.

For peer review only

Quality assurance

The radiotherapy quality assurance (QA) programme will be implemented by the National Cancer Research Institute (NCRI) Radiotherapy Trials Quality Assurance (RTTQA) group to ensure treatment is planned and delivered according to the trial protocol. RTTQA includes both benchmarking contouring and planning cases. A summary of the RTTQA requirements are provided in the BRIOChe radiotherapy outlining, planning, treatment delivery and QA guidelines. All sites must complete essential documentation and the site initiation process before a site can be activated. The site initiation will be an electronic process including an audio-visual recording. The video recorded site initiation presentation slides can be used for future training and reference at participating sites.

Trial monitoring

The BRIOChe trial will be monitored by the specific project delivery group at CTRU and the multi-disciplinary TMG. An independent data monitoring and ethics committee (DMEC) will be responsible for the safety and integrity of the patients and study, monitoring unblinded interim data. The DMEC will be formed of two independent clinical oncologists and one independent statistician, meeting at least annually, in addition to receiving 6-monthly safety reports. The DMEC members will advise the Trial Steering Committee (TSC) on any trial or safety concerns. The TSC have definitive control over the continuation of the trial. A DMEC charter has been developed including the roles and responsibilities, and communication processes.

Patient and Public Involvement

Patient and Public Involvement (PPI) has been an instrumental part of the development and conduct of the BRIOChe trial. The trial design was discussed with and presented to

For peer review only

PPI representatives. The trial question was felt to be of importance and the treatments and assessment schedule were considered acceptable.

The Trial Management Group includes a key patient representative, who provides ongoing and regular advice regarding trial conduct from the patient perspective.

Imaging sub-study

An imaging sub-study is planned between two of the recruiting centres (Leeds Teaching Hospitals NHS Trust and University College London Hospitals NHS Foundation Trust), to investigate the impact of re-irradiation and chemotherapy treatments on cerebral vascular changes and to improve understanding as to how these changes might be associated with both tumour response and normal tissue damage. The imaging sub-study is estimated to recruit 12-16 patients from the full BRIOChe trial. Sub-study participants who have been randomised to re-irradiation will have Arterial Spin Labelling (ASL) and T2 Dynamic Susceptibility Contrast (DSC) sequences added to the dedicated radiotherapy planning MRI. In addition, these participants will have ASL added to their routine follow up MRI scans (T2 DSC already included), to provide 'before' and 'after' re-irradiation information. Those sub-study participants randomised to chemotherapy will also have ASL and T2 DSC sequences added to their routine follow-up MRI to allow comparison between chemotherapy-treated and re-irradiated participants.

For peer review only

ETHICS AND DISSEMINATION

The trial obtained ethical approval from the Office for Research Ethics Committees Northern Ireland (ORECNI) (reference no. 20/NI/0070) and is registered in the ISRCTN registry (registration number 16052954). The trial is currently adhering to protocol version 4.0 (7/12/2022). Any protocol amendments will be submitted to MHRA and REC, all changes will be communicated with local sites.

There is no formal interim analysis. The final trial data will be analysed and reported approximately one year after the final participant is recruited. The final trial result manuscript will be written in accordance to ICMJE guidelines and submitted for publication to peer reviewed journals.

DISCUSSION

Despite the poor outcomes and high clinical need, there is a lack of active clinical trials for patients with recurrent GBM in the UK and no gold standard treatment at recurrence exists. Chemotherapy is often used in the UK, but outcomes are disappointing. Re-irradiation is recognised as an alternative treatment option and is listed as such in European and American treatment guidelines for patients with recurrent GBM, and in the current NICE guidelines for brain tumours [39-41]. Despite this, re-irradiation is less commonly utilised in the UK compared to other parts of the world. Re-irradiation offers a shorter course of treatment compared to chemotherapy and has a different toxicity profile, and so may be preferable for some patients, although the risks and benefits need further clarification. The BRIOChe trial aims to be a pragmatic, clinically relevant trial that evaluates re-irradiation as an alternative treatment option to chemotherapy for patients with recurrent GBM. While OS is the primary end point, HRQoL is a key secondary endpoint.

For peer review only

For peer review only - http://bmjopen.bmj.com/site/about/guidelines.xhtml

The trial design and treatment, specified in BRIOChe, intentionally reflects routine practice. Centres are able to use their usual lomustine-based chemotherapy regimen(s) thus reflecting the variety in practice across the UK. The frequently used and relatively well-tolerated hypofractionated schedule, 35Gy in 10 fractions, has been adopted for BRIOChe re-irradiation regimen [27-30] .

No organ at risk (OAR) constraints have been formally validated for GBM re-irradiation. Most GBM recurrences occur within the previous high dose region and so it is often necessary to assume that a degree of OAR recovery has occurred following the original course of radiotherapy if a meaningful dose is to be delivered to the re-irradiation target. As such, BRIOChe provides ‘radiobiological’ optimal constraints, allowing for 25% recovery from the original radiotherapy, based on limited evidence regarding post-irradiation recovery in neural structures [42-44]. In addition, where optimal constraints limit re-irradiation delivery, the more lenient mandatory constraints have been based on the RTOG-1205 trial [45]. Encouragingly since the opening of the BRIOChe trial, results from the RTOG1205 trial have been published indicating that re-irradiation, 35Gy in 10 fractions, as used in BRIOChe, in combination with bevacizumab, results in a median overall survival of 10.1 months [25]. In addition, re-irradiation was well tolerated [25]. However, re-irradiation alone was not investigated in RTOG1205, BRIOChe will investigate this further.

For peer review only

The eligibility criteria volume requirements consider whether the lesion is single or multi-focal. Size and volume limitations were included to limit the amount of normal brain re-irradiation in an effort to keep the risk of radionecrosis low. This approach was preferred over using 'diameter only' measurement as this could potentially exclude long but narrow recurrences, where the total volume was low.

For peer review only - http://bmjopen.bmj.com/site/about/guidelines.xhtml

The minimal acceptable interval between completion of original radiotherapy and commencement of re-irradiation is also unknown and this interval varies amongst existing studies. We have adopted a minimum interval of 6 months from completion of first radiotherapy in BRIOChe, which is in-keeping with current practices in other countries [46] and international trials using the same re-irradiation schedule [31, 32]. An interval of at least 6 months also allows some time for OAR repair, should repair occur, and reduces the risk of mistaking pseudo-progression for true progression, which typically occurs within 3 months of radiotherapy [47].

It is acknowledged as a limitation that the trial design and decision criteria are based on available historical data at the time of development. The referenced studies of nitrosourea-based chemotherapy required intervals between recurrence to treatment that are shorter than the 6 months required in order to enter BRIOChe, potentially selecting some patients that come from a slightly better prognosis patient group. The trial has taken a pragmatic approach given the paucity of data with the exact criteria for those treated with systemic therapy.

While the patient numbers involved will not permit a direct comparison of survival outcomes in each arm, the BRIOChe trial aims to demonstrate that re-irradiation is an acceptable alternative treatment option for patients with recurrent GBM, including from the patient perspective. This in turn will allow the development of future novel approaches, including potential treatment intensification strategies using re-irradiation in combination with novel agents such as immunotherapies and radiosensitisers, to be explored in large-scale platform studies.

For peer review only

For peer review only - http://bmjopen.bmj.com/site/about/guidelines.xhtml

513 The BRIOChe study will provide valuable data for patients with recurrent GBM treated with re-irradiation and nitrosourea-based chemotherapy, forming a first step in improving treatment for this poor prognosis patient group.

For peer review only

AUTHOR CONTRIBUTIONS

Contributors to the conception, trial design and ongoing conduct: LM, FB, SCS, EH, SN, JW, SB, OA, HBe, HBu, SC, SF, JH, JL, AS, CP, FS, DSM.

Development of the protocol, trial documents and patient information sheet: EH, LM, SB, SN, FB, CP, SC, JL.

Writing manuscript: EH, LM.

Reviewed manuscript: all authors

All authors read and approved the final manuscript.

FUNDING, SPONSOR AND COORDINATION

BRIOChe is supported by The Jon Moulton Charity Trust (NA), and by Yorkshire Cancer Research (YCR), Centre for Early Phase Clinical Trials (L375PA). The imaging sub-study is supported by Cancer Research UK funding for the Leeds Radiotherapy Research Centre of Excellence (RadNet; C19942/A28832).

The study is coordinated by University of Leeds CTRU and sponsored by University of Leeds (GA18/118305). For enquires, trial contact *email:* BRIOCHE@leeds.ac.uk. The sponsor and funders have had no influence on the BRIOChe trial study design, collection, management, analysis, and interpretation of data; and decision for publication. The sponsor and funder will review all manuscripts prior to publication.

COMPETING INTERESTS

FS reports grants from Cancer Research UK, during the conduct of the study. DSM reports grants from Jon Moulton Charitable Foundation, grants from Yorkshire Cancer Research,

For peer review only

during the conduct of the study. LM reports grants from Jon Moulton Charity Trust, grants from Cancer Research UK, during the conduct of the study; grants from Yorkshire Cancer Research, outside the submitted work. SB reports grants from Jon Moulton Charitable Foundation, grants from Yorkshire Cancer Research, during the conduct of the study. SS reports advisory board activity for Bayer, CeCaVa Laboratory Research project support, Apollomics.

EXCLUSIVE LICENCE

I, the Submitting Author has the right to grant and does grant on behalf of all authors of the Work (as defined in the below author licence), an exclusive licence and/or a non-exclusive licence for contributions from authors who are: i) UK Crown employees; ii) where BMJ has agreed a CC-BY licence shall apply, and/or iii) in accordance with the terms applicable for US Federal Government officers or employees acting as part of their official duties; on a worldwide, perpetual, irrevocable, royalty-free basis to BMJ Publishing Group Ltd (“BMJ”) its licensees and where the relevant Journal is co-owned by BMJ to the co-owners of the Journal, to publish the Work in BMJ Open and any other BMJ products and to exploit all rights, as set out in our licence.

The Submitting Author accepts and understands that any supply made under these terms is made by BMJ to the Submitting Author unless you are acting as an employee on behalf of your employer or a postgraduate student of an affiliated institution which is paying any applicable article publishing charge (“APC”) for Open Access articles. Where the Submitting Author wishes to make the Work available on an Open Access basis (and intends to pay the relevant APC), the terms of reuse of such Open Access shall be governed by a Creative

For peer review only

Commons licence – details of these licences and which Creative Commons licence will apply to this Work are set out in our licence referred to above.

FIGURE LEGEND: Figure 1 - BRIOChe Trial schema

For peer review only

REFERENCES

1. Stupp, R., et al., *Radiotherapy plus concomitant and adjuvant temozolomide for glioblastoma.* New England journal of medicine, 2005. **352**(10): p. 987-996.
2. Gately, L., et al., *Life beyond a diagnosis of glioblastoma: a systematic review of the literature.* Journal of Cancer Survivorship, 2017. **11**(4): p. 447-452.
3. Mummudi, N. and R. Jalali, *Palliative care and quality of life in neuro-oncology.* F1000prime reports, 2014. **6**.
4. Arvold, N.D., et al., *Hospitalization burden and survival among older glioblastoma patients.* Neuro-oncology, 2014. **16**(11): p. 1530-1540.
5. Mukand, J.A., et al., *Incidence of neurologic deficits and rehabilitation of patients with brain tumors.* American journal of physical medicine & rehabilitation, 2001. **80**(5): p. 346-350.
6. Boele, F.W., et al., *Symptom management and quality of life in glioma patients.* CNS oncology, 2014. **3**(1): p. 37-47.
7. Sterckx, W., et al., *The impact of a high-grade glioma on everyday life: a systematic review from the patient's and caregiver's perspective.* European Journal of Oncology Nursing, 2013. **17**(1): p. 107-117.
8. Weller, M., et al., *Standards of care for treatment of recurrent glioblastoma—are we there yet?* Neuro-oncology, 2013. **15**(1): p. 4-27.
9. van Linde, M.E., et al., *Treatment outcome of patients with recurrent glioblastoma multiforme: a retrospective multicenter analysis.* Journal of neuro-oncology, 2017. **135**(1): p. 183-192.
10. Das, P., S. McCaffrey, and D. Conkey, *Re-evaluating the efficacy and toxicity of procarbazine, lomustine and vincrsitine (PCV) in relapsed glioblastoma in the concurrent/ adjuvant temozolamide era.* Journal of Cancer Prevention and Current Research, 2016. **6**(3): p. 1-6.
11. Taal, W., et al., *Single-agent bevacizumab or lomustine versus a combination of bevacizumab plus lomustine in patients with recurrent glioblastoma (BELOB trial): a randomised controlled phase 2 trial.* The lancet oncology, 2014. **15**(9): p. 943-953.
12. Carvalho, B.F., et al., *Second-line chemotherapy in recurrent glioblastoma: a 2-cohort study.* Oncology research and treatment, 2015. **38**(7-8): p. 348-354.
13. Brada, M., et al., *Temozolomide versus procarbazine, lomustine, and vincristine in recurrent high-grade glioma.* J Clin Oncol, 2010. **28**(30): p. 4601-4608.
14. Wick, W., et al., *Lomustine and bevacizumab in progressive glioblastoma.* New England Journal of Medicine, 2017. **377**(20): p. 1954-1963.
15. Rajan, B., et al., *Survival in patients with recurrent glioma as a measure of treatment efficacy: prognostic factors following nitrosourea chemotherapy.* European Journal of Cancer, 1994. **30**(12): p. 1809-1815.
16. Wick, W., et al., *Phase III study of enzastaurin compared with lomustine in the treatment of recurrent intracranial glioblastoma.* Journal of clinical oncology, 2010. **28**(7): p. 1168.
17. Lombardi, G., et al. *Updated results of REGOMA: A randomized, multicenter, controlled open-label phase II clinical trial evaluating regorafenib in relapsed glioblastoma (GBM) patients (PTS)*. in *Oral Presentation at EANO (Eurpoean Association of Neuro-Oncolgy) Annual conference*. 2018.
18. Dirven, L., et al., *Dutch Neuro-Oncology Group (LWNO). The impact of bevacizumab on health-related quality of life in patients treated for recurrent glioblastoma: results of the randomised controlled phase 2 BELOB trial.* Eur J Cancer, 2015. **51**(10): p. 1321-1330.
19. Pessina, F., et al., *Role of surgical resection in recurrent glioblastoma: prognostic factors and outcome evaluation in an observational study.* Journal of neuro-oncology, 2017. **131**(2): p. 377-384.
20. Franceschi, E., et al., *The effect of re-operation on survival in patients with recurrent glioblastoma.* Anticancer research, 2015. **35**(3): p. 1743-1748.

For peer review only - http://bmjopen.bmj.com/site/about/guidelines.xhtml

21. Gorlia, T., et al., *New prognostic factors and calculators for outcome prediction in patients with recurrent glioblastoma: a pooled analysis of EORTC Brain Tumour Group phase I and II clinical trials.* European journal of cancer, 2012. **48**(8): p. 1176-1184.
22. Kazmi, F., et al., *Re-irradiation for recurrent glioblastoma (GBM): a systematic review and meta-analysis.* Journal of neuro-oncology, 2019. **142**(1): p. 79-90.
23. Shanker, M., et al., *Re-irradiation for recurrent high-grade gliomas: a systematic review and analysis of treatment technique with respect to survival and risk of radionecrosis.* Neuro-Oncology Practice, 2019. **6**(2): p. 144-155.
24. Nieder, C., et al., *Improvement, clinical course, and quality of life after palliative radiotherapy for recurrent glioblastoma.* American journal of clinical oncology, 2008. **31**(3): p. 300-305.
25. Tsien, C.I., et al., *NRG Oncology/RTOG1205: a randomized phase II trial of concurrent bevacizumab and reirradiation versus bevacizumab alone as treatment for recurrent glioblastoma.* Journal of clinical oncology, 2023. **41**(6): p. 1285-1295.
26. Grant, R., et al., *OP01THE UK TOP 10 CLINICAL RESEARCH PRIORITIES IN NEURO-ONCOLOGY.* Neuro-Oncology, 2015. **17**(Suppl 8): p. viii16.
27. Klobukowski, L., et al., *A retrospective review of re-irradiating patients' recurrent high-grade gliomas.* Clinical Oncology, 2018. **30**(9): p. 563-570.
28. Fogh, S.E., et al., *Hypofractionated stereotactic radiation therapy: an effective therapy for recurrent high-grade gliomas.* Journal of clinical oncology, 2010. **28**(18): p. 3048.
29. Hudes, R.S., et al., *A phase I dose escalation study of hypofractionated stereotactic radiotherapy as salvage therapy for persistent or recurrent malignant glioma.* International Journal of Radiation Oncology* Biology* Physics, 1999. **43**(2): p. 293-298.
30. Tsien, C., et al., *ACTR-32. NRG ONCOLOGY RTOG 1205: randomized phase II trial of concurrent bevacizumab and re-irradiation vs. bevacizumab alone as treatment for recurrent glioblastoma.* Neuro-Oncology, 2019. **21**(Suppl 6): p. vi20.
31. RTOG 1205. *Randomized phase II trial of concurrent bevacizumab and re-irradiation versus bevacizumab alone as treatment of recurrent glioblastoma.* 2016; Available from: https://clinicaltrials.gov/ct2/show/NCT01730950.
32. AstraZeneca, *AZD1390 Radiation Procedure Manual. Version 1.0.* 2017.
33. Sargent, D.J., V. Chan, and R.M. Goldberg, *A three-outcome design for phase II clinical trials.* Controlled clinical trials, 2001. **22**(2): p. 117-125.
34. Abraha, I. and A. Montedori, *Modified intention to treat reporting in randomised controlled trials: systematic review.* Bmj, 2010. **340**.
35. Batchelor, T.T., et al., *Phase III randomized trial comparing the efficacy of cediranib as monotherapy, and in combination with lomustine, versus lomustine alone in patients with recurrent glioblastoma.* Journal of Clinical Oncology, 2013. **31**(26): p. 3212.
36. Boiardi, A., et al., *Efficacy of '8-drugs-in-one-day'combination in treatment of recurrent GBM patients.* Journal of neuro-oncology, 1992. **12**(2): p. 153-158.
37. Fereday, J. and E. Muir-Cochrane, *Demonstrating rigor using thematic analysis: A hybrid approach of inductive and deductive coding and theme development.* International journal of qualitative methods, 2006. **5**(1): p. 80-92.
38. Patton, M., *Pattern, theme, and content analysis*, in *Qualitative research & evaluation methods*. 2002, Sage Publications Ltd, London. p. 452-471.
39. Cabrera, A.R., et al., *Radiation therapy for glioblastoma: executive summary of an American Society for Radiation Oncology evidence-based clinical practice guideline.* Practical radiation oncology, 2016. **6**(4): p. 217-225.
40. Stupp, R., et al., *High-grade glioma: ESMO Clinical Practice Guidelines for diagnosis, treatment and follow-up.* Annals of Oncology, 2014. **25**: p. iii93-iii101.
41. NICE, N.I.f.H.a.C.E., *Brain tumours (primary) and brain metastases in adults.* NICE guideline [NG99], 2018.
42. Kirkpatrick, J.P., A.J. van der Kogel, and T.E. Schultheiss, *Radiation dose–volume effects in the spinal cord.* International Journal of Radiation Oncology* Biology* Physics, 2010. **76**(3): p. S42-S49.

For peer review only - http://bmjopen.bmj.com/site/about/guidelines.xhtml

43. Ang, K.K., et al., *Extent and kinetics of recovery of occult spinal cord injury.* International Journal of Radiation Oncology* Biology* Physics, 2001. **50**(4): p. 1013-1020.
44. Flickinger, J.C., M. Deutsch, and L.D. Lunsford, *Repeat megavoltage irradiation of pituitary and suprasellar tumors.* International Journal of Radiation Oncology* Biology* Physics, 1989. **17**(1): p. 171-175.
45. Tsien, C., et al., *Randomized phase II trial of re-irradiation and concurrent bevacizumab versus bevacizumab alone as treatment for recurrent glioblastoma (NRG Oncology/RTOG 1205): initial outcomes and RT plan quality report.* International Journal of Radiation Oncology Biology and Physics, 2019. **105**(1).
46. Post, C.C., et al., *Patterns of re-irradiation for recurrent gliomas and validation of a prognostic score.* Radiotherapy and Oncology, 2019. **130**: p. 156-163.
47. De Wit, M., et al., *Immediate post-radiotherapy changes in malignant glioma can mimic tumor progression.* Neurology, 2004. **63**(3): p. 535-537.

For peer review only

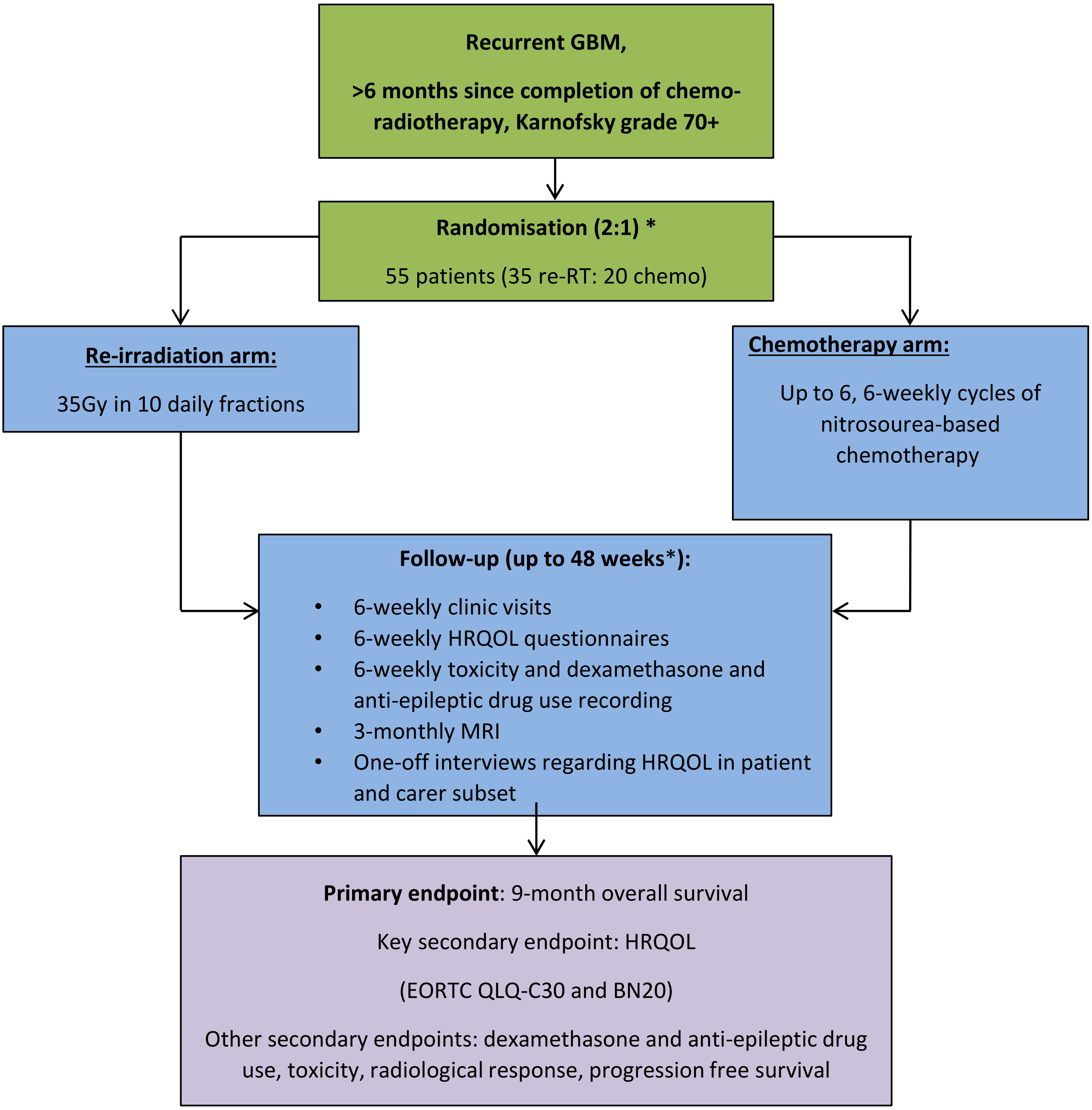

**allocation ratio originally 1:1, ratio was adapted to 2:1 during the recruitment phase*

EORTC QLQ-C30 and BN20, European Organization for Research and Treatment of Cancer Quality of life questionnaire core 30 and Brain Cancer module 20; GBM, glioblastoma multiforme; HRQoL, health-related quality of life; MRI, magnetic resonance imaging;

SUPPLEMENTARY FILE 1 – Full inclusion exclusion criteria

Inclusion criteria	Exclusion criteria
1. Histologically proven diagnosis of GBM with consistent molecular pathology, based on original pathology.	1. Pregnant (positive pregnancy test) or lactating
2. First recurrence of GBM, with contrast enhancing disease, following primary treatment (i.e. no previous systemic therapy or re-irradiation for recurrence).	2. Critical normal brain structures treated above usual tolerance during initial radiotherapy
3. Agreement of a Consultant Neuro-Radiologist within multi-disciplinary team meeting that imaging changes are in keeping with recurrence and not pseudo-progression.	3. Recurrence with leptomeningeal disease or only leptomeningeal disease
4. Randomisation must be performed within 21 days of the MRI that confirms recurrence.	4. Recurrence defined by non-enhancing disease only
5. ≥6 months since completion of primary radiotherapy	5. More than three enhancing lesions present on MRI or multi-focal recurrence
6. Prior history of standard dose, conventionally fractionated CNS radiotherapy	6. IDH1/2 mutant tumours on original pathology (to avoid unbalance between arms).
7. As a minimum patients will have completed at least two weeks of temozolomide, concurrent with their original radiotherapy.	7. GBM with known features of PXA, BRAF mutations or 1p19q co-deletion (on original pathology or updated pathology if available)
• Up to and including three enhancing lesions:<br>○ In cases of a single recurrent enhancing lesion:<br>▪ Predicted re-irradiation GTV<75cm^3 and<br>▪ Maximum diameter of enhancing disease must be ≤6cm.<br>○ In cases of multiple (i.e. two or three) discrete recurrent enhancing lesions:<br>▪ The total (i.e. combined) predicted re-irradiation GTV must be <50cm^3 and lesions must be clustered in a similar brain region such that PTVs are anticipated to be adjacent or overlapping and<br>▪ Maximum diameter of the combined enhancing disease, across all enhancing lesions (including any gaps between), must be ≤6cm.	8. Prior invasive malignancy (except non-melanomatous skin cancer), unless disease free for a minimum of one year.

For peer review only - http://bmjopen.bmj.com/site/about/guidelines.xhtml

8. Karnofsky Performance Status 70+	9. Severe active co-morbidity making patient unsuitable for chemotherapy or re-irradiation
9. Adequate hematologic, renal, and hepatic function	10. Prior allergic reaction to nitrosoureas
10. Patients who have had surgery for first recurrence may also be included provided there is residual enhancing disease on the immediate post-operative MRI or if enhancing disease develops on subsequent follow-up imaging, provided no prior systemic therapy or re-irradiation for recurrence has been given.	11. Any recognised genetic syndromes causing sensitivity to radiotherapy
11. Patients must have recovered from the prior effects of therapy	12. Patient unwilling/ unable to attend for follow up in the radiotherapy centre
12. Medical history and physical examination, including CNS examination, must be performed within 14 days prior to randomisation.	13. Contra-indication to MRI or gadolinium
13. Female participants of child-bearing potential must agree to be pregnancy screened.	14. Previous radiotherapy dose distribution unavailable
14. Patients must be able to swallow oral medication.	15. Previous systemic therapy or re-irradiation for recurrent GBM
15. Patients must be able to provide study-specific informed consent.	16. Spinal or infratentorial disease
16. Age 18 or over.	
17. Patients should be able to start treatment within 21 days of randomisation and must start within 28 days of randomisation.	

Coeliac disease is usually considered a contra-indication to lomustine as there is wheat within the capsule. For patients with recurrent GBM, the individual clinician may decide whether or not this is a relevant exclusion criteria.

For peer review only

Delete this line, then print on Trust/Hospital headed paper

Participant ID:	Initials:
Date of Birth:	NHS/Hospital Number:
EudraCT Number: 2019-004053-91	Principal Investigator:

BRIOChe

SAMPLE

PARTICIPANT CONSENT FORM

Please initial each box

1. I confirm that I have read and understood the information sheet for the above study and have had the opportunity to ask questions.

2. I understand that my participation in this study is voluntary and that I am free to withdraw at any time without my medical care or legal rights being affected. I understand that even if I withdraw from the above study, the data and samples collected from me will be used in analysing the results of the study and in some cases further information about any unwanted effects of my treatment may need to be collected by the study team.

3. I understand that my healthcare records may be looked at by authorised individuals from the study team, regulatory bodies or Sponsor in order to check that the study is being carried out correctly.

4. I agree to allow any information or results arising from this study to be used for healthcare and/or further medical research upon the understanding that my identity will remain anonymous wherever possible. This includes the use of my radiotherapy data and imaging data for future analysis.

5. I understand that if I lose capacity, further information about any unwanted effects of my treatment may need to be collected by the study team.

6. If selected, I agree to take part in a semi-structured interview on my experiences of participating in the trial and my health-related quality of life, where a member of the research team may approach me directly to schedule a suitable day and time. I agree to these interviews being audio recorded.

7. I agree to consent to my anonymised trial data to be viewed if required by 3rd parties outside the UK / EU etc.

8. I agree to a copy of this Consent Form being sent to the Clinical Trials Research Unit.

9. I agree that my GP, or any other doctor treating me, will be notified of my participation in this study.

10. I agree to take part in the study.

The following points are **OPTIONAL.**

Title: Consent for potential tissue collection and use in future research (if you do not wish to give this permission do not initial the boxes – you can still participate in the trial)

Please initial each box

I give my permission for stored tumour tissue sample that was removed during my operation and was not needed for routine diagnosis and treatment to be collected and used for future translational research purposes as described in the information sheet for the above trial if this becomes relevant in the future.

Yes **No**

I understand how the tissue sample will be collected, that giving samples is voluntary and that I'm free to withdraw my approval for use of the samples at any time without giving a reason and without my medical care or legal rights being affected.

Yes **No**

SAMPLE

For peer review only

Patient:
Signature…………………………………………………………………………………

Name (block capitals)………………………………………………………………….

Date…………………………………………………..…………………………………

Investigator:

I have explained the study to the above named patient and he/she has indicated his/her willingness to participate.

Signature………………………………………..………………………………………

Name (block capitals)…………………………………………………..……………….

Date………………………………………………………………………………………

(If used)Translator:

Signature…………………………………………………………………………………

Name (block capitals)…………………………………………………..……………….

Date………………………………………………………………………………………

(1 copy for patient; 1 for the CTRU; 1 held in patient notes;

SAMPLE

For peer review only

SPIRIT

STANDARD PROTOCOL ITEMS: RECOMMENDATIONS FOR INTERVENTIONAL TRIALS

SPIRIT 2013 Checklist: Recommended items to address in a clinical trial protocol and related documents*

Section/item	Item No	Description	Addressed on page number
**Administrative information**			
Title	1	Descriptive title identifying the study design, population, interventions, and, if applicable, trial acronym	1 ____________
Trial registration	2a	Trial identifier and registry name. If not yet registered, name of intended registry	4, 23___________
	2b	All items from the World Health Organization Trial Registration Data Set	Many___________
Protocol version	3	Date and version identifier	23 ____________
Funding	4	Sources and types of financial, material, and other support	4, 26___________
Roles and responsibilities	5a	Names, affiliations, and roles of protocol contributors	1, 26 __________
	5b	Name and contact information for the trial sponsor	26 ____________
	5c	Role of study sponsor and funders, if any, in study design; collection, management, analysis, and interpretation of data; writing of the report; and the decision to submit the report for publication, including whether they will have ultimate authority over any of these activities	26 ____________
	5d	Composition, roles, and responsibilities of the coordinating centre, steering committee, endpoint adjudication committee, data management team, and other individuals or groups overseeing the trial, if applicable (see Item 21a for data monitoring committee)	20, 21 _________

For peer review only

For peer review only - http://bmjopen.bmj.com/site/about/guidelines.xhtml

Introduction

Background and rationale	6a	Description of research question and justification for undertaking the trial, including summary of relevant studies (published and unpublished) examining benefits and harms for each intervention	6,7, 8_____
	6b	Explanation for choice of comparators	6, 7__________
Objectives	7	Specific objectives or hypotheses	11 _________
Trial design	8	Description of trial design including type of trial (e.g., parallel group, crossover, factorial, single group), allocation ratio, and framework (e.g., superiority, equivalence, non-inferiority, exploratory)	9 ___________

Methods: Participants, interventions, and outcomes

Study setting	9	Description of study settings (e.g., community clinic, academic hospital) and list of countries where data will be collected. Reference to where list of study sites can be obtained	9 ________
Eligibility criteria	10	Inclusion and exclusion criteria for participants. If applicable, eligibility criteria for study centres and individuals who will perform the interventions (e.g., surgeons, psychotherapists)	13, Table 1, Supplementary file 1
Interventions	11a	Interventions for each group with sufficient detail to allow replication, including how and when they will be administered	15__________
	11b	Criteria for discontinuing or modifying allocated interventions for a given trial participant (e.g., drug dose change in response to harms, participant request, or improving/worsening disease)	15, 16_________
	11c	Strategies to improve adherence to intervention protocols, and any procedures for monitoring adherence (e.g., drug tablet return, laboratory tests)	14,15 ________
	11d	Relevant concomitant care and interventions that are permitted or prohibited during the trial	N/A________
Outcomes	12	Primary, secondary, and other outcomes, including the specific measurement variable (e.g., systolic blood pressure), analysis metric (e.g., change from baseline, final value, time to event), method of aggregation (e.g., median, proportion), and time point for each outcome. Explanation of the clinical relevance of chosen efficacy and harm outcomes is strongly recommended	11 _____

For peer review only - http://bmjopen.bmj.com/site/about/guidelines.xhtml

Participant timeline	13	Time schedule of enrolment, interventions (including any run-ins and washouts), assessments, and visits for participants. A schematic diagram is highly recommended (see Figure)	Table 2, Figure 1
Sample size	14	Estimated number of participants needed to achieve study objectives and how it was determined, including clinical and statistical assumptions supporting any sample size calculations	14_______
Recruitment	15	Strategies for achieving adequate participant enrolment to reach target sample size	12______

Methods: Assignment of interventions (for controlled trials)

Allocation:

Sequence generation	16a	Method of generating the allocation sequence (e.g., computer-generated random numbers), and list of any factors for stratification. To reduce predictability of a random sequence, details of any planned restriction (eg, blocking) should be provided in a separate document that is unavailable to those who enrol participants or assign interventions	13, 14 ___________
Allocation concealment mechanism	16b	Mechanism of implementing the allocation sequence (e.g., central telephone; sequentially numbered, opaque, sealed envelopes), describing any steps to conceal the sequence until interventions are assigned	13, 14 ____________
Implementation	16c	Who will generate the allocation sequence, who will enrol participants, and who will assign participants to interventions	11, 12, 13______
Blinding (masking)	17a	Who will be blinded after assignment to interventions (e.g., trial participants, care providers, outcome assessors, data analysts), and how	N/A _________
	17b	If blinded, circumstances under which unblinding is permissible, and procedure for revealing a participant's allocated intervention during the trial	N/A__________

Methods: Data collection, management, and analysis

Data collection methods	18a	Plans for assessment and collection of outcome, baseline, and other trial data, including any related processes to promote data quality (e.g., duplicate measurements, training of assessors) and a description of study instruments (e.g., questionnaires, laboratory tests) along with their reliability and validity, if known. Reference to where data collection forms can be found, if not in the protocol	10, 11, (Figure 1)

For peer review only - http://bmjopen.bmj.com/site/about/guidelines.xhtml

	18b	Plans to promote participant retention and complete follow-up, including list of any outcome data to be collected for participants who discontinue or deviate from intervention protocols	16__________
Data management	19	Plans for data entry, coding, security, and storage, including any related processes to promote data quality (e.g., double data entry; range checks for data values). Reference to where details of data management procedures can be found, if not in the protocol	20 _________
Statistical methods	20a	Statistical methods for analysing primary and secondary outcomes. Reference to where other details of the statistical analysis plan can be found, if not in the protocol	18,19______
	20b	Methods for any additional analyses (e.g., subgroup and adjusted analyses)	18,19_______
	20c	Definition of analysis population relating to protocol non-adherence (e.g., as randomised analysis), and any statistical methods to handle missing data (e.g., multiple imputation)	18 _________
**Methods: Monitoring**			
Data monitoring	21a	Composition of data monitoring committee (DMC); summary of its role and reporting structure; statement of whether it is independent from the sponsor and competing interests; and reference to where further details about its charter can be found, if not in the protocol. Alternatively, an explanation of why a DMC is not needed	21 __________
	21b	Description of any interim analyses and stopping guidelines, including who will have access to these interim results and make the final decision to terminate the trial	21, 23 ________
Harms	22	Plans for collecting, assessing, reporting, and managing solicited and spontaneously reported adverse events and other unintended effects of trial interventions or trial conduct	17 ____
Auditing	23	Frequency and procedures for auditing trial conduct, if any, and whether the process will be independent from investigators and the sponsor	20_________
**Ethics and dissemination**			
Research ethics approval	24	Plans for seeking research ethics committee/institutional review board (REC/IRB) approval	23 ___________

Protocol amendments	25	Plans for communicating important protocol modifications (e.g., changes to eligibility criteria, outcomes, analyses) to relevant parties (e.g., investigators, REC/IRBs, trial participants, trial registries, journals, regulators)	23____________
Consent or assent	26a	Who will obtain informed consent or assent from potential trial participants or authorised surrogates, and how (see Item 32)	11 ___________
	26b	Additional consent provisions for collection and use of participant data and biological specimens in ancillary studies, if applicable	N/A__________
Confidentiality	27	How personal information about potential and enrolled participants will be collected, shared, and maintained in order to protect confidentiality before, during, and after the trial	20 ________
Declaration of interests	28	Financial and other competing interests for principal investigators for the overall trial and each study site	26 ___________
Access to data	29	Statement of who will have access to the final trial dataset, and disclosure of contractual agreements that limit such access for investigators	20 ________
Ancillary and post-trial care	30	Provisions, if any, for ancillary and post-trial care, and for compensation to those who suffer harm from trial participation	N/A ________
Dissemination policy	31a	Plans for investigators and sponsor to communicate trial results to participants, healthcare professionals, the public, and other relevant groups (e.g., via publication, reporting in results databases, or other data sharing arrangements), including any publication restrictions	23 _____
	31b	Authorship eligibility guidelines and any intended use of professional writers	23 __________
	31c	Plans, if any, for granting public access to the full protocol, participant-level dataset, and statistical code	20_________
**Appendices**			
Informed consent materials	32	Model consent form and other related documentation given to participants and authorised surrogates	Supplementary fie 2 _________
Biological specimens	33	Plans for collection, laboratory evaluation, and storage of biological specimens for genetic or molecular analysis in the current trial and for future use in ancillary studies, if applicable	_N/A__________

For peer review only - http://bmjopen.bmj.com/site/about/guidelines.xhtml

*It is strongly recommended that this checklist be read in conjunction with the SPIRIT 2013 Explanation & Elaboration for important clarification on the items. Amendments to the protocol should be tracked and dated. The SPIRIT checklist is copyrighted by the SPIRIT Group under the Creative Commons "Attribution-NonCommercial-NoDerivs 3.0 Unported" license.

For peer review only

BMJ Open

Brain Re-Irradiation Or Chemotherapy: a phase II randomised trial of re-irradiation and chemotherapy in patients with recurrent glioblastoma (BRIOChe): Protocol for a multi-centre open label randomised trial

Journal:	*BMJ Open*
Manuscript ID	bmjopen-2023-078926.R3
Article Type:	Protocol
Date Submitted by the Author:	14-Feb-2024
Complete List of Authors:	Hudson, Eleanor; University of Leeds, Leeds Institute of Clinical Trials Research<br>Noutch, Samantha; University of Leeds, Leeds Institute of Clinical Trials Research<br>Webster, Joanne; University of Leeds, Leeds Institute of Clinical Trials Research<br>Brown, Sarah; University of Leeds, Clinical Trials Research Unit<br>Boele, Florien; University of Leeds, Leeds Institute of Medical Research at St James's; University of Leeds, Leeds Institute of Health Sciences<br>Al-Salihi, Omar; Guy's and St Thomas' Hospitals NHS Trust<br>Baines, Helen; Mount Vernon Cancer Centre, National Radiotherapy Trials QA (RTTQA) Group<br>Bulbeck, Helen; brainstrust,<br>Currie, Stuart; Leeds General Infirmary, Department of Radiology<br>Fernandez, Sharon; University of Leeds, Leeds Institute of Medical Research at St James's<br>Hughes, Jane; University of Leeds, Leeds Institute of Medical Research at St James's<br>Lilley, John; Leeds Institute of Cancer and Pathology, Department of Medical Physics<br>Smith, Alexandra; University of Leeds, Leeds Institute of Clinical Trials Research<br>Parbutt, Catherine; Leeds Teaching Hospitals NHS Trust, Pharmacy<br>Slevin, Finbar; Leeds Institute of Cancer and Pathology, Department of Clinical Oncology; University of Leeds, Leeds Institute of Medical Research at St James's<br>Short, Susan; Leeds Institute of Cancer and Pathology, Department of Clinical Oncology; University of Leeds, Leeds Institute of Medical Research at St James's<br>Sebag-Montefiore, David ; University of Leeds Faculty of Medicine and Health, Institute of Cancer and Pathology<br>Murray, Louise; Leeds Institute of Cancer and Pathology, Department of Clinical Oncology; University of Leeds, Leeds Institute of Medical Research at St James's
**Primary Subject Heading**:	Oncology

Secondary Subject Heading:	Oncology
Keywords:	Radiation oncology < RADIOTHERAPY, RADIOTHERAPY, ONCOLOGY, Neurological oncology < NEUROLOGY

SCHOLARONE™ Manuscripts

Brain Re-Irradiation Or Chemotherapy: a phase II randomised trial of re-irradiation and chemotherapy in patients with recurrent glioblastoma (BRIOChe): Protocol for a multi-centre open label randomised trial

Authors:

Eleanor Hudson[1], Samantha Noutch[1], Joanne Webster[1], Sarah R. Brown[1], Florien W. Boele[2,3], Omar Al-Salihi[4], Helen Baines[5], Helen Bulbeck[6], Stuart Currie[7], Sharon Fernandez[2] Jane Hughes[2], John Lilley[8], Alexandra Smith[1], Catherine Parbutt[9], Finbar Slevin[2,10], Susan C. Short[2,10], David Sebag-Montefiore[2], Louise Murray[2,10]

Author affiliation:

[1]Clinical Trials Research Unit, Leeds Institute of Clinical Trials Research, University of Leeds, Leeds, United Kingdom

[2]Leeds Institute of Medical Research at St James's, University of Leeds, Leeds, United Kingdom

[3]Leeds Institute of Health Sciences, Faculty of Medicine and Health, University of Leeds, Leeds, UK

[4]Guy's and St Thomas Hospital, London, United Kingdom

[5]National Radiotherapy Trials QA (RTTQA) Group, United Kingdom

[6]Patient Advocate, United Kingdom

[7]Department of Radiology, Leeds General Infirmary, Leeds, United Kingdom

[8]Department of Medical Physics, Leeds Cancer Centre, Leeds, United Kingdom

[9]Pharmacy Department, St James's University Hospital, Leeds, United Kingdom

[10]Department of Clinical Oncology, Leeds Cancer Centre, Leeds, United Kingdom

For peer review only

Corresponding author: Dr. Louise Murray

L.J.Murray@leeds.ac.uk

Leeds Institute of Medical Research at St James's,

University of Leeds,

St James's University Hospital,

Harehills

Leeds,

LS9 7LP

+44 113 206 7854

Word count (excluding title page, abstract, references, figures and tables):

4180-128-234-115 = 3,703

For peer review only

ABSTRACT (300 word count)

Introduction

Glioblastoma (GBM) is the most common adult primary malignant brain tumour. The condition is incurable and, despite aggressive treatment at first presentation, almost all tumours recur after a median of 7 months. The aim of treatment at recurrence is to prolong survival and maintain health-related quality of life (HRQoL). Chemotherapy is typically employed for recurrent GBM, often using nitrosourea-based regimens. However, efficacy is limited, with reported median survivals between 5 and 9 months. Although less commonly used in the United Kingdom (UK), there is growing evidence that re-irradiation may produce survival outcomes at least similar to nitrosourea-based chemotherapy. However, there remains uncertainty as to the optimum approach and there is a paucity of available data, especially with regards to HRQoL. BRIOChe aims to assess re-irradiation, as an acceptable treatment option for recurrent IDH-wild-type GBM.

Methods and analysis

BRIOChe is a phase II, multi-centre, open-label, randomised trial in patients with recurrent GBM. The trial uses a Sargent's three-outcome design and will recruit approximately 55 participants from 10-15 UK radiotherapy sites, allocated (2:1) to receive re-irradiation (35 Gy in 10 daily fractions) or nitrosourea-based chemotherapy (up to six, 6-weekly cycles). The primary endpoint is overall survival rate for re-irradiation patients at 9 months. There will be no formal statistical comparison between treatment arms for the decision-making primary analysis. The chemotherapy arm will be used for calibration purposes, to collect concurrent data to aid interpretation of results. Secondary outcomes include HRQoL, dexamethasone

For peer review only

For peer review only - http://bmjopen.bmj.com/site/about/guidelines.xhtml

requirement, anti-epileptic drug requirement, radiological response, treatment compliance, acute and late toxicities, progression free survival.

Ethics and dissemination

BRIOChe obtained ethical approval from Office for Research Ethics Committees Northern Ireland (reference no. 20/NI/0070). Final trial results will be published in peer reviewed journals and adhere to the to ICMJE guidelines.

Registration details: BRIOChe (ISRCTN: 16052954; registered 25/02/2021)

For peer review only

For peer review only - http://bmjopen.bmj.com/site/about/guidelines.xhtml

STRENGTHS AND WEAKNESSES (up to 5)

- BRIOChe is multi-centre randomised trial exploring re-irradiation and chemotherapy for recurrent GBM.
- The trial will collect much needed survival and health related quality of life (HRQoL) data for patients with recurrent GBM treated with re-irradiation.
- BRIOChe focuses on the underserved population of recurrent GBM patients, for whom there is a significant lack of treatment options and UK-based trials.
- The trial design and decision criteria are based on available historical data at the time of development, where patient populations may differ from those in the BRIOChe trial.
- The trial does not provide formal comparative evidence between treatment arms. Rather the parallel concurrent control arm provides essential randomised data for benchmarking and interpretation.

For peer review only

INTRODUCTION

Glioblastoma

Glioblastoma (GBM) is the most common adult primary malignant brain tumour, with an incidence of 5/100,000 per year in England. Prognosis is poor, with an average survival of 12 to 15 months [1].

GBM is incurable, with treatment from the point of diagnosis aiming to improve survival, delay disease progression and manage symptoms/preserve HRQoL. Optimal treatment at first presentation is aggressive, including debulking surgery (if feasible), chemo-radiotherapy and adjuvant chemotherapy. However, almost all patients will relapse within the brain, with a median progression free survival (PFS) of approximately 7 months [1].

The burden placed on patients, their informal caregivers and the healthcare system as a result of neurological disability and the care requirements for patients with brain tumours is significant [2-4]. GBM is often accompanied by debilitating physical and neurological symptoms, such as paresis, sensory loss, seizures, fatigue, mood issues, and cognitive deficits [5-7]. This symptom burden can affect patients' ability to function independently and negatively impact on health-related quality of life (HRQoL) for both patients and their informal caregivers.

Recurrent glioblastoma

The optimal treatment for recurrent GBM is undefined and treatment decisions are made on a case-by-case basis, based on the site, volume and time of recurrence in relation to the original diagnosis, with consideration for patient age, co-morbidities and performance status.

In the UK, chemotherapy is commonly employed in the setting of recurrent GBM, typically using nitrosourea-based regimens. These DNA alkylating agents are highly lipophilic, and are

For peer review only

therefore able to penetrate the blood brain barrier, making them suitable for the treatment of brain tumours. Nitrosourea-combination regimens have not proven superior to single agent nitrosoureas, resulting in variations in practice as to which regimens are employed [8]. Efficacy is limited, with reported median overall survivals (OS) of between 5 and 9 months [9-17]. Side effects may include fatigue, nausea, vomiting, anorexia, constipation, paraesthesia, rash and myelosuppression. There is currently a relative lack of HRQoL data for patients receiving nitrosourea-based chemotherapy for recurrent GBM [13, 16, 18], making it difficult to determine whether the benefits of chemotherapy outweigh its burdens.

Surgery is another treatment option at point of recurrence, however, it is usually reserved for a minority of excellent performance patients with well-localised disease, although high-level evidence to support its role is lacking [9, 19-21].

Re-irradiation for recurrent glioblastoma

Re-irradiation is a further treatment option for recurrent GBM, although at present it is not commonly used in the UK. A meta-analysis of re-irradiation for recurrent GBM [22] included 50 studies of re-irradiation, of which 35 employed external beam radiotherapy (EBRT; the remainder used brachytherapy). Of the patients who received EBRT, 6-month and 12-month overall survival (OS) was 70% and 34% respectively [22]. Progression free survival (PFS) was 40% and 16% at 6 and 12-months, respectively. Grade 3+ toxicity was reported in 7% of patients. Overall, only 13 of the studies were of prospective design and only 9 were considered by the authors to be of good quality. Similarly, a systematic review of re-irradiation in GBM presented comparable outcomes based on a pooled, population-weighted analysis, where mean adjusted OS following hypofractionated EBRT was 10.1 months (95% confidence interval (CI) 9.7-10.5 months) and the rate of radionecrosis was 7.1% (95% CI: 6.6-7.7%) [23]. Despite the promising outcomes reported after re-irradiation, the poor quality of existing evidence highlights the urgent need for a high quality, prospective, randomised assessment

For peer review only

of re-irradiation, including HRQoL assessment [23, 24]. More recently, the phase II RTOG1205 trial, which randomised patients with recurrent GBM between bevacizumab (not routinely available in the UK) and bevacizumab plus re-irradiation 35Gy in 10 fractions, demonstrated a median overall survival of 10.1 months in the combination arm [25]. There was no difference in overall survival between arms, the primary endpoint of the trial, however, progression free survival was improved compared to bevacizumab alone [25]. Treatment was also well tolerated, with 5% grade 3+ acute toxicities and no delayed high grade adverse events reported.

Rationale for study

While the limited existing evidence suggests that re-irradiation has a potential positive impact on overall survival and HRQoL, to date, no trial has randomised patients with recurrent GBM between re-irradiation and chemotherapy. Addressing this research gap is a top priority for patients, informal caregivers, and healthcare professionals [26].

The BRIOChe phase II prospective trial will randomise patients with recurrent GBM to re-irradiation (35 Gy in 10 fractions, a commonly adopted re-irradiation regimen [27-32]), or nitrosourea-based chemotherapy. The aims will be to explore the efficacy and HRQoL impact of re-irradiation. This study will collect concurrent data for patients receiving chemotherapy for validation ('benchmarking') of outcomes. The study is not designed to statistically compare the two arms for the primary outcome of survival, which would require a larger number of patients. By establishing the feasibility of randomisation and providing prospective controlled data on survival and HRQoL impacts associated with re-irradiation, the BRIOChe trial aims to demonstrate re-irradiation as a possible alternative treatment option for patients with recurrent GBM and inform the design of future research.

For peer review only

For peer review only - http://bmjopen.bmj.com/site/about/guidelines.xhtml

METHODS AND ANALYSIS

Design and aim

BRIOChe is a phase II, multi-centre, open-label, randomised trial in patients with recurrent GBM. The aim of the study is to assess re-irradiation as an alternative treatment to chemotherapy in this group of patients. BRIOChe uses a Sargent's three-outcome, single-stage, single-arm design, with the inclusion of a chemotherapy calibration arm for concurrent data for benchmarking. Following an amendment due to slow recruitment, the trial will now recruit approximately 55 participants from 10-15 UK radiotherapy sites. The original sample size was 70 based on a 1:1 allocation ratio, to receive either re-irradiation or nitrosourea-based chemotherapy (lomustine +/- procarbazine +/- vincristine). The allocation ratio was adapted during recruitment to 2:1, further details are presented in the sample size section.

The trial schema is presented in Figure 1.

For peer review only

Trial objectives

The primary objective of BRIOChe is to assess the proportion of participants alive at 9 months post start of treatment in the re-irradiation arm.

The key secondary objective of BRIOChe is to evaluate the effect of re-irradiation on HRQoL. Questionnaires to be completed by participants include the European Organization for Research and Treatment of Cancer (EORTC) Quality of life questionnaire core 30 (QLQ-C30) and Brain Cancer module (BN20). HRQoL will also be explored in greater detail through one-off semi-structured qualitative interviews in a subsample of patients and their informal caregivers.

Additional secondary objectives include evaluating:

- Dexamethasone requirement
- Anti-epileptic drug requirement
- Radiological response in accordance with Response Assessment in Neuro-Oncology (RANO) criteria
- Treatment compliance
- Acute (measured from randomisation to 12 weeks post end of treatment of treatment) and late toxicities (after 12 weeks post end of treatment) as per Common Terminology Criteria for Adverse Events (CTCAE V.5.0.); radionecrosis is a late toxicity of particular interest
- PFS
- OS

Study population

All inclusion/exclusion criteria must be met and written informed consent obtained prior to recruitment (Key criteria summarised in Table 1); full criteria (Supplementary File 1) and a sample patient consent form (Supplementary File 2) are included as Supplementary Material.

For peer review only

The formal assessment of eligibility and informed consent may only be obtained by the Principal Investigator (PI) or an appropriate medically qualified doctor. Patients will be screened from clinic lists. Disease eligibility will be based on the diagnostic MRI scan where recurrence is identified. For patients who are randomised to re-irradiation, if significant progression is observed on the re-irradiation planning imaging (with GTV volume >75cm3 or doubling compared to the baseline MRI), such cases will be reviewed with the RTTQA group before proceeding with re-irradiation. Patients who are randomised to chemotherapy who remain clinically stable would not routinely receive updated imaging prior to commencement of chemotherapy but in these cases the interval to starting chemotherapy is anticipated to be shorter than that to starting re-irradiation, providing less time for significant progression.

Table 1– Summary of key inclusion and exclusion criteria

**Inclusion criteria**	**Exclusion criteria**
Histologically proven diagnosis of GBM, based on original pathology.	Pregnant (positive pregnancy test) or lactating
First recurrence of GBM, with contrast enhancing disease, following primary treatment	Critical normal brain structures treated above usual tolerance during initial radiotherapy
Agreement of a Consultant Neuro-Radiologist that imaging changes are in keeping with recurrence and not pseudo-progression.	Recurrence with leptomeningeal disease or only leptomeningeal disease
≥6 months since completion of primary radiotherapy	More than three enhancing lesions present on MRI or multi-focal recurrence
Prior history of standard dose, conventionally fractionated CNS radiotherapy	IDH1/2 mutant tumours on original pathology
As a minimum, patients will have completed at least two weeks of temozolomide, concurrent with their original radiotherapy.	GBM with known features of PXA, BRAF mutations or 1p19q co-deletion (on original pathology or updated pathology if available)
Up to and including three enhancing lesions (size and volume criteria included in Supplementary Material)	Prior invasive malignancy (except non-melanomatous skin cancer), unless disease free for a minimum of one year.
Karnofsky Performance Status 70+	Severe active co-morbidity making patient unsuitable for chemotherapy or re-irradiation
Adequate hematologic, renal, and hepatic function	Prior allergic reaction to nitrosoureas
No contra-indication to lomustine	Any recognised genetic syndromes causing sensitivity to radiotherapy

For peer review only - http://bmjopen.bmj.com/site/about/guidelines.xhtml

Patients must be able to provide study-specific informed consent.	Contra-indication to MRI or gadolinium
Age 18 or over.	Previous radiotherapy dose distribution unavailable
	Previous systemic therapy or re-irradiation for recurrent GBM

Randomisation and recruitment

Randomisation will be performed centrally using University of Leeds Clinical Trials Research Unit (CTRU) automated 24-hour randomisation system. A computer-generated minimisation program incorporating a random element will be used to ensure the treatment groups are well balanced for the following factors:

- Age (<50 or ≥50 years)
- Time to randomisation from completion of previous radiotherapy (≤12 months or >12 months)
- MGMT promoter methylation status (Methylated, Unmethylated or Unknown)
- Randomising site

The intended recruitment period is 2-years (BRIOChe opened to recruitment on 22/06/2021).

Sample size

Thirty-three patients are required in the re-irradiation arm, 35 allowing for 5% drop out. With a 1:1 randomisation allocation ratio to include a calibration arm, the original sample size was 70 patients in total. As a result of slow recruitment, the randomisation allocation ratio was adapted in a protocol amendment to 2:1 (re-irradiation: chemotherapy), to allow a higher proportion of recruited patients into the intervention arm. The subsequent sample size was estimated to be 55 patients in total, with 35 in the re-irradiation arm and approximately 20 in chemotherapy arm.

For peer review only

For peer review only - http://bmjopen.bmj.com/site/about/guidelines.xhtml

This study is powered to demonstrate that the treatment strategy of re-irradiation demonstrates sufficient efficacy to warrant further large-scale evaluation, based on 9-month survival rates.

A Sargent's three-outcome, phase II, single-stage, single-arm design will be used to determine whether re-irradiation demonstrates sufficient efficacy to warrant further evaluation [33]. The trial is designed to test the null hypothesis (H0) that the proportion of participants alive at 9 months is <25% where re-irradiation would not be deemed worthy of further investigation, against an alternative hypothesis H1 of >45% where re-irradiation would be deemed worthy of further investigation. If the proportion of participants alive at 9 months is ≥25% and ≤45%, this is an inconclusive region where neither the null or alternative hypothesis would be rejected and the decision regarding further investigation would be based on other factors. The following operating characteristics are used: type I error=0.05, type II error=0.06, prob (correctly rejecting the H1) = 0.82 (eta), prob (correctly rejecting the H0) =0.79 (pi).

Treatment regimen

Re-irradiation arm: The radiotherapy prescription dose will be 35 Gy in 10 fractions, a commonly employed schedule [27-32], delivered daily Monday-Friday over two weeks. Treatment will be delivered with Intensity Modulated Radiotherapy (IMRT)/ Volumetric Modulated Arc Therapy (VMAT) or tomotherapy. Optimally, normal tissue constraints will be based on a 'dose remaining' approach, whereby an original organ at risk (OAR) dose (allowing for 25% repair) is subtracted from a cumulative tolerance to give a 'dose remaining' for re-irradiation. To save clinicians time and reduce errors in performing such calculations, the radiotherapy guidelines contain tables of 'OAR doses remaining' for ease of reference. Where these optimal constraints cannot be achieved, mandatory constraints are those used in the RTOG1205 trial, which also delivered a dose of 35 Gy in 10 fractions [31]. Treatment

For peer review only

compliance data to radiotherapy, including fractions, doses, interruptions and reasons will be collected weekly during treatment.

Chemotherapy arm: As per usual UK practice in the setting of relapsed GBM where the interval to recurrence is insufficiently long to use re-challenge temozolomide and/or when patients have O^6-methylguanine-DNA-methyltransferase (MGMT) unmethylated tumours, chemotherapy will be lomustine-based. These drugs have been used in this setting for many years. A variety of lomustine-based regimens are used across the UK including single agent lomustine, PCV (procarbazine, lomustine and vincristine), PC (procarbazine and lomustine) or CV (lomustine and vincristine). Lomustine-based regimens will be delivered as per each centre's standard practice. Doses will be recorded at each cycle and (based on a survey of participating centres regarding their usual practice) it is recommend that these will consist of:

- Lomustine 100-130mg/m^2, day 1, PO, on a 42 day cycle

  +/-

- Procarbazine 50-100mg/m^2, once daily on days 1-10 or days 2-11, PO, on a 42 day cycle

  +/-

- Vincristine 1.4-1.5mg/m^2, with dose capping as per the institution's usual practice, IV day 1, on a 42 day cycle.

(Dose capping is as per the institution's usual practice).

Treatment compliance data to chemotherapy will be collected from patient diaries. Dose modifications and reasons for changes will also be collected. Suggested dose modifications to chemotherapy based on haematological, renal and hepatic functioning are provided in the study protocol.

Upon further disease progression, patients may receive the alternative treatment according to local policy.

For peer review only

Trial assessments and follow-up

Full eligibility criteria (Table 1, Supplementary Material), will be assessed and confirmed prior to randomisation. Further assessments will be conducted at multiple timepoints. An overview of data collection can be seen in Table 2. Data will be collected from all participants, until progression or death. Where available, date of death will be collected for all participants. A final data sweep prior to the final analysis will collect information regarding death and any additional anti-cancer treatment received beyond progression.

Follow-up imaging with MRI will include T2, FLAIR (fluid attenuated inversion recovery), DWI (diffusion weighted imaging), T1 pre-gadolinium and T1 post-gadolinium sequences. In addition, patients randomised to re-irradiation will also undergo T2* Dynamic Susceptibility Contrast (DSC) perfusion as part of follow-up imaging to assist in determination of the presence of radionecrosis.

For peer review only

Table 2 – Trial assessment schedules

	Early assessments				Follow-up assessment (measured post start of treatment)									
	Eligibility	Baseline	Pre-treat RT	Pre-treat chemo	0w	1w	6w	12w	18w	24w	30w	36w	42w	48w
Informed consent	X													
Clinical assessment	X				X	X***	X	X	X	X	X	X	X	X
Performance status (Karnofsky)	X				X	X***	X	X	X	X	X	X	X	X
Bloods*	X			X	X	X	X	X	X	X	X			
Pregnancy screening**	X		X		X	X	X	X	X	X	X			
Re-irradiation					X	X								
Chemotherapy					X		X	X	X	X	X			
MRI (RANO criteria)	X						X			X				
Toxicity (CTCAE)		X			X	X***	X	X	X	X	X	X	X	X
HRQoL		X					X	X	X	X	X	X	X	X

* Bloods only for Eligibility for re-irradiation arm, with each cycle in chemotherapy arm

**only at pretreatment for re-irradiation arm, with every cycle in chemotherapy arm

*** re-irradiation arm patients only

Adverse Events (AEs), Serious Adverse Events (SAEs), Adverse Reaction (ARs), Serious Adverse Reactions (SARs) and Suspected Unexpected Serious Adverse Reactions (SUSARs) will all be collected from randomisation until the last participant follow-up or disease progression. Beyond this period only, SARs and SUSARs related to the trial treatment (and not GBM progression) will be reported if the investigator becomes aware of them until the end of trial notification. All SAEs/SARs and SUSARs related to investigation medicinal products (IMPs) will be reported to the Medicines and Healthcare products Regulatory Agency (MHRA) and sponsor. All radiotherapy related SAEs/SARs and SUSARs will be reported to the Research Ethics Committee and sponsor.

For peer review only - http://bmjopen.bmj.com/site/about/guidelines.xhtml

Statistical analysis

The statistical analysis will be conducted by University of Leeds CTRU. A full statistical analysis plan has been developed and finalised.

The trial analysis will be conducted on a Modified Intention to Treat (MITT) population [34]. MITT is defined as all participants randomised that have received at least one dose of trial treatment, analysed according to the treatment arm they were randomised to (regardless of ineligibility, non-compliance or withdrawal).

Overall survival rates at 9 months, i.e. the number and proportion of participants alive at 9 months post-start of treatment, will be calculated for the re-irradiation arm with 90% (corresponding to a 1-sided 5% significance level used in the design) and 95% confidence intervals (CIs).

Based on the 3-outcome design, the cut-off values and conclusions for the statistical test for the primary analysis of efficacy are defined as follows:

Green: If ≥13/33 patients are alive, re-irradiation demonstrates sufficient efficacy to warrant larger-scale evaluation. This is based on a 9-month OS >45% (i.e. competitive with the 'best' outcomes from prospective studies using nitrosourea-based chemotherapy [11, 14, 35].

Amber: If 11-12/33 patients are alive the decision to take re-irradiation forward will be uncertain and will be based on HRQoL and toxicity at 3 months, (this is based on 9-month OS 25%-45%; i.e. similar to outcomes from other prospective nitrosourea-based trials [13, 16, 17, 36].

Red: If ≤10/33 patients are alive re-irradiation will not be considered worthy of further investigation, based on 9-month OS <25%.

For peer review only

The chemotherapy arm is included for calibration and will be used for validation of outcomes in the re-irradiation group with respect to previous series, and validation of the assumptions used to inform the statistical cut-offs.

Summary statistics will be calculated for all domains of the EORTC QLQ-C30 and QLQ-BN20 and the overall summary score calculated, presented overall and by treatment arm for each of the follow-up time points. The difference in scores between treatment arms will be presented with corresponding 90% and 95% confidence intervals.

Summary statistics will be presented for dexamethasone requirement, anti-epileptic drug requirement, treatment compliance, and response assessment; including the proportion of participants within each clinical response status. The maximum CTCAE grade of toxicities experienced by participants will be summarised for each CTCAE term reported, by treatment arm, for the acute and late toxicities.

The assessment of PFS and OS will be based on a time to event analysis and presented using Kaplan Meier curves. Median PFS and OS will be presented with 95% CIs for each arms. There will be no formal comparison between arms, however, the unadjusted OS hazard ratio (HR) for the re-irradiation arm vs the chemotherapy arm will be presented.

Qualitative interviews

Qualitative exploration of HRQoL associated with brain tumour treatments will allow more in-depth evaluation of what matters most to patients and informal caregivers at the time of GBM recurrence; their perception of HRQoL before and during trial treatment; and how they experience both the treatment they receive and trial participation. Interviews will be recorded, transcribed, and thematically analysed [37]. A sub-sample of trial participants will be asked to partake in this interview, around 15 patients and their informal caregivers. We will utilise a maximum variation sample, to choose patients (and their informal caregivers) that differ in age, sex and previous/current treatment(s)

For peer review only

received. By using a varied sample, we aim to highlight important shared patterns that cut across cases and also discover unique or diverse variations from different participants [38].

Trial organisation

University of Leeds CTRU will manage all trial and data co-ordination according to the unit's well established standard operating procedures. All trial organisation will be conducted in-line with the principles of Good Clinical Practice.

Data collection and management

The BRIOChe trial data collection has been designed for remote data entry. However, all safety data will be recorded on paper case report forms and Patient Reported Outcome Measures (PROMs) on the relevant paper questionnaires, sent to and entered at CTRU. Participating centres will record and complete patient data on a trial specific database. The database has instantaneous validation checking. In addition, data management validation reports for missing and discrepant data.

All trial data is stored confidentially in a secure location at University of Leeds CTRU. Access to trial data will be restricted to CTRU staff and study team only, prior to the analysis and release of the trial results. Upon completion of the trial and publishing of results, researchers can request access to data by contacting members of the BRIOChe trial management group (TMG) or CTRU. All data will be stored and archived for a minimum of 25 years after trial completion.

For peer review only

For peer review only - http://bmjopen.bmj.com/site/about/guidelines.xhtml

Quality assurance

The radiotherapy quality assurance (QA) programme will be implemented by the National Cancer Research Institute (NCRI) Radiotherapy Trials Quality Assurance (RTTQA) group to ensure treatment is planned and delivered according to the trial protocol. RTTQA includes both benchmarking contouring and planning cases. A summary of the RTTQA requirements are provided in the BRIOChe radiotherapy outlining, planning, treatment delivery and QA guidelines. All sites must complete essential documentation and the site initiation process before a site can be activated. The site initiation will be an electronic process including an audio-visual recording. The video recorded site initiation presentation slides can be used for future training and reference at participating sites.

Trial monitoring

The BRIOChe trial will be monitored by the specific project delivery group at CTRU and the multi-disciplinary TMG. An independent data monitoring and ethics committee (DMEC) will be responsible for the safety and integrity of the patients and study, monitoring unblinded interim data. The DMEC will be formed of two independent clinical oncologists and one independent statistician, meeting at least annually, in addition to receiving 6-monthly safety reports. The DMEC members will advise the Trial Steering Committee (TSC) on any trial or safety concerns. The TSC have definitive control over the continuation of the trial. A DMEC charter has been developed including the roles and responsibilities, and communication processes.

Patient and Public Involvement

Patient and Public Involvement (PPI) has been an instrumental part of the development and conduct of the BRIOChe trial. The trial design was discussed with and presented to

For peer review only

For peer review only - http://bmjopen.bmj.com/site/about/guidelines.xhtml

PPI representatives. The trial question was felt to be of importance and the treatments and assessment schedule were considered acceptable.

The Trial Management Group includes a key patient representative, who provides ongoing and regular advice regarding trial conduct from the patient perspective.

Imaging sub-study

An imaging sub-study is planned between two of the recruiting centres (Leeds Teaching Hospitals NHS Trust and University College London Hospitals NHS Foundation Trust), to investigate the impact of re-irradiation and chemotherapy treatments on cerebral vascular changes and to improve understanding as to how these changes might be associated with both tumour response and normal tissue damage. The imaging sub-study is estimated to recruit 12-16 patients from the full BRIOChe trial. Sub-study participants who have been randomised to re-irradiation will have Arterial Spin Labelling (ASL) and T2 Dynamic Susceptibility Contrast (DSC) sequences added to the dedicated radiotherapy planning MRI. In addition, these participants will have ASL added to their routine follow up MRI scans (T2 DSC already included), to provide 'before' and 'after' re-irradiation information. Those sub-study participants randomised to chemotherapy will also have ASL and T2 DSC sequences added to their routine follow-up MRI to allow comparison between chemotherapy-treated and re-irradiated participants.

For peer review only

ETHICS AND DISSEMINATION

The trial obtained ethical approval from the Office for Research Ethics Committees Northern Ireland (ORECNI) (reference no. 20/NI/0070) and is registered in the ISRCTN registry (registration number 16052954). The trial is currently adhering to protocol version 4.0 (7/12/2022). Any protocol amendments will be submitted to MHRA and REC, all changes will be communicated with local sites.

There is no formal interim analysis. The final trial data will be analysed and reported approximately one year after the final participant is recruited. The final trial result manuscript will be written in accordance to ICMJE guidelines and submitted for publication to peer reviewed journals.

DISCUSSION

Despite the poor outcomes and high clinical need, there is a lack of active clinical trials for patients with recurrent GBM in the UK and no gold standard treatment at recurrence exists. Chemotherapy is often used in the UK, but outcomes are disappointing. Re-irradiation is recognised as an alternative treatment option and is listed as such in European and American treatment guidelines for patients with recurrent GBM, and in the current NICE guidelines for brain tumours [39-41]. Despite this, re-irradiation is less commonly utilised in the UK compared to other parts of the world. Re-irradiation offers a shorter course of treatment compared to chemotherapy and has a different toxicity profile, and so may be preferable for some patients, although the risks and benefits need further clarification. The BRIOChe trial aims to be a pragmatic, clinically relevant trial that evaluates re-irradiation as an alternative treatment option to chemotherapy for patients with recurrent GBM. While OS is the primary end point, HRQoL is a key secondary endpoint.

For peer review only - http://bmjopen.bmj.com/site/about/guidelines.xhtml

The trial design and treatment, specified in BRIOChe, intentionally reflects routine practice. Centres are able to use their usual lomustine-based chemotherapy regimen(s) thus reflecting the variety in practice across the UK. The frequently used and relatively well-tolerated hypofractionated schedule, 35Gy in 10 fractions, has been adopted for BRIOChe re-irradiation regimen [27-30] .

No organ at risk (OAR) constraints have been formally validated for GBM re-irradiation. Most GBM recurrences occur within the previous high dose region and so it is often necessary to assume that a degree of OAR recovery has occurred following the original course of radiotherapy if a meaningful dose is to be delivered to the re-irradiation target. As such, BRIOChe provides 'radiobiological' optimal constraints, allowing for 25% recovery from the original radiotherapy, based on limited evidence regarding post-irradiation recovery in neural structures [42-44]. In addition, where optimal constraints limit re-irradiation delivery, the more lenient mandatory constraints have been based on the RTOG-1205 trial [45]. Encouragingly since the opening of the BRIOChe trial, results from the RTOG1205 trial have been published indicating that re-irradiation, 35Gy in 10 fractions, as used in BRIOChe, in combination with bevacizumab, results in a median overall survival of 10.1 months [25]. In addition, re-irradiation was well tolerated [25]. However, re-irradiation alone was not investigated in RTOG1205, BRIOChe will investigate this further.

The eligibility criteria volume requirements consider whether the lesion is single or multi-focal. Size and volume limitations were included to limit the amount of normal brain re-irradiation in an effort to keep the risk of radionecrosis low. This approach was preferred over using 'diameter only' measurement as this could potentially exclude long but narrow recurrences, where the total volume was low.

For peer review only

The minimal acceptable interval between completion of original radiotherapy and commencement of re-irradiation is also unknown and this interval varies amongst existing studies. We have adopted a minimum interval of 6 months from completion of first radiotherapy in BRIOChe, which is in-keeping with current practices in other countries [46] and international trials using the same re-irradiation schedule [31, 32]. An interval of at least 6 months also allows some time for OAR repair, should repair occur, and reduces the risk of mistaking pseudo-progression for true progression, which typically occurs within 3 months of radiotherapy [47].

It is acknowledged as a limitation that the trial design and decision criteria are based on available historical data at the time of development. The referenced studies of nitrosourea-based chemotherapy required intervals between recurrence to treatment that are shorter than the 6 months required in order to enter BRIOChe, potentially selecting some patients that come from a slightly better prognosis patient group. The trial has taken a pragmatic approach given the paucity of data with the exact criteria for those treated with systemic therapy.

While the patient numbers involved will not permit a direct comparison of survival outcomes in each arm, the BRIOChe trial aims to demonstrate that re-irradiation is a possible alternative treatment option for patients with recurrent GBM, including from the patient perspective. This in turn will allow the development of future novel approaches, including potential treatment intensification strategies using re-irradiation in combination with novel agents such as immunotherapies and radiosensitisers, to be explored in large-scale platform studies.

For peer review only

The BRIOChe study will provide valuable data for patients with recurrent GBM treated with re-irradiation and nitrosourea-based chemotherapy, forming a first step in improving treatment for this poor prognosis patient group.

For peer review only

AUTHOR CONTRIBUTIONS

Contributors to the conception, trial design and ongoing conduct: LM, FB, SCS, EH, SN, JW, SB, OA, HBe, HBu, SC, SF, JH, JL, AS, CP, FS, DSM.

Development of the protocol, trial documents and patient information sheet: EH, LM, SB, SN, FB, CP, SC, JL.

Writing manuscript: EH, LM.

Reviewed manuscript: all authors

All authors read and approved the final manuscript.

FUNDING, SPONSOR AND COORDINATION

BRIOChe is supported by The Jon Moulton Charity Trust (NA), and by Yorkshire Cancer Research (YCR), Centre for Early Phase Clinical Trials (L375PA). The imaging sub-study is supported by Cancer Research UK funding for the Leeds Radiotherapy Research Centre of Excellence (RadNet; C19942/A28832).

The study is coordinated by University of Leeds CTRU and sponsored by University of Leeds (GA18/118305). For enquires, trial contact *email:* BRIOCHE@leeds.ac.uk. The sponsor and funders have had no influence on the BRIOChe trial study design, collection, management, analysis, and interpretation of data; and decision for publication. The sponsor and funder will review all manuscripts prior to publication.

COMPETING INTERESTS

FS reports grants from Cancer Research UK, during the conduct of the study. DSM reports grants from Jon Moulton Charitable Foundation, grants from Yorkshire Cancer Research,

For peer review only

during the conduct of the study. LM reports grants from Jon Moulton Charity Trust, grants from Cancer Research UK, during the conduct of the study; grants from Yorkshire Cancer Research, outside the submitted work. SB reports grants from Jon Moulton Charitable Foundation, grants from Yorkshire Cancer Research, during the conduct of the study. SS reports advisory board activity for Bayer, CeCaVa Laboratory Research project support, Apollomics.

For peer review only

EXCLUSIVE LICENCE

I, the Submitting Author has the right to grant and does grant on behalf of all authors of the Work (as defined in the below author licence), an exclusive licence and/or a non-exclusive licence for contributions from authors who are: i) UK Crown employees; ii) where BMJ has agreed a CC-BY licence shall apply, and/or iii) in accordance with the terms applicable for US Federal Government officers or employees acting as part of their official duties; on a worldwide, perpetual, irrevocable, royalty-free basis to BMJ Publishing Group Ltd ("BMJ") its licensees and where the relevant Journal is co-owned by BMJ to the co-owners of the Journal, to publish the Work in BMJ Open and any other BMJ products and to exploit all rights, as set out in our licence.

The Submitting Author accepts and understands that any supply made under these terms is made by BMJ to the Submitting Author unless you are acting as an employee on behalf of your employer or a postgraduate student of an affiliated institution which is paying any applicable article publishing charge ("APC") for Open Access articles. Where the Submitting Author wishes to make the Work available on an Open Access basis (and intends to pay the relevant APC), the terms of reuse of such Open Access shall be governed by a Creative

Commons licence – details of these licences and which Creative Commons licence will apply to this Work are set out in our licence referred to above.

FIGURE LEGEND: Figure 1 - BRIOChe Trial schema

For peer review only

For peer review only - http://bmjopen.bmj.com/site/about/guidelines.xhtml

REFERENCES

1. Stupp, R., et al., *Radiotherapy plus concomitant and adjuvant temozolomide for glioblastoma.* New England journal of medicine, 2005. **352**(10): p. 987-996.
2. Gately, L., et al., *Life beyond a diagnosis of glioblastoma: a systematic review of the literature.* Journal of Cancer Survivorship, 2017. **11**(4): p. 447-452.
3. Mummudi, N. and R. Jalali, *Palliative care and quality of life in neuro-oncology.* F1000prime reports, 2014. **6**.
4. Arvold, N.D., et al., *Hospitalization burden and survival among older glioblastoma patients.* Neuro-oncology, 2014. **16**(11): p. 1530-1540.
5. Mukand, J.A., et al., *Incidence of neurologic deficits and rehabilitation of patients with brain tumors.* American journal of physical medicine & rehabilitation, 2001. **80**(5): p. 346-350.
6. Boele, F.W., et al., *Symptom management and quality of life in glioma patients.* CNS oncology, 2014. **3**(1): p. 37-47.
7. Sterckx, W., et al., *The impact of a high-grade glioma on everyday life: a systematic review from the patient's and caregiver's perspective.* European Journal of Oncology Nursing, 2013. **17**(1): p. 107-117.
8. Weller, M., et al., *Standards of care for treatment of recurrent glioblastoma—are we there yet?* Neuro-oncology, 2013. **15**(1): p. 4-27.
9. van Linde, M.E., et al., *Treatment outcome of patients with recurrent glioblastoma multiforme: a retrospective multicenter analysis.* Journal of neuro-oncology, 2017. **135**(1): p. 183-192.
10. Das, P., S. McCaffrey, and D. Conkey, *Re-evaluating the efficacy and toxicity of procarbazine, lomustine and vincrsitine (PCV) in relapsed glioblastoma in the concurrent/ adjuvant temozolamide era.* Journal of Cancer Prevention and Current Research, 2016. **6**(3): p. 1-6.
11. Taal, W., et al., *Single-agent bevacizumab or lomustine versus a combination of bevacizumab plus lomustine in patients with recurrent glioblastoma (BELOB trial): a randomised controlled phase 2 trial.* The lancet oncology, 2014. **15**(9): p. 943-953.
12. Carvalho, B.F., et al., *Second-line chemotherapy in recurrent glioblastoma: a 2-cohort study.* Oncology research and treatment, 2015. **38**(7-8): p. 348-354.
13. Brada, M., et al., *Temozolomide versus procarbazine, lomustine, and vincristine in recurrent high-grade glioma.* J Clin Oncol, 2010. **28**(30): p. 4601-4608.
14. Wick, W., et al., *Lomustine and bevacizumab in progressive glioblastoma.* New England Journal of Medicine, 2017. **377**(20): p. 1954-1963.
15. Rajan, B., et al., *Survival in patients with recurrent glioma as a measure of treatment efficacy: prognostic factors following nitrosourea chemotherapy.* European Journal of Cancer, 1994. **30**(12): p. 1809-1815.
16. Wick, W., et al., *Phase III study of enzastaurin compared with lomustine in the treatment of recurrent intracranial glioblastoma.* Journal of clinical oncology, 2010. **28**(7): p. 1168.
17. Lombardi, G., et al. *Updated results of REGOMA: A randomized, multicenter, controlled open-label phase II clinical trial evaluating regorafenib in relapsed glioblastoma (GBM) patients (PTS)*. in *Oral Presentation at EANO (Eurpoean Association of Neuro-Oncolgy) Annual conference*. 2018.
18. Dirven, L., et al., *Dutch Neuro-Oncology Group (LWNO). The impact of bevacizumab on health-related quality of life in patients treated for recurrent glioblastoma: results of the randomised controlled phase 2 BELOB trial.* Eur J Cancer, 2015. **51**(10): p. 1321-1330.
19. Pessina, F., et al., *Role of surgical resection in recurrent glioblastoma: prognostic factors and outcome evaluation in an observational study.* Journal of neuro-oncology, 2017. **131**(2): p. 377-384.
20. Franceschi, E., et al., *The effect of re-operation on survival in patients with recurrent glioblastoma.* Anticancer research, 2015. **35**(3): p. 1743-1748.

For peer review only - http://bmjopen.bmj.com/site/about/guidelines.xhtml

21. Gorlia, T., et al., *New prognostic factors and calculators for outcome prediction in patients with recurrent glioblastoma: a pooled analysis of EORTC Brain Tumour Group phase I and II clinical trials.* European journal of cancer, 2012. **48**(8): p. 1176-1184.
22. Kazmi, F., et al., *Re-irradiation for recurrent glioblastoma (GBM): a systematic review and meta-analysis.* Journal of neuro-oncology, 2019. **142**(1): p. 79-90.
23. Shanker, M., et al., *Re-irradiation for recurrent high-grade gliomas: a systematic review and analysis of treatment technique with respect to survival and risk of radionecrosis.* Neuro-Oncology Practice, 2019. **6**(2): p. 144-155.
24. Nieder, C., et al., *Improvement, clinical course, and quality of life after palliative radiotherapy for recurrent glioblastoma.* American journal of clinical oncology, 2008. **31**(3): p. 300-305.
25. Tsien, C.I., et al., *NRG Oncology/RTOG1205: a randomized phase II trial of concurrent bevacizumab and reirradiation versus bevacizumab alone as treatment for recurrent glioblastoma.* Journal of clinical oncology, 2023. **41**(6): p. 1285-1295.
26. Grant, R., et al., *OP01THE UK TOP 10 CLINICAL RESEARCH PRIORITIES IN NEURO-ONCOLOGY.* Neuro-Oncology, 2015. **17**(Suppl 8): p. viii16.
27. Klobukowski, L., et al., *A retrospective review of re-irradiating patients' recurrent high-grade gliomas.* Clinical Oncology, 2018. **30**(9): p. 563-570.
28. Fogh, S.E., et al., *Hypofractionated stereotactic radiation therapy: an effective therapy for recurrent high-grade gliomas.* Journal of clinical oncology, 2010. **28**(18): p. 3048.
29. Hudes, R.S., et al., *A phase I dose escalation study of hypofractionated stereotactic radiotherapy as salvage therapy for persistent or recurrent malignant glioma.* International Journal of Radiation Oncology* Biology* Physics, 1999. **43**(2): p. 293-298.
30. Tsien, C., et al., *ACTR-32. NRG ONCOLOGY RTOG 1205: randomized phase II trial of concurrent bevacizumab and re-irradiation vs. bevacizumab alone as treatment for recurrent glioblastoma.* Neuro-Oncology, 2019. **21**(Suppl 6): p. vi20.
31. RTOG 1205. *Randomized phase II trial of concurrent bevacizumab and re-irradiation versus bevacizumab alone as treatment of recurrent glioblastoma.* 2016; Available from: https://clinicaltrials.gov/ct2/show/NCT01730950.
32. AstraZeneca, *AZD1390 Radiation Procedure Manual. Version 1.0.* 2017.
33. Sargent, D.J., V. Chan, and R.M. Goldberg, *A three-outcome design for phase II clinical trials.* Controlled clinical trials, 2001. **22**(2): p. 117-125.
34. Abraha, I. and A. Montedori, *Modified intention to treat reporting in randomised controlled trials: systematic review.* Bmj, 2010. **340**.
35. Batchelor, T.T., et al., *Phase III randomized trial comparing the efficacy of cediranib as monotherapy, and in combination with lomustine, versus lomustine alone in patients with recurrent glioblastoma.* Journal of Clinical Oncology, 2013. **31**(26): p. 3212.
36. Boiardi, A., et al., *Efficacy of '8-drugs-in-one-day'combination in treatment of recurrent GBM patients.* Journal of neuro-oncology, 1992. **12**(2): p. 153-158.
37. Fereday, J. and E. Muir-Cochrane, *Demonstrating rigor using thematic analysis: A hybrid approach of inductive and deductive coding and theme development.* International journal of qualitative methods, 2006. **5**(1): p. 80-92.
38. Patton, M., *Pattern, theme, and content analysis*, in *Qualitative research & evaluation methods*. 2002, Sage Publications Ltd, London. p. 452-471.
39. Cabrera, A.R., et al., *Radiation therapy for glioblastoma: executive summary of an American Society for Radiation Oncology evidence-based clinical practice guideline.* Practical radiation oncology, 2016. **6**(4): p. 217-225.
40. Stupp, R., et al., *High-grade glioma: ESMO Clinical Practice Guidelines for diagnosis, treatment and follow-up.* Annals of Oncology, 2014. **25**: p. iii93-iii101.
41. NICE, N.I.f.H.a.C.E., *Brain tumours (primary) and brain metastases in adults.* NICE guideline [NG99], 2018.
42. Kirkpatrick, J.P., A.J. van der Kogel, and T.E. Schultheiss, *Radiation dose–volume effects in the spinal cord.* International Journal of Radiation Oncology* Biology* Physics, 2010. **76**(3): p. S42-S49.

For peer review only - http://bmjopen.bmj.com/site/about/guidelines.xhtml

43. Ang, K.K., et al., *Extent and kinetics of recovery of occult spinal cord injury.* International Journal of Radiation Oncology* Biology* Physics, 2001. **50**(4): p. 1013-1020.
44. Flickinger, J.C., M. Deutsch, and L.D. Lunsford, *Repeat megavoltage irradiation of pituitary and suprasellar tumors.* International Journal of Radiation Oncology* Biology* Physics, 1989. **17**(1): p. 171-175.
45. Tsien, C., et al., *Randomized phase II trial of re-irradiation and concurrent bevacizumab versus bevacizumab alone as treatment for recurrent glioblastoma (NRG Oncology/RTOG 1205): initial outcomes and RT plan quality report.* International Journal of Radiation Oncology Biology and Physics, 2019. **105**(1).
46. Post, C.C., et al., *Patterns of re-irradiation for recurrent gliomas and validation of a prognostic score.* Radiotherapy and Oncology, 2019. **130**: p. 156-163.
47. De Wit, M., et al., *Immediate post-radiotherapy changes in malignant glioma can mimic tumor progression.* Neurology, 2004. **63**(3): p. 535-537.

For peer review only

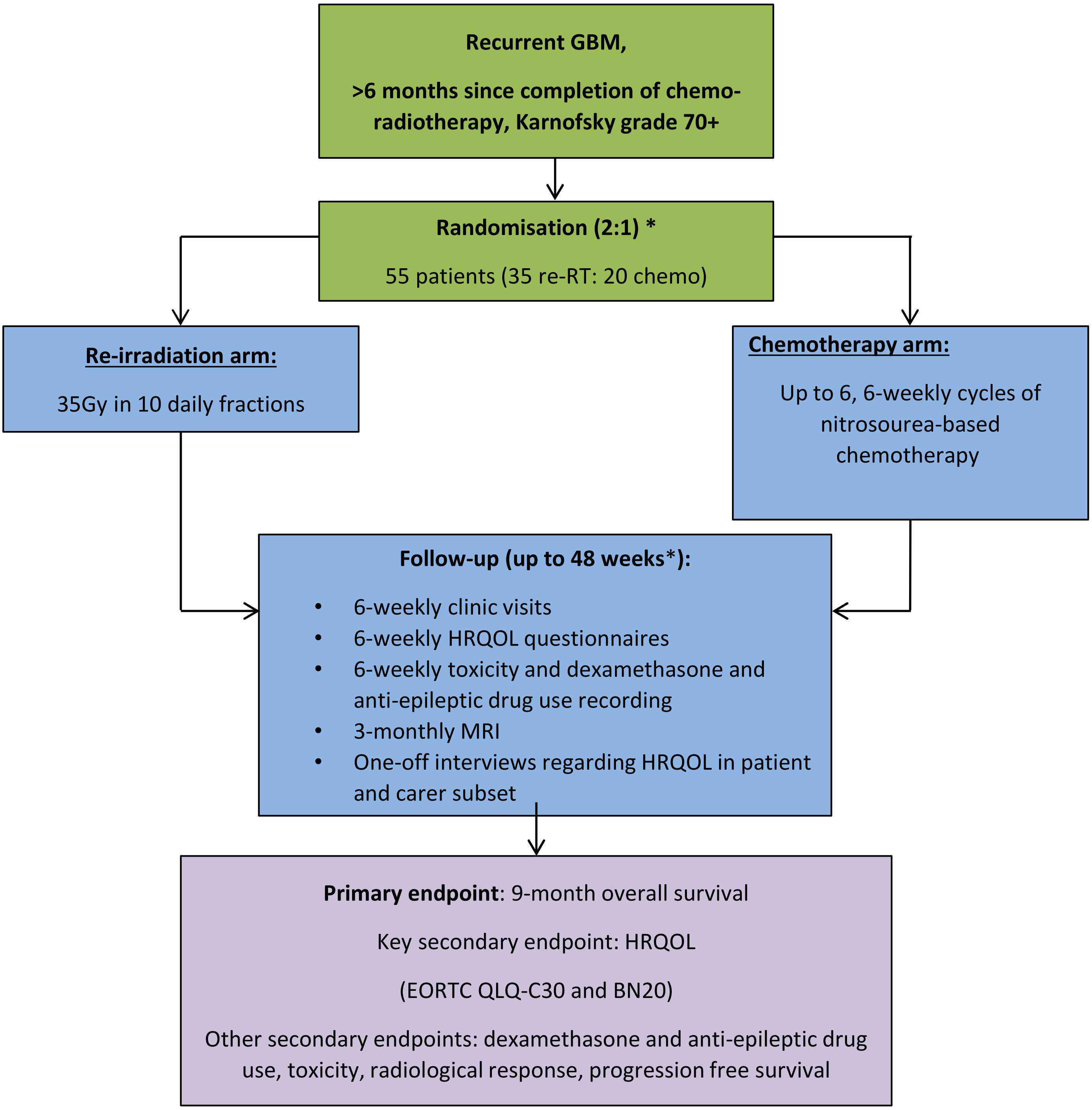

**allocation ratio originally 1:1, ratio was adapted to 2:1 during the recruitment phase*

EORTC QLQ-C30 and BN20, European Organization for Research and Treatment of Cancer Quality of life questionnaire core 30 and Brain Cancer module 20; GBM, glioblastoma multiforme; HRQoL, health-related quality of life; MRI, magnetic resonance imaging;

SUPPLEMENTARY FILE 1 – Full inclusion exclusion criteria

Inclusion criteria	Exclusion criteria
1. Histologically proven diagnosis of GBM with consistent molecular pathology, based on original pathology.	1. Pregnant (positive pregnancy test) or lactating
2. First recurrence of GBM, with contrast enhancing disease, following primary treatment (i.e. no previous systemic therapy or re-irradiation for recurrence).	2. Critical normal brain structures treated above usual tolerance during initial radiotherapy
3. Agreement of a Consultant Neuro-Radiologist within multi-disciplinary team meeting that imaging changes are in keeping with recurrence and not pseudo-progression.	3. Recurrence with leptomeningeal disease or only leptomeningeal disease
4. Randomisation must be performed within 21 days of the MRI that confirms recurrence.	4. Recurrence defined by non-enhancing disease only
5. ≥6 months since completion of primary radiotherapy	5. More than three enhancing lesions present on MRI or multi-focal recurrence
6. Prior history of standard dose, conventionally fractionated CNS radiotherapy	6. IDH1/2 mutant tumours on original pathology (to avoid unbalance between arms).
7. As a minimum patients will have completed at least two weeks of temozolomide, concurrent with their original radiotherapy.	7. GBM with known features of PXA, BRAF mutations or 1p19q co-deletion (on original pathology or updated pathology if available)
• Up to and including three enhancing lesions:<br>○ In cases of a single recurrent enhancing lesion:<br>▪ Predicted re-irradiation GTV<75cm^3 and<br>▪ Maximum diameter of enhancing disease must be ≤6cm.<br>○ In cases of multiple (i.e. two or three) discrete recurrent enhancing lesions:<br>▪ The total (i.e. combined) predicted re-irradiation GTV must be <50cm^3 and lesions must be clustered in a similar brain region such that PTVs are anticipated to be adjacent or overlapping and<br>▪ Maximum diameter of the combined enhancing disease, across all enhancing lesions (including any gaps between), must be ≤6cm.	8. Prior invasive malignancy (except non-melanomatous skin cancer), unless disease free for a minimum of one year.

For peer review only

8. Karnofsky Performance Status 70+	9. Severe active co-morbidity making patient unsuitable for chemotherapy or re-irradiation
9. Adequate hematologic, renal, and hepatic function	10. Prior allergic reaction to nitrosoureas
10. Patients who have had surgery for first recurrence may also be included provided there is residual enhancing disease on the immediate post-operative MRI or if enhancing disease develops on subsequent follow-up imaging, provided no prior systemic therapy or re-irradiation for recurrence has been given.	11. Any recognised genetic syndromes causing sensitivity to radiotherapy
11. Patients must have recovered from the prior effects of therapy	12. Patient unwilling/ unable to attend for follow up in the radiotherapy centre
12. Medical history and physical examination, including CNS examination, must be performed within 14 days prior to randomisation.	13. Contra-indication to MRI or gadolinium
13. Female participants of child-bearing potential must agree to be pregnancy screened.	14. Previous radiotherapy dose distribution unavailable
14. Patients must be able to swallow oral medication.	15. Previous systemic therapy or re-irradiation for recurrent GBM
15. Patients must be able to provide study-specific informed consent.	16. Spinal or infratentorial disease
16. Age 18 or over.	
17. Patients should be able to start treatment within 21 days of randomisation and must start within 28 days of randomisation.	

Coeliac disease is usually considered a contra-indication to lomustine as there is wheat within the capsule. For patients with recurrent GBM, the individual clinician may decide whether or not this is a relevant exclusion criteria.

For peer review only - http://bmjopen.bmj.com/site/about/guidelines.xhtml

Delete this line, then print on Trust/Hospital headed paper

Participant ID:	Initials:
Date of Birth:	NHS/Hospital Number:
EudraCT Number: 2019-004053-91	Principal Investigator:

BRIOChe

Please initial each box

PARTICIPANT CONSENT FORM

1. I confirm that I have read and understood the information sheet for the above study and have had the opportunity to ask questions.

2. I understand that my participation in this study is voluntary and that I am free to withdraw at any time without my medical care or legal rights being affected. I understand that even if I withdraw from the above study, the data and samples collected from me will be used in analysing the results of the study and in some cases further information about any unwanted effects of my treatment may need to be collected by the study team.

3. I understand that my healthcare records may be looked at by authorised individuals from the study team, regulatory bodies or Sponsor in order to check that the study is being carried out correctly.

4. I agree to allow any information or results arising from this study to be used for healthcare and/or further medical research upon the understanding that my identity will remain anonymous wherever possible. This includes the use of my radiotherapy data and imaging data for future analysis.

5. I understand that if I lose capacity, further information about any unwanted effects of my treatment may need to be collected by the study team.

6. If selected, I agree to take part in a semi-structured interview on my experiences of participating in the trial and my health-related quality of life, where a member of the research team may approach me directly to schedule a suitable day and time. I agree to these interviews being audio recorded.

7. I agree to consent to my anonymised trial data to be viewed if required by 3rd parties outside the UK / EU etc.

8. I agree to a copy of this Consent Form being sent to the Clinical Trials Research Unit.

9. I agree that my GP, or any other doctor treating me, will be notified of my participation in this study.

10. I agree to take part in the study.

The following points are **OPTIONAL.**

Title: Consent for potential tissue collection and use in future research (if you do not wish to give this permission do not initial the boxes – you can still participate in the trial)

Please initial each box

	Yes	No
I give my permission for stored tumour tissue sample that was removed during my operation and was not needed for routine diagnosis and treatment to be collected and used for future translational research purposes as described in the information sheet for the above trial if this becomes relevant in the future.		
I understand how the tissue sample will be collected, that giving samples is voluntary and that I'm free to withdraw my approval for use of the samples at any time without giving a reason and without my medical care or legal rights being affected.		

SAMPLE

For peer review only

Patient:
Signature……………………………………………………………………………

Name (block capitals)………………………………………………………………

Date…………………………………………………………………………………

Investigator:

I have explained the study to the above named patient and he/she has indicated his/her willingness to participate.

Signature……………………………………………………………………………

Name (block capitals)………………………………………………………………

Date…………………………………………………………………………………

(If used)Translator:

Signature……………………………………………………………………………

Name (block capitals)………………………………………………………………

Date…………………………………………………………………………………

(1 copy for patient; 1 for the CTRU; 1 held in patient notes;

SAMPLE
For peer review only

SPIRIT

STANDARD PROTOCOL ITEMS: RECOMMENDATIONS FOR INTERVENTIONAL TRIALS

SPIRIT 2013 Checklist: Recommended items to address in a clinical trial protocol and related documents*

Section/item	Item No	Description	Addressed on page number
**Administrative information**			
Title	1	Descriptive title identifying the study design, population, interventions, and, if applicable, trial acronym	1 ___________
Trial registration	2a	Trial identifier and registry name. If not yet registered, name of intended registry	4, 23__________
	2b	All items from the World Health Organization Trial Registration Data Set	Many__________
Protocol version	3	Date and version identifier	23 ___________
Funding	4	Sources and types of financial, material, and other support	4, 26__________
Roles and responsibilities	5a	Names, affiliations, and roles of protocol contributors	1, 26 _________
	5b	Name and contact information for the trial sponsor	26 ___________
	5c	Role of study sponsor and funders, if any, in study design; collection, management, analysis, and interpretation of data; writing of the report; and the decision to submit the report for publication, including whether they will have ultimate authority over any of these activities	26 ___________
	5d	Composition, roles, and responsibilities of the coordinating centre, steering committee, endpoint adjudication committee, data management team, and other individuals or groups overseeing the trial, if applicable (see Item 21a for data monitoring committee)	20, 21 ________

For peer review only

Introduction

Background and rationale	6a	Description of research question and justification for undertaking the trial, including summary of relevant studies (published and unpublished) examining benefits and harms for each intervention	6,7, 8_____
	6b	Explanation for choice of comparators	6, 7__________
Objectives	7	Specific objectives or hypotheses	11 _________
Trial design	8	Description of trial design including type of trial (e.g., parallel group, crossover, factorial, single group), allocation ratio, and framework (e.g., superiority, equivalence, non-inferiority, exploratory)	9 ___________

Methods: Participants, interventions, and outcomes

Study setting	9	Description of study settings (e.g., community clinic, academic hospital) and list of countries where data will be collected. Reference to where list of study sites can be obtained	9 ________
Eligibility criteria	10	Inclusion and exclusion criteria for participants. If applicable, eligibility criteria for study centres and individuals who will perform the interventions (e.g., surgeons, psychotherapists)	13, Table 1, Supplementary file 1
Interventions	11a	Interventions for each group with sufficient detail to allow replication, including how and when they will be administered	15__________
	11b	Criteria for discontinuing or modifying allocated interventions for a given trial participant (e.g., drug dose change in response to harms, participant request, or improving/worsening disease)	15, 16_________
	11c	Strategies to improve adherence to intervention protocols, and any procedures for monitoring adherence (e.g., drug tablet return, laboratory tests)	14,15 ________
	11d	Relevant concomitant care and interventions that are permitted or prohibited during the trial	N/A________
Outcomes	12	Primary, secondary, and other outcomes, including the specific measurement variable (e.g., systolic blood pressure), analysis metric (e.g., change from baseline, final value, time to event), method of aggregation (e.g., median, proportion), and time point for each outcome. Explanation of the clinical relevance of chosen efficacy and harm outcomes is strongly recommended	11 _____

Participant timeline	13	Time schedule of enrolment, interventions (including any run-ins and washouts), assessments, and visits for participants. A schematic diagram is highly recommended (see Figure)	Table 2, Figure 1
Sample size	14	Estimated number of participants needed to achieve study objectives and how it was determined, including clinical and statistical assumptions supporting any sample size calculations	14_______
Recruitment	15	Strategies for achieving adequate participant enrolment to reach target sample size	12______

Methods: Assignment of interventions (for controlled trials)

Allocation:

Sequence generation	16a	Method of generating the allocation sequence (e.g., computer-generated random numbers), and list of any factors for stratification. To reduce predictability of a random sequence, details of any planned restriction (eg, blocking) should be provided in a separate document that is unavailable to those who enrol participants or assign interventions	13, 14 __________
Allocation concealment mechanism	16b	Mechanism of implementing the allocation sequence (e.g., central telephone; sequentially numbered, opaque, sealed envelopes), describing any steps to conceal the sequence until interventions are assigned	13, 14 ___________
Implementation	16c	Who will generate the allocation sequence, who will enrol participants, and who will assign participants to interventions	11, 12, 13______
Blinding (masking)	17a	Who will be blinded after assignment to interventions (e.g., trial participants, care providers, outcome assessors, data analysts), and how	N/A _________
	17b	If blinded, circumstances under which unblinding is permissible, and procedure for revealing a participant's allocated intervention during the trial	N/A__________

Methods: Data collection, management, and analysis

Data collection methods	18a	Plans for assessment and collection of outcome, baseline, and other trial data, including any related processes to promote data quality (e.g., duplicate measurements, training of assessors) and a description of study instruments (e.g., questionnaires, laboratory tests) along with their reliability and validity, if known. Reference to where data collection forms can be found, if not in the protocol	10, 11, (Figure 1)

	18b	Plans to promote participant retention and complete follow-up, including list of any outcome data to be collected for participants who discontinue or deviate from intervention protocols	16__________
Data management	19	Plans for data entry, coding, security, and storage, including any related processes to promote data quality (e.g., double data entry; range checks for data values). Reference to where details of data management procedures can be found, if not in the protocol	20 _________
Statistical methods	20a	Statistical methods for analysing primary and secondary outcomes. Reference to where other details of the statistical analysis plan can be found, if not in the protocol	18,19______
	20b	Methods for any additional analyses (e.g., subgroup and adjusted analyses)	18,19_______
	20c	Definition of analysis population relating to protocol non-adherence (e.g., as randomised analysis), and any statistical methods to handle missing data (e.g., multiple imputation)	18 _________
**Methods: Monitoring**			
Data monitoring	21a	Composition of data monitoring committee (DMC); summary of its role and reporting structure; statement of whether it is independent from the sponsor and competing interests; and reference to where further details about its charter can be found, if not in the protocol. Alternatively, an explanation of why a DMC is not needed	21 __________
	21b	Description of any interim analyses and stopping guidelines, including who will have access to these interim results and make the final decision to terminate the trial	21, 23 ________
Harms	22	Plans for collecting, assessing, reporting, and managing solicited and spontaneously reported adverse events and other unintended effects of trial interventions or trial conduct	17 ____
Auditing	23	Frequency and procedures for auditing trial conduct, if any, and whether the process will be independent from investigators and the sponsor	20_________
**Ethics and dissemination**			
Research ethics approval	24	Plans for seeking research ethics committee/institutional review board (REC/IRB) approval	23 ___________

Protocol amendments	25	Plans for communicating important protocol modifications (e.g., changes to eligibility criteria, outcomes, analyses) to relevant parties (e.g., investigators, REC/IRBs, trial participants, trial registries, journals, regulators)	23_____________
Consent or assent	26a	Who will obtain informed consent or assent from potential trial participants or authorised surrogates, and how (see Item 32)	11 ____________
	26b	Additional consent provisions for collection and use of participant data and biological specimens in ancillary studies, if applicable	N/A___________
Confidentiality	27	How personal information about potential and enrolled participants will be collected, shared, and maintained in order to protect confidentiality before, during, and after the trial	20 _________
Declaration of interests	28	Financial and other competing interests for principal investigators for the overall trial and each study site	26 ____________
Access to data	29	Statement of who will have access to the final trial dataset, and disclosure of contractual agreements that limit such access for investigators	20 _________
Ancillary and post-trial care	30	Provisions, if any, for ancillary and post-trial care, and for compensation to those who suffer harm from trial participation	N/A _________
Dissemination policy	31a	Plans for investigators and sponsor to communicate trial results to participants, healthcare professionals, the public, and other relevant groups (e.g., via publication, reporting in results databases, or other data sharing arrangements), including any publication restrictions	23 _____
	31b	Authorship eligibility guidelines and any intended use of professional writers	23 ___________
	31c	Plans, if any, for granting public access to the full protocol, participant-level dataset, and statistical code	20__________
**Appendices**			
Informed consent materials	32	Model consent form and other related documentation given to participants and authorised surrogates	Supplementary fie 2 __________
Biological specimens	33	Plans for collection, laboratory evaluation, and storage of biological specimens for genetic or molecular analysis in the current trial and for future use in ancillary studies, if applicable	_N/A__________

For peer review only - http://bmjopen.bmj.com/site/about/guidelines.xhtml

*It is strongly recommended that this checklist be read in conjunction with the SPIRIT 2013 Explanation & Elaboration for important clarification on the items. Amendments to the protocol should be tracked and dated. The SPIRIT checklist is copyrighted by the SPIRIT Group under the Creative Commons "Attribution-NonCommercial-NoDerivs 3.0 Unported" license.

For peer review only
